# Supplementary material for: Development of a novel therapy for systolic heart failure
Source: EMBO Mol Med. 2025 Aug 4;17(9):2332–53. doi: 10.1038/s44321-025-00284-6 (PMC12423297; doi:10.1038/s44321-025-00284-6)
Supplement: Supplementary file 1 — Appendix [file 44321_2025_284_MOESM1_ESM.pdf]

Table of contents **Page No.**

MEDICINAL CHEMISTRY

Chemical synthesis of lead compound C26 2

Chemical synthesis of C26 analogues 3

Chemistry experimental: General materials and methods 8

APPENDIX TABLE S1: P VALUES 32

Chemical synthesis of lead compound C26

The synthesis of 3-(pyrrolidin-2-yl) isoxazole **2** was undertaken across seven steps from commercially available proline **5** (Supplementary Scheme 1). Di-*tert*-butyl dicarbonate (Boc) protection of the proline nitrogen was followed by reduction of the resulting carbamate **6** with boron trifluoride diethyl etherate. Alcohol **7** was then oxidised under Parikh-Doering oxidation conditions to the corresponding aldehyde **8**. Subsequent conversion to oxime **9** using hydroxylamine hydrochloride proceeded smoothly allowing the formation of chlorooxime **10** by reaction with *N*-chlorosuccinimide. Addition of triethylamine to **10** in the presence of 3-methyl-1-butyne via 1,3-dipolar cycloaddition, followed by removal of the protecting group with trifluoroacetic acid resulted in formation of the desired isoxazole product **2**.

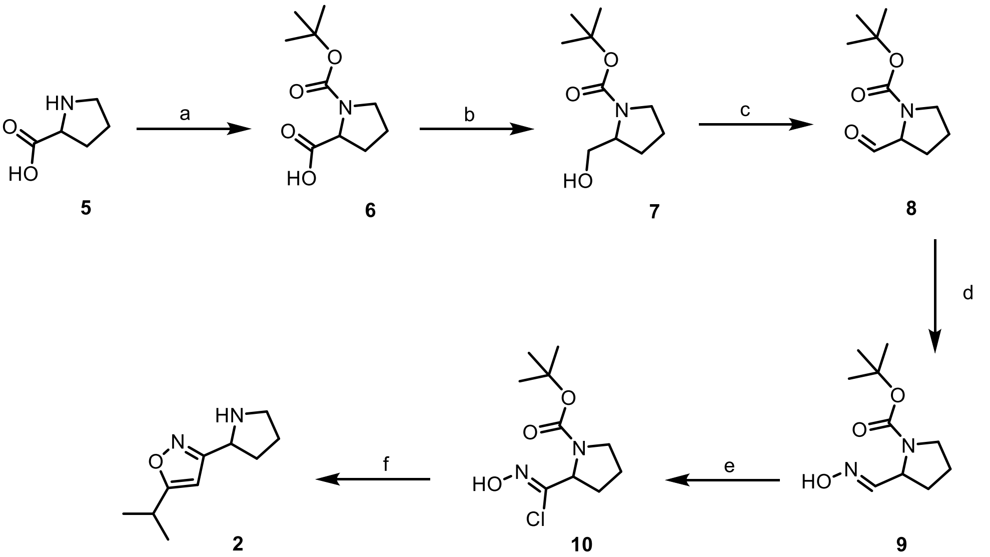

Appendix Figure S1 Synthesis of the isoxazole fragment **8**. Reagents and conditions: (a) Boc_2O, Et_3N, CH_2Cl_2, r.t., 16 h, 94%; (b) $NaBH_4$, BF_3•Et_2O, THF, 0 °C to r.t., 16 h, 85%; (c) Et_3N, DMSO, Pyr•SO_3, CH_2Cl_2, 0 °C to r.t., 2 h, 85%; (d) NH_2OH•HCl, $NaHCO_3$, MeOH, 0 °C to r.t., 16 h, 95%; (e) 4 M HCl in dioxane, NCS, DMF, 0 °C to r.t., 4 h, 84%; (f) $(CH_3)_2CHCCH$, Et_3N, CH_2Cl_2, r.t., 16 h then TFA, CH_2Cl_2, 0 °C, 3 h, 62% over 2 steps.

Two separate strategies were devised and tested for the synthesis of the hit compound **C26** using isoxazole **2** (Supplementary Scheme 2). The first method relied on alkylation of the secondary amine of isoxazole **2** with an alkyl halide. Initial attempts to produce the *N*-ethyl indole variant resulted in a highly unstable product upon reduction of the ester. Methyl 1*H*-indole-3-carboxylate **11** was first Boc-protected at the indole nitrogen using di-*tert*-butyl dicarbonate and subsequently reduced using DIBAL to give alcohol **12**. Alcohol **12** was successfully converted to alkyl halide **1** using phosphorus tribromide. Compound **1** was then used for the *N*-alkylation of isoxazole **2** to form the pyrrolidine C-N

bond, followed by removal of the carbamate protecting group to give free indole analogue **4**. Alkylation of the indole nitrogen of **4** with bromoethane was then undertaken which gave the lead compound **C26**.

The second method aimed to remove the two steps of Boc-protection/deprotection by employing reductive amination to form the methylene-pyrrolidine C-N bond. Vilsmeier formylation of indole **13** resulted in the *1H*-indole-3-carbaldehyde **14**, which was smoothly *N*-alkylated by bromoethane to give ethylindole **15**. Finally, reaction of isoxazole **2** with indole **15** in the presence of sodium triacetoxyborohydride gave the hit compound **C26.**

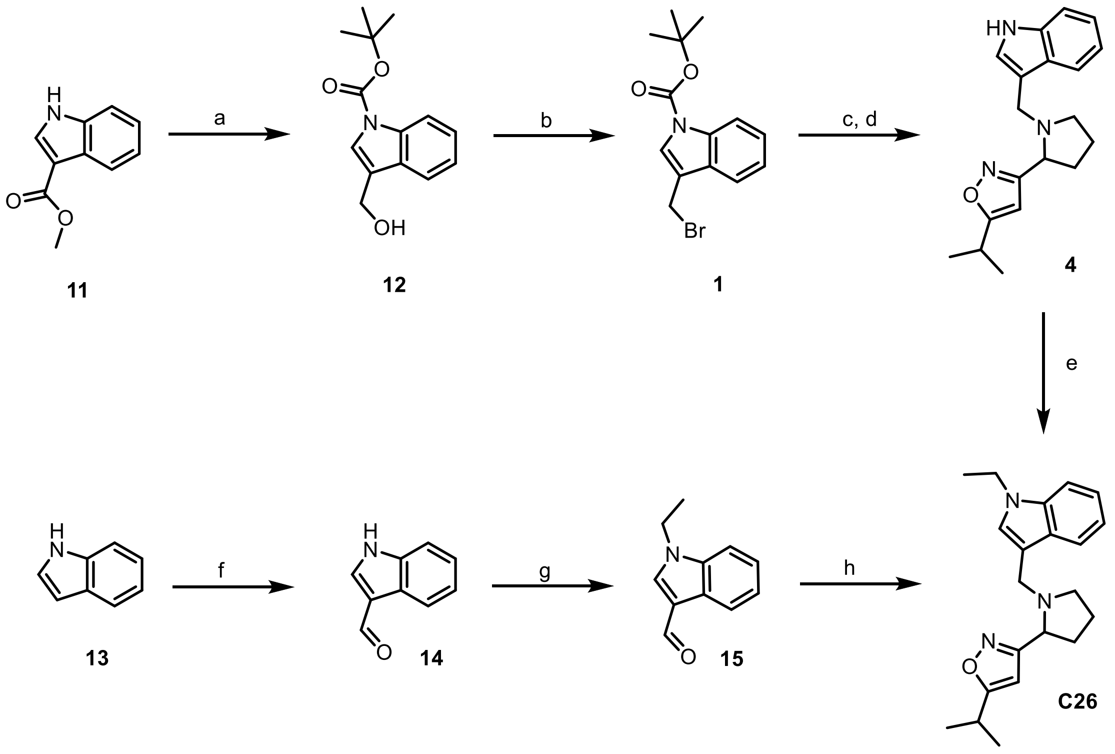

Appendix Figure S2 Synthesis of **C26** via indoles **4** or **15**. Reagents and conditions: (a) Boc_2O, DMAP, PhMe, r.t., 2 h, then 1 M DIBAL in PhMe, CH_2Cl_2, -78 °C to 0 °C, 2 h, 67% over two steps; (b) PBr_3, Et_2O, 0 °C, 1 h, 95%; (c) Isoxazole **2**, Et_3N, CH_2Cl_2, reflux, 16 h (d) TFA, CH_2Cl_2, 0 °C, 3 h, 12% over steps c and d; (e) EtBr, KOH, DMF, r.t., 2 h, 60%; (f) DMF, $POCl_3$, CH_2Cl_2, reflux, 3.5 h, 65%; (g) EtBr, KOH, DMF, r.t., 2 h, 94%; (h) Isoxazole **2**, $NaBH(OAc)_3$, $(CHCl)_2$, r.t., 1.5 h, 56%.

Chemical synthesis of C26 analogues

The first area targeted for derivatisation was the isoxazole ring system (Supplementary Scheme 3). The alkyl substituent was varied by using different alkynes for the generation of isoxazoles **16-19**. Alkylation of the isoxazoles at the pyrrolidine nitrogen using the previously synthesised alkyl indole **1** gave intermediates **20-23**. Installation of ethyl group was achieved using bromoethane producing

isoxazole analogues **24-27**. Unfortunately, an initial attempt for synthesis with a disubstituted alkyne was unsuccessful but this result was not further explored.

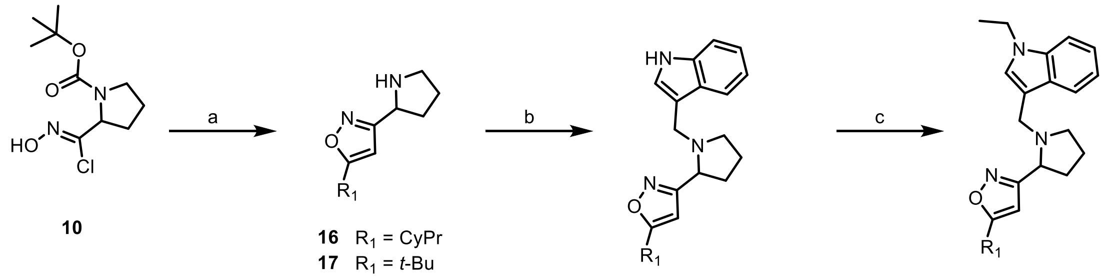

10

16 R_1 = CyPr
17 R_1 = *t*-Bu
18 R_1 = *i*-Bu
19 R_1 = Ph

20 R_1 = CyPr
21 R_1 = *t*-Bu
22 R_1 = *i*-Bu
23 R_1 = Ph

24 R_1 = CyPr
25 R_1 = *t*-Bu
26 R_1 = *i*-Bu
27 R_1 = Ph

Appendix Figure S3 Synthesis of **C26** analogues with alternate substitutions to the isopropyl group. Reagents and conditions: (a) Alkyne, Et_3N, CH_2Cl_2, r.t., 16 h, then TFA, CH_2Cl_2, 0 °C, 3 h, 52-73% over two steps; (b) Indole **1,** Et_3N, CH_2Cl_2, r.t., 16 h then TFA, CH_2Cl_2, 0 °C, 3 h, 13-29% over two steps; (c) EtBr, KOH, DMF, r.t., 2 h, 92-95% over two steps.

Focus turned to optimisation of the *N*-alkyl substituent of the indole (Supplementary Scheme 4). Analogue generation was achieved by variation of the alkylating agent used. Propyl analogue **28** was synthesised by the addition of bromopropane to indole **4** in the presence of potassium hydroxide. Unfortunately, no products were isolated when other alkylating agents were used, even when conditions were varied. Failure was thus thought to be due to the sensitivity of compound **4**, so formyl indole **14** was then used as a substrate for alkylation with reductive amination following. Addition of a methyl group to form analogue **29** was achieved by heating to 130 °C with dimethylcarbonate in the presence of potassium carbonate. Ethoxymethylene and cyanomethylene analogues **30** and **31** were synthesised using chloromethylethylether and bromoacetonitrile, respectively, in the presence of sodium hydride. Benzyl analogue **32** was synthesised using a combination of sodium hydride and benzyl bromide. The alkylated indoles **29-32** were then subjected to reductive amination with isoxazole **2** giving *N*-alkyl analogues **33-36**.

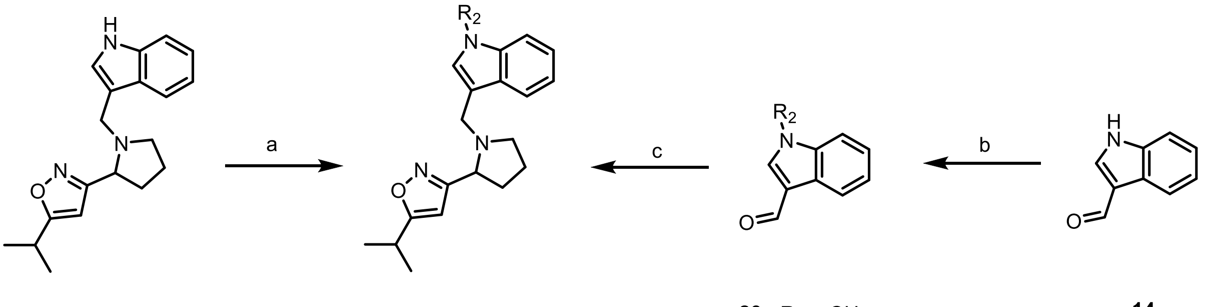

4

28 R_2 = $CH_2CH_2CH_3$
33 R_2 = CH_3
34 R_2 = CH_2OEt
35 R_2 = CH_2CN
36 R_2 = Bn

29 R_2 = CH_3
30 R_2 = CH_2OEt
31 R_2 = CH_2CN
32 R_2 = Bn

14

Appendix Figure S4 Synthesis of **C26** analogues with alternate *N*-substitutions of the indole. Reagents and conditions: (a) $CH_3CH_2CH_2Br$, KOH, DMF, r.t., 2 h, 75% (b) K_2CO_3/DMF or NaH/THF, alkylating agent, 67-93% (c) Isoxazole **2,** $NaBH(OAc)_3$, $(CHCl)_2$, r.t., 1.5 h, 27-50%.

The change of the indole scaffold to the related indazole scaffold was next explored. As indazole is unable to undergo Vilsmeier formylation, new chemistry was needed to access this analogue. Starting with methyl 1*H*-indazole-3-carboxylate **37**, alkylation with bromoethane in the presence of potassium *tert*-butoxide gave the 1-ethyl product **38** (Supplementary Scheme 5). The ester was then reduced to the corresponding alcohol **39** using DIBAL followed by oxidation with manganese dioxide to give aldehyde **40**. The aldehyde was then subject to reductive amination with isoxazole **11** giving indazole analogue **41**.

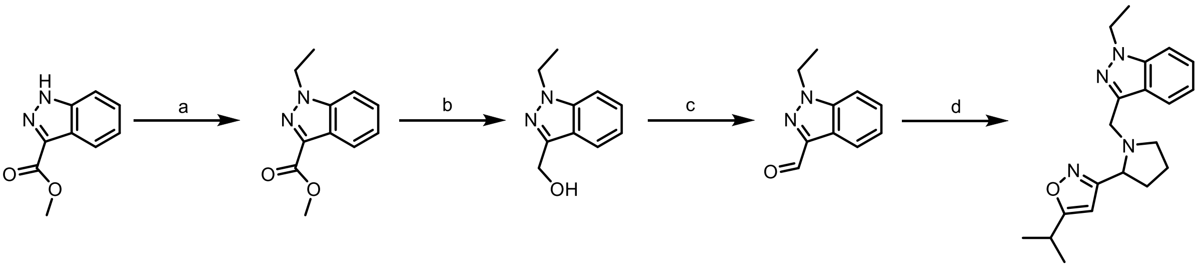

37 **38** **39** **40** **41**

Appendix Figure S5 Synthesis of indazole analogue of **C26**. Reagents and conditions: (a) EtBr, tBuOK, THF, r.t., 26 h, 29%; (b) 1 M DIBAL in PhMe, CH_2Cl_2, -78 °C to 0 °C, 2 h, 58% (c) MnO_2, CH_2Cl_2, r.t., 2 h, 79% (d) Isoxazole **2**, $NaBH(OAc)_3$, $(CHCl)_2$, r.t., 1.5 h, 16%.

The linker region was then targeted for derivatisation. Ester **11** was taken and alkylated using previously established conditions to give alkyl indole **42** (Supplementary Scheme 6). The ester was subsequently hydrolysed using potassium hydroxide to give the free acid **43**. Following conversion into an acid chloride using oxalyl chloride, reaction with isoxazole **8** gave amide analogue **44**.

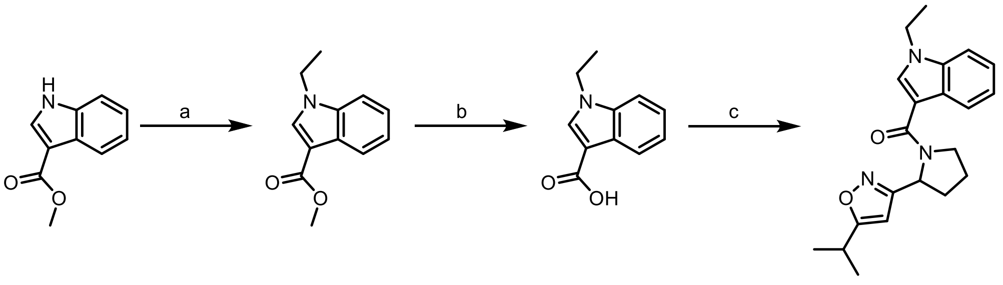

11 **42** **43** **44**

Appendix Figure S6 Synthesis of carbonyl linker analogue of **C26**. Reagents and conditions: (a) EtBr, KOH, DMF, r.t., 2 h, 98%; (b) KOH, THF, reflux, 16 h, 64%; (c) $(COCl)_2$, DMF, 0 °C to room temperature, 16 h, then isoxazole **2**, NEt_3, room temperature, 16 h, 94% over two steps.

Attention then turned to the central pyrrolidine ring. The use of enantiopure L-proline as a starting point allowed selective synthesis of the *S* analogue of C26 (Supplementary Scheme 7). Following the previously established conditions, D-proline **45** was protected with di-*tert*-butyl dicarbonate and the resultant carbamate **46** was reduced giving alcohol **47**. Oxidation was again completed using Parikh-Doering conditions and the aldehyde **48** turned into the oxime **49** then chlorooxime **50**. This allowed synthesis of the R-isoxazole **51** by 1,3-dipolar cycloaddition and deprotection of the nitrogen. Finally, reductive amination between indole **15** and isoxazole **51** produced the *R*-enantiomer of compound **C26**.

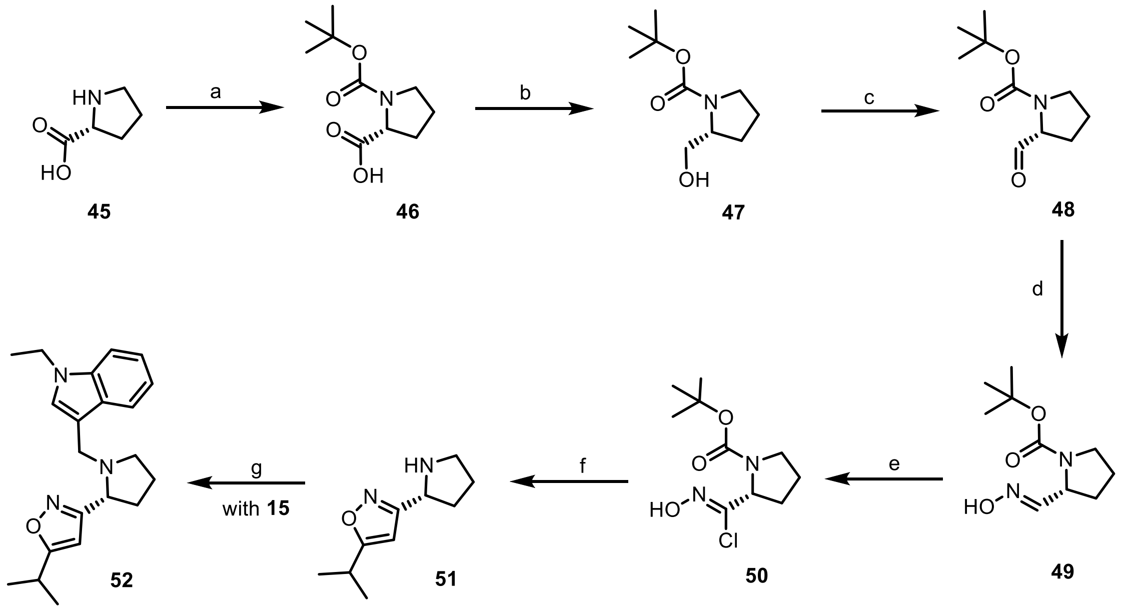

Appendix Figure S7 Synthesis of the *R* enantiomer of **C26**. Reagents and conditions: (a) Boc_2O, Et_3N, CH_2Cl_2, r.t., 16 h, 91%; (b) $NaBH_4$, BF_3•Et_2O, THF, 0 °C to r.t., 16 h, 60%; (c) Et_3N, DMSO, Pyr•SO_3, CH_2Cl_2, 0 °C to r.t., 2 h, 85%; (d) NH_2OH•HCl, $NaHCO_3$, MeOH, 0 °C to r.t., 16 h, 81%; (e) 4 M HCl in dioxane, NCS, DMF, 0 °C to r.t., 16 h, 81%; (f) $(CH_3)_2CHCCH$, Et_3N, CH_2Cl_2, r.t., 16 h then TFA, CH_2Cl_2, 0 °C, 3 h, 42% over 2 steps; (g) Compound **15**, $NaBH(OAc)_3$, $(CHCl)_2$, r.t., 1.5 h, 53%.

Ring expansion of the pyrrolidine was then conducted based on the synthetic strategies already established. Piperidine-2-carboxylic acid **53** was taken and protected at the nitrogen with di-*tert*-butyl decarbonate (Supplementary Scheme 8). The acid **54** was subsequently reduced to give alcohol **55**. Parikh-Doering oxidation successfully formed aldehyde **56** which was subsequently transformed into oxime **57** then chlorooxime **58**. A cycloaddition with 3-methyl-1-butyne and chlorooxime **58** formed the corresponding isoxazole. Reductive amination was then conducted using indole **15** giving ring expanded analogue **BR43**.

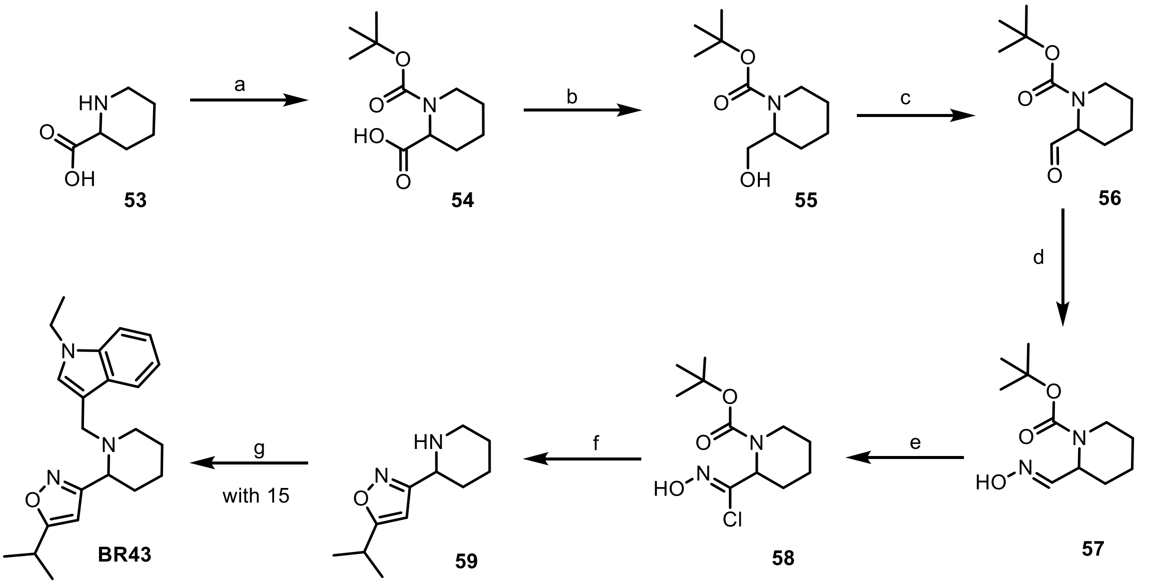

Appendix Figure S8 Synthesis of **BR43** as the piperidine analogue of **C26**. Reagents and conditions: (a) Boc_2O, Et_3N, CH_2Cl_2, r.t., 16 h, 94%; (b) $NaBH_4$, BF_3•Et_2O, THF, 0 °C to r.t., 16 h, 60%; (c) Et_3N, DMSO, Pyr•SO_3, CH_2Cl_2, 0 °C to r.t., 2 h, 30%; (d) NH_2OH•HCl, $NaHCO_3$, MeOH, 0 °C to r.t., 16 h, 93%; (e) 4 M HCl in dioxane, NCS, DMF, 0 °C to r.t., 4 h, 72%; (f) $(CH_3)_2CHCCH$, Et_3N, CH_2Cl_2, r.t., 16 h then TFA, CH_2Cl_2, 0 °C, 3 h, 51% over 2 steps; (g) Compound **15**, $NaBH(OAc)_3$, $(CHCl)_2$, r.t., 1.5, 26%.

For the generation analogues with chloro substituents on the 5- and 6-posiiton of the indole ring, formylation of the relevant commercially available indoles **60** and **61** was undertaken by Vilsmeier reagent using phosphoryl chloride and dimethylformamide. Boc protection followed to give compounds **62** and **63**. Reductive amination then indole deprotection produced **64** and **65**, with alkylation completing the synthesis of *N*-ethyl analogues **66** and **67**.

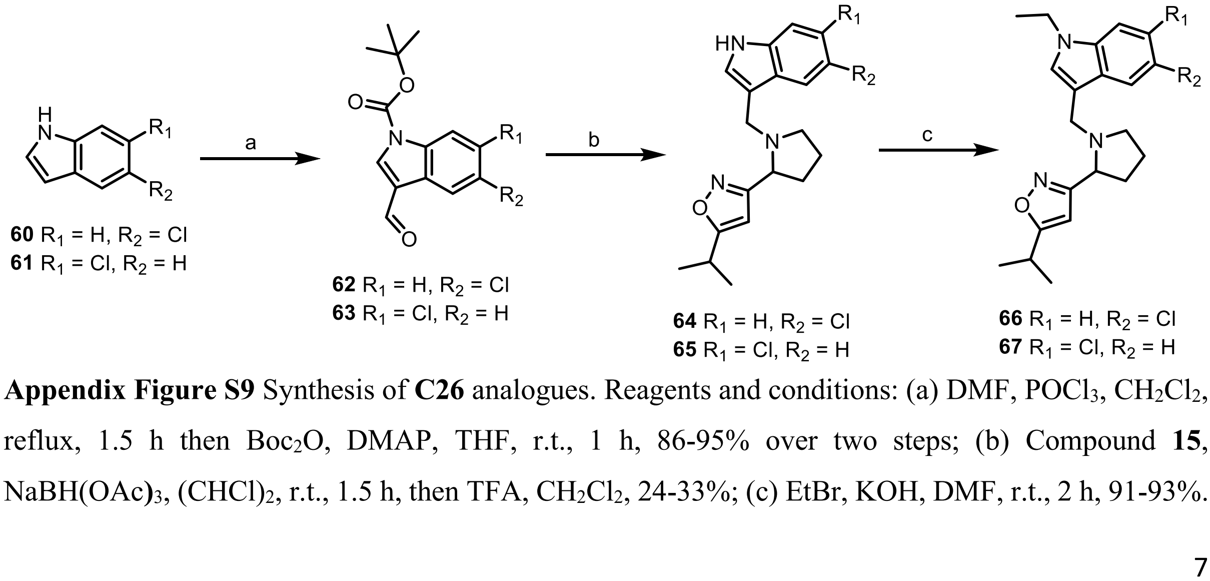

Appendix Figure S9 Synthesis of **C26** analogues. Reagents and conditions: (a) DMF, $POCl_3$, CH_2Cl_2, reflux, 1.5 h then Boc_2O, DMAP, THF, r.t., 1 h, 86-95% over two steps; (b) Compound **15**, $NaBH(OAc)_3$, $(CHCl)_2$, r.t., 1.5 h, then TFA, CH_2Cl_2, 24-33%; (c) EtBr, KOH, DMF, r.t., 2 h, 91-93%.

Chemistry Experimental

General Materials and Methods

All glassware used in moisture sensitive reactions was oven dried at 140 °C and then cooled under vacuum prior to use. Anhydrous dichloromethane, diethyl ether, dimethylformamide, tetrahydrofuran and toluene used were obtained from Caledon Laboratories and dried using an Innovative Technologies Solvent Purification System with dual columns packed with solvent appropriate drying agents. Dry triethylamine was distilled from and stored over potassium hydroxide. Anhydrous dimethyl sulfoxide was dried over 4 Å molecular sieves. Anhydrous methanol was purchased from Sigma-Aldrich and used without further purification. All other solvents, reagents and starting materials were purchased as reagent-grade from commercial sources and used without further purification. All reactions were carried out at room temperature unless otherwise noted.

All reactions were monitored by thin layer chromatography (TLC), unless otherwise noted. Analytical Thin Layer Chromatography (TLC) was performed on Merck kieselgel 60 F254 plates aluminium backed plates and visualised using a 254 nm UV lamp or by staining with a ninhydrin stain consisting of ninhydrin (0.2 g), acetic acid (0.5 mL) and water (4.5 mL) in ethanol (100 mL), a *p*-anisaldehyde stain consisting of p-anisaldehyde (3.7 mL), acetic acid (1.5 mL) and concentrated sulfuric acid (5 mL) in ethanol (135 mL), a vanillin stain consisting of vanillin (15 g), conc. sulfuric acid (2.5 mL) in ethanol (250 mL), a DNP stain consisting of 2,4-dinitrophenylhydrazine (12 g), conc. sulfuric acid (60 mL), water (80 mL) in ethanol (200 mL) and a bromocresol green stain consisting of bromocresol green (0.04 g) and 0.1M sodium hydroxide added until basic in ethanol (100 mL).

Flash chromatography was performed on silica gel (Davisil® LC60Å 40-63 microns. Melting points were, measured on a Reichert Thermopan microscope hot stage apparatus and are uncorrected.

Solvents for NMR spectroscopy were purchased from Cambridge Isotope Laboratories. NMR spectra were recorded on a Bruker Avance 400 at 400.13 MHz for 1H nuclei, 100.62 MHz for 13C nuclei and at 376.47 MHz for 19F nuclei or a Bruker AV-500 at 500.19 MHz for 1H nuclei and at 125.78 MHz for 13C nuclei. For 1H NMR spectra the residual CHCl3 peak (7.26 ppm), residual DMSO peak (2.50 ppm), residual MeOH peak (3.31 ppm) were used as internal standards. For 13C NMR spectroscopy the central peak of the CDCl3 triplet (77.23 ppm), the central peak of the DMSO heptet (39.52 ppm) or the MeOH heptet (49.00 ppm) were used as internal standards. Chemical shifts are reported as [illegible] values in parts per million (ppm). In reporting spectral data, the following abbreviations have been used: s, singlet; br s, broad singlet; d, doublet; t, triplet; q, quartet; m, multiplet. Coupling constants (*J*) are reported in Hz to the nearest 0.5 Hz.

Low-resolution electrospray ionization (ESI) mass spectra were recorded on a Bruker Daltronics Esquire 6000 Ion Trap mass spectrometer in a mixture of methanol or acetonitrile with 0.1% formic acid at 40 eV cone voltage in positive mode, unless otherwise stated. High-resolution electrospray ionization mass spectra were recorded on an Agilent 6500 series QTOF LC/MS mass spectrometer. All

data were acquired, and reference mass corrected via a dual-spray electrospray ionization (ESI) source, in positive mode, unless otherwise stated.

Analytical reverse phase HPLC was performed a Shimadzu LC-20AB Prominence Liquid Chromatography system fitted with a Phenomenex® Luna C18(2) 100 Å column (250 mm × 4.6 mm, 10 µm), using a binary solvent system; solvent A: water with 0.1% (v/v) trifluoroacetic acid; solvent B: acetonitrile with 0.1% (v/v) trifluoroacetic acid. Gradient elution was performed using two methods. Method A: A gradient of 90% solvent A to 90% solvent B over 26 minutes with a flow rate of 1 mL/min, monitored at 254 nm. Method B: A gradient of 90% solvent A to 90% solvent B over 12 minutes with a flow rate of 1 mL/min, monitored at 254 nm. Preparatory reverse phase HPLC was performed on the same system with a Phenomenex® Jupiter C18 300 Å column (250 mm × 10.0 mm, 10 µm), using the same solvent system specified above. Gradient elution was performed using a gradient of 90% solvent A to 10% solvent B over 60 minutes with a flow rate of 2 mL/min, monitored at 254 nm.

General Procedure 1: To a solution of chlorooxime (4 mmol, 1 eq.) and alkyne (12 mmol, 3 eq.) in dichloromethane (0.15M) at 0 °C was added triethylamine (4 mmol, 1 eq.) and the reaction allowed to stir at room temperature for 16 hours. Upon completion the solvent was removed *in vacuo* and the remaining slurry dissolved into ethyl acetate (30 mL), washed with water (3 × 30 mL), brine (1 × 30 mL), dried with sodium sulfate and the solvent removed *in vacuo*. The resultant material was then dissolved into dichloromethane (0.2M), cooled to 0 °C, trifluoroacetic acid (12 mmol, 3 eq.) added and the reaction was stirred for 3 hours. Upon completion the reaction was neutralised with saturated sodium carbonate (20 mL) and the aqueous phase extracted with dichloromethane (2 × 20 mL). The combined organic extracts were then dried with sodium sulfate and the solvent removed *in vacuo*.

General Procedure 2: To a solution of amine (2.50 mmol, 1 eq.) and triethylamine (7.50 mmol, 3 eq.) in dichloromethane (0.4M) was added the alkyl halide (2.50 mmol, 1 eq.) and the mixture heated to reflux for 16 hours. Upon completion the solvent was removed *in vacuo* and the remaining slurry dissolved into ethyl acetate (20 mL), washed with water (3 × 10 mL), brine (1 × 10 mL), dried with sodium sulfate and the solvent removed *in vacuo*. The resultant material was then dissolved into dichloromethane (0.1M) and trifluoroacetic acid (50 mmol, 20 eq.) added at 0 °C and the reaction left to stir for 3 hours. Upon completion the mixture was neutralised with saturated potassium carbonate and the solution extracted with dichloromethane (2 × 10 mL). The combined organic extracts were then washed with water (1 × 10 mL), brine (1 × 10 mL), dried with sodium sulfate and the solvent removed *in vacuo*.

General Procedure 3: To a solution of indole (1.00 mmol, 1 eq.) and potassium hydroxide (3.00 mmol, 3 eq.) in dimethylformamide (1M) was added bromoethane (2.00 mmol, 2 eq.) and the reaction left to stir for 2 hours. Upon completion the reaction was diluted with ethyl acetate (10 mL), washed with water (5 × 5 mL), brine (1 × 5 mL), dried with sodium sulfate and the solvent removed *in vacuo*.

General Procedure 4: To a solution of amine (1 mmol, 1 eq.) and aldehyde (1 mmol, 1 eq.) in dichloroethane (0.3M) under an atmosphere of nitrogen was added sodium triacetoxyborohydride (1.4 mmol, 1.4 eq.) and the reaction left to stir for 1.5 hours. Upon completion the reaction was neutralised with saturated sodium bicarbonate and the mixture extracted with ethyl acetate (3 × 10 mL). The combined organic extracts were then washed with brine (1 × 15 mL), dried with magnesium sulfate and the solvent removed *in vacuo*.

(*S*)-3-(1-((1-Ethyl-1*H*-indol-3-yl) methyl) pyrrolidin-2-yl)-5-isopropylisoxazole C26

General procedure 3 using indole **4** (0.100 g, 0.32 mmol) gave alkyl indole **C26** as an orange-brown oil (0.070 g, 60%) while general procedure 4 using isoxazole **2** (0.180 g, 1 mmol) and indole **15** gave product **C26** as an orange-brown oil (0.189 g, 56%). **^{1}H NMR** (400 MHz, $CDCl_3$): δ 7.65 (1H, d, *J* = 8.0 Hz), 7.32 (1H, d, *J* = 8.0 Hz), 7.21 (1H, t, *J* = 8.0 Hz), 7.11 (1H, t, *J* = 8.0 Hz), 7.06 (1H, s), 6.10 (1H, s), 4.14 (2H, q, *J* = 7.5 Hz), 4.04 (1H, d, *J* = 13.5 Hz), 3.71 (1H, t, *J* = 8.0 Hz), 3.58 (1H, d, *J* = 13.5 Hz), 3.21-3.15 (1H, m), 3.08 (1H, sept., *J* = 8.0 Hz), 2.41 (1H, q, *J* = 8.0 Hz), 2.24-2.15 (1H, m), 1.98-1.85 (2H, m), 1.85-1.74 (1H, m), 1.46 (3H, t, *J* = 8.0 Hz), 1.34 (6H, d, *J* = 7.0 Hz) ppm; **^{13}C NMR** (100 MHz, $CDCl_3$): δ 178.7 (C_q), 167.1 (C_q), 136.1 (C_q), 128.7 (C_q), 126.9 (CH), 121.4 (CH), 119.6 (CH), 118.9 (CH), 111.1 (C_q), 109.3 (CH), 97.2, (CH) 59.4 (CH), 53.6 (CH_2), 47.8 (CH_2), 40.9 (CH_2), 32.2 (CH_2), 27.3 (CH), 22.8 (CH_2), 21.0 (CH_3), 15.6 (CH_3) ppm; **HRMS** (ESI): *m/z* $[M+H]^+$ calculated for $C_{21}H_{28}N_3O^+$ 338.2227, found 338.2233; **RP-HPLC**: t_R 15.3 mins (Method A); **Chiral NP-HPLC**: t_R 8.5 mins.

(*tert*-Butoxycarbonyl)-*L*-proline 6

To a solution of *L*-proline **5** (20.0 g, 174 mmol, 1 eq.) in dry dichloromethane (400 mL) under an atmosphere of nitrogen was added di-*tert*-butyl dicarbonate (41.8 g, 191 mmol, 1.1 eq.) and dry triethylamine (26.6 mL, 191 mmol, 1.1 eq.). The reaction was left to stir for 16 hours at room temperature. Upon completion the reaction was neutralised with 1M hydrochloric acid, the organic phase separated and washed with brine (1 × 100 mL), dried with magnesium sulfate and the solvent removed *in vacuo* gave the Boc protected proline **6** as a white solid (35.0 g, 94%). **^{1}H NMR** (400 MHz, $CDCl_3$, exists as 1:1 mixture of isomers): δ 10.71 (2H, br. s), 4.34-4.33 (1H, m), 4.23-4.20 (1H, m), 3.54-3.34 (4H, m), 2.28-1.84 (8H, m), 1.46 (9H, s), 1.40 (9H, s) ppm; **^{13}C-NMR** (100 MHz, $CDCl_3$, exists as a 1:1 mixture of isomers): δ 178.0 (C_q), 175.0 (C_q), 156.1 (C_q), 154.2 (C_q), 81.0 (C_q), 80.2 (C_q), 59.4 (2 × CH), 47.1 (CH_2), 46.5 (CH_2), 31.1 (CH_2), 29.1 (CH_2), 28.6 (CH_3), 28.5 (CH_3), 24.5 (CH_2), 23.8 (CH_2) ppm; **HRMS** (ESI): *m/z* $[M+Na]^+$ calculated for $C_{10}H_{17}NNaO_4^+$ 238.1050, found 238.1054.

***tert*-Butyl (*S*)-2-(hydroxymethyl) pyrrolidine-1-carboxylate 7**

To a solution of Boc protected proline **6** (35.0 g, 163 mmol, 1 eq.) in tetrahydrofuran (113 mL) and cooled to 0 °C was added sodium borohydride (9.87 g, 261 mmol, 1.6 eq.) over a 30-minute period

followed by boron trifluoride diethyl etherate (40.2 mL, 326 mmol, 2 eq.). The vessel was allowed to warm to room temperature and stirred for 16 hours. Upon completion the reaction was diluted with saturated sodium hydroxide (226 mL) and the solvent removed *in vacuo*. The remaining aqueous phase was then extracted with ethyl acetate (4 × 100 mL). The combined organic phase was then washed with saturated potassium carbonate (2 × 100 mL), brine (1 × 100 mL), dried with sodium sulfate and the solvent removed *in vacuo* gave the alcohol **7** as a white solid (27.9 g, 85%). **^{1}H NMR** (400 MHz, $CDCl_3$, exists as a 0.2:1 mixture of isomers): δ 4.73 (2H, s, maj. + min.), 3.98-3.87 (1H, m, maj.), 3.86-3.76 (1H, m, min.), 3.62-3.50 (4H, m, maj. + min.), 3.45-3.36 (2H, m, maj. + min.), 3.31-3.22 (2H, m, maj. + min.), 2.03-1.90 (2H, m, maj. + min.), 1.89-1.66 (4H, m, maj. + min.) 1.61-1.47 (1H, m, maj.), 1.43 (19H, s, maj. + min.) ppm; **^{13}C-NMR** (100 MHz, $CDCl_3$): δ 157.2 (C_q), 80.3 (C_q), 67.7 (CH_2), 60.3 (CH), 47.7 (CH_2), 28.8 (CH_2), 28.6 (CH_3), 24.2 (CH_2) ppm; **HRMS** (ESI): *m/z* $[M+Na]^+$ calculated for $C_{10}H_{19}NNaO_3^+$ 224.1257, found 224.1259.

***tert*-Butyl (*S*)-2-formylpyrrolidine-1-carboxylate 8**

To a solution of Boc protected prolinol **7** (8.00 g, 39.8 mmol, 1 eq.) in dichloromethane (86 mL) under an atmosphere of nitrogen at 0 °C was added triethylamine (17.5 mL, 125 mmol, 3.15 eq.) and dimethylsulfoxide (14.1 mL, 199 mmol, 5 eq.). Pyridine sulfur trioxide complex (19.0 g, 119 mmol, 3 eq.) was then added portion wise and the reaction left to stir for 1 hour before being warmed to room temperature and stirred for another hour. Upon completion the reaction was diluted with brine (50 mL) and the solvent removed *in vacuo*. The aqueous phase was then extracted with ethyl acetate (3 × 50 mL). The combined organic phase was then washed with brine (1 × 100 mL), dried with sodium sulfate and the solvent removed *in vacuo* to give a yellow-brown oil. The yellow oil was then dissolved into a 50:50 mixture of ethyl acetate and hexane then passed through a plug of silica, followed again by solvent removal *in vacuo* to give the aldehyde **8** as a light-yellow oil (6.74 g, 85%). **^{1}H NMR** (400 MHz, $CDCl_3$, exists as 0.6:1 a mixture of isomers): δ 9.50 (1H, s, min), 9.41 (1H, s maj.), 4.16-4.13 (1H, m, min.), 4.02-3.98 (1H, m, maj.), 3.55-3.38 (4H, m, maj. + min.), 2.12-1.80 (8H, m, maj. + min.), 1.43 (9H, s, min.), 1.38 (9H, s, maj.) ppm; **^{13}C NMR** (100 MHz, $CDCl_3$, exists as a 0.6:1 mixture of isomers): δ 200.8 (CH, min.), 200.5 (CH, maj.), 155.0 (C_q, min.), 154.1 (C_q, maj.), 80.7 (C_q, maj.), 80.3 (C_q, min.), 65.1 (CH, maj.), 65.0 (CH, min.), 47.0 (CH_2, min.), 46.8 (CH_2, maj.), 28.5 (CH_3, min.), 28.4 (CH_3, maj.), 28.1 (CH_2, maj.), 26.8 (CH_2, min.), 24.7 (CH_2, min.), 24.1 (CH_2, maj.) ppm; **HRMS** (ESI): *m/z* $[M+Na]^+$ calculated for $C_{10}H_{17}NNaO_3^+$ 222.1106, found 222.1111.

***tert*-Butyl (*S*)-2-((hydroxyimino)methyl) pyrrolidine-1-carboxylate 9**

To a solution of Boc protected prolinal **8** (12.1 g, 60.6 mmol, 1 eq.) in methanol (61 mL) at 0 °C was added hydroxylamine hydrochloride (4.63 g, 66.6 mmol, 1.1 eq.) and sodium bicarbonate (6.62 g, 78.8 mmol, 1.3 eq.). The reaction was then warmed to room temperature and stirred for 16 hours. Upon completion the solvent was removed *in vacuo* and the resultant slurry dissolved into brine (30 mL) then

extracted with ethyl acetate (4 × 60 mL). The combined organic phase was then passed through a silica plug, dried with sodium sulfate and the solvent removed *in vacuo* to give oxime **9** as a clear oil (12.3 g, 95%). **^{1}H NMR** (400 MHz, MeOD, exists as a 0.5:1 mixture of isomers): δ 7.27 (1H, br s, maj.), 6.64 (1H, br s, min.), 4.90-4.81 (1H, m, min.), 4.40-4.27 (1H, m, maj.), 3.45-3.36 (4H, m, maj. + min.), 2.31-2.20 (1H, m, min.), 2.17-2.02 (1H, m, maj.), 2.00-1.79 (6H, m, maj. + min.), 1.47 (9H, s, min.), 1.44 (9H, s, maj.) ppm; **^{13}C NMR** (100 MHz, MeOD, exists as a 0.5:1 mixture of isomers): δ 156.2 (C_q, maj. + min.), 154.5 (CH, min.), 151.5 (CH, maj.), 81.1 (C_q, maj. + min.), 57.5 (CH, maj.), 54.1 (CH, min.), 47.6 (CH_2, maj. + min.), 31.3 (CH_2, maj. + min.), 24.5 (CH_2, maj. + min.) ppm; **HRMS** (ESI): *m/z* $[M+Na]^+$ calculated for $C_{10}H_{18}N_2NaO_3^+$ 237.1210, found 237.1209.

***tert*-Butyl (*S*)-2-(chloro(hydroxyimino)methyl) pyrrolidine-1-carboxylate 10**

To a solution of oxime **9** (12.3 g, 57.5 mmol, 1 eq.) in dimethylformamide (81 mL) at 0 °C was added 4M HCl in dioxane (1.44 mL, 5.75 mmol, 0.1 eq.) followed by *N*-chlorosuccinimide (8.45 g, 63.3 mmol, 1.1 eq.) whilst maintaining the temperature at 0 °C. The reaction was then stirred at room temperature for 4 hours. Upon completion the reaction was diluted with water (200 mL) and extracted with ethyl acetate (4 × 100 mL). The combined organic extracts were then washed with water (1 × 200 mL), brine (1 × 200 mL), dried with sodium sulfate and the solvent removed *in vacuo* to give chlorooxime **10** as a pale-yellow solid (12.0 g, 84%). **^{1}H NMR** (400 MHz, $CDCl_3$, exists as a 0.5:1 mixture of isomers): δ 9.54 (1H, br s, maj.), 9.41 (1H, br s, min.), 4.72-4.61 (1H, m, min.), 4.56-4.46 (1H, m, maj.), 3.54-3.40 (4H, m, maj. + min.), 2.20-2.09 (2H, m, maj. + min.), 2.08-1.90 (4H, m, maj. + min.), 1.90-1.76 (2H, m, maj. + min.), 1.44 (9H, s, min.), 1.39 (9H, s, maj.) ppm; **^{13}C NMR** (100 MHz, $CDCl_3$, exists as a 0.5:1 mixture of isomers): δ 154.59 (C_q, min.), 154.54 (C_q, maj.), 141.1 (C_q, maj.), 140.6 (C_q, min.), 80.7 (C_q, maj.), 80.6 (C_q, min.), 61.2 (CH, maj.), 60.7 (CH, min.), 47.3 (CH_2, min.), 46.6 (CH_2, maj.), 31.0 (CH_2, maj.), 30.4 (CH_2, min.), 28.6 (CH_3, min.), 28.5 (CH_3, maj.), 23.9 (CH_2, min.), 23.0 (CH_2, maj.) ppm.

(*S*)-5-Isopropyl-3-(pyrrolidin-2-yl) isoxazole hydrochloride 2

General procedure 1 using chlorooxime **10** (7.00 g, 28.1 mmol) and 3-methyl-1-butyne gave isoxazole **2** as a brown oil (3.14 g, 62% over 2 steps). **^{1}H NMR** (400 MHz, $CDCl_3$): δ 5.90 (1H, s), 4.26-4.22 (1H, m), 3.61 (2H, br s), 3.10-3.05 (1H, m), 3.01-2.94 (2H, m), 2.16-2.11 (1H, m), 1.89-1.77 (3H, m), 1.24 (6H, d, J = 7.0 Hz) ppm; **^{13}C NMR** (100 MHz, $CDCl_3$): δ 179.0 (C_q), 166.5 (C_q), 93.3 (CH), 54.6 (CH), 46.7 (CH_2), 31.8 (CH_2), 27.3 (CH), 25.4 (CH_2), 20.9 (CH_3) ppm; **HRMS** (ESI): *m/z* $[M+H]^+$ calculated for $C_{10}H_{17}N_2O^+$ 181.1335, found 181.1339; **RP-HPLC**: t_R 10.6 mins (Method A).

***tert*-Butyl 3-(hydroxymethyl)-1*H*-indole-1-carboxylate 12**

To a solution of methyl 1*H*-indole-3-carboxylate **11** (2.00 g, 11.4 mmol, 1 eq.) in dry toluene (45.7 mL) under an atmosphere of nitrogen was added di-*tert*-butyl dicarbonate (2.74 g, 12.6 mmol, 1.1 eq.) then

dimethylaminopyridine (0.138 g, 1.14 mmol, 0.1 eq.) and the reaction left to stir for 2 hours. The reaction was then diluted with ethyl acetate (50 mL), washed with water (5 × 25 mL) then brine (1 × 25 mL), dried with magnesium sulfate and the solvent removed *in vacuo* to give the protected indole. The protected indole (5.00 g, 18.2 mmol, 1 eq.) was then dissolved in dry dichloromethane (55 mL) under an atmosphere of nitrogen at -78 °C and a 1M DIBAL in toluene (27.3 mL, 27.3 mmol, 1.5 eq.) solution was added dropwise over 10 minutes at which point the reaction was warmed to 0 °C and left to stir for 2 hours. Upon completion, the reaction was partitioned with saturated potassium sodium tartrate (80 mL) and left to stir overnight to separate. Once clear separation of the layers was achieved the aqueous phase was extracted with dichloromethane (3 × 100 mL). The combined organic extracts were then washed with brine (1 × 150 mL), dried with sodium sulphate and the solvent removed *in vacuo*. The resultant solid was purified by flash column chromatography in 20% ethyl acetate:hexane giving alcohol **12** as a yellow solid (3.00 g, 67%). **^{1}H NMR** (400 MHz, $CDCl_3$): δ 8.14 (1H, d, *J* = 8.0 Hz), 7.62 (1H, d, *J* = 8.0 Hz), 7.55 (1H, s), 7.33 (1H, t, *J* = 8.0 Hz), 7.24 (1H, t, *J* = 8.0 Hz), 4.79 (2H, s), 1.67 (9H, s) ppm; **^{13}C NMR** (100 MHz, $CDCl_3$): δ 149.9 (C_q), 135.9 (C_q), 129.4 (C_q), 124.8 (CH), 123.8 (CH), 122.8 (CH), 120.7 (C_q), 119.5 (CH), 115.4 (CH), 83.9 (C_q), 57.2 (CH_2), 28.3 (CH_3) ppm.

***tert*-Butyl 3-(bromomethyl)-1*H*-indole-1-carboxylate 1**

To a solution of Boc indole alcohol **12** (7.62 g, 27 mmol, 1 eq.) in dry diethyl ether (160 mL) under an atmosphere of nitrogen at 0 °C was added phosphorus tribromide (7.32 g, 27 mmol, 1 eq.) and the reaction maintained at this temperature for 2 hours. Upon completion the reaction was warmed and saturated sodium bicarbonate (300 mL) added. The organic phase was then washed with water (1 × 150 mL), brine (1 × 150 mL) and the solvent removed *in vacuo* resulting in alkyl halide **1** as a tan solid (7.95 g, 95%). **^{1}H NMR** (400 MHz, $CDCl_3$): δ 8.20-8.14 (1H, m), 7.70-7.68 (2H, m), 7.40-7.30 (2H, m), 4.69 (2H, s), 1.67 (9H, s) ppm; **^{13}C NMR** (100 MHz, $CDCl_3$): δ 149.5 (C_q), 135.9 (C_q), 128.9 (C_q), 125.24 (CH), 125.19 (CH), 123.1 (CH), 119.5 (CH), 117.4 (C_q), 115.7 (CH), 84.4 (C_q), 28.4 (CH_3), 24.7 (CH_2) ppm.

(*S*)-3-(1-((1*H*-Indol-3-yl)methyl)pyrrolidin-2-yl)-5-isopropylisoxazole 4

General procedure 2 using isoxazole **2** (1.50 g, 8.32 mmol) and indole **1** gave product **4** as a brown solid (0.488 g, 19% over 2 steps). **^{1}H NMR** (400 MHz, $CDCl_3$): δ 8.61 (1H, s), 7.66 (1H, d, *J* = 8.0 Hz), 7.32 (1H, d, *J* = 8.0 Hz), 7.18 (1H, t, *J* = 8.0 Hz), 7.12 (1H, t, *J* = 8.0 Hz), 7.07 (1H, s), 6.11 (1H, s), 4.07 (1H, d, *J* = 13.5 Hz), 3.73 (1H, t, *J* = 8.0 Hz), 3.60 (1H, d, *J* = 13.5 Hz), 3.20-3.14 (1H, m), 3.08 (1H, sept., *J* = 8.0 Hz), 2.41 (1H, q, *J* = 8.0 Hz), 2.24-2.15 (1H, m), 1.97-1.83 (2H, m), 1.83-1.72 (1H, m), 1.34 (6H, d, *J* = 8.0 Hz) ppm; **^{13}C NMR** (100 MHz, $CDCl_3$): δ 178.9 (C_q), 167.1 (C_q), 136.4 (C_q), 128.1 (C_q), 124.2 (CH), 121.9 (CH), 119.4 (CH × 2), 112.2 (C_q), 111.3 (CH), 97.3 (CH), 59.4 (CH), 53.5 (CH_2), 47.8 (CH_2), 32.2 (CH_2), 27.4 (CH), 22.5 (CH_2), 21.0 (CH_3) ppm; **HRMS** (ESI): *m/z* $[M+H]^+$ calculated for $C_{19}H_{24}N_3O^+$ 310.1914, found 310.1913; **RP-HPLC**: t_R 13.4 mins (Method A).

1*H*-Indole-3-carbaldehyde 14

To a solution of dry dimethylformamide (19.8 mL, 256 mmol, 6 eq.) in dry dichloromethane (213 mL) under an atmosphere of nitrogen at 0 °C was added a solution of phosphorus oxychloride (11.9 mL, 128 mmol, 3 eq.) in dry dichloromethane (76.8 mL). The mixture was then heated to reflux for 30 minutes, upon which a solution of indole **13** (5.00 g, 42.7 mmol, 1 eq.) in dry dichloromethane (213 mL) was added and the reaction left to reflux for a further 3 hours. Upon completion the reaction was cooled, water (100 mL) was added, and the pH adjusted to 8 using potassium carbonate. The mixture was then extracted with chloroform (3 × 50 mL). The combined organic phases were then washed with brine (1 × 75 mL), dried with sodium sulfate and the solvent removed *in vacuo* to give formyl indole **14** as a light-yellow solid (4.02 g, 65%). **^{1}H NMR** (400 MHz, d_6-DMSO): δ 12.14 (1H, br s), 9.95 (1H, s), 8.28 (1H, s), 8.13-8.10 (1H, m), 7.53-7.51 (1H, m), 7.29-7.19 (2H, m) ppm; **^{13}C NMR** (100 MHz, d_6-DMSO): δ 185.0 (CH), 138.4 (CH), 137.1 (Cq), 124.2 (C_q), 123.5 (CH), 122.1 (CH), 120.8 (CH), 118.2 (C_q), 112.4 (CH) ppm; **HRMS** (ESI): *m/z* $[M+H]^+$ calculated for $C_9H_8NO^+$ 168.0420, found 168.0421.

1-Ethyl-1*H*-indole-3-carbaldehyde 15

General procedure 3 using compound **14** (1.00 g, 6.89 mmol) gave indole **15** as a dark yellow solid (1.12 g, 94%). **^{1}H NMR** (400 MHz, $CDCl_3$): δ 9.92 (1H, s), 8.25-8.22 (1H, m), 7.70 (1H, s), 7.34-7.32 (1H, m), 7.29-7.24 (2H, m), 4.17 (2H, q, J = 7.5 Hz), 1.48 (3H, t, J = 7.5 Hz) ppm; **^{13}C NMR** (100 MHz, $CDCl_3$): δ 184.4 (CH), 138.9 (C_q), 136.9 (CH), 125.3 (C_q), 123.7 (CH), 122.7 (CH), 121.8 (CH), 117.9(C_q), 110.0 (CH), 41.7 (CH_2), 14.8 (CH_3) ppm; **HRMS** (ESI): *m/z* $[M+Na]^+$ calculated for $C_{11}H_{11}NNaO^+$ 196.0733, found 196.0732.

(*S*)-5-Cyclopropyl-3-(pyrrolidin-2-yl) isoxazole hydrochloride 16

General procedure 1 using chlorooxime **10** (1.25 g, 5.03 mmol) and ethynylcyclopropane gave isoxazole **16** as a brown oil (0.508 g, 57% over 2 steps). **^{1}H NMR** (400 MHz, $CDCl_3$): δ 6.03 (1H, s), 4.79 (1H, t, J = 7.0 Hz), 3.47 (2H, t, J = 7.0 Hz), 2.48-2.42 (1H, m), 2.27-2.11 (3H, m), 206-1.98 (1H, m), 1.10-1.03 (2H, m), 0.97-0.93 (2H, m) ppm; **^{13}C NMR** (100 MHz, $CDCl_3$): δ 177.2 (C_q), 159.8 (C_q), 97.5 (CH), 55.1 (CH), 45.5 (CH_2), 30.3 (CH_2), 23.7 (CH_2), 8.9 (CH_2), 8.3 (CH) ppm; **HRMS** (ESI): *m/z* $[M+H]^+$ calculated for $C_{10}H_{15}N_2O^+$ 179.1179, found 179.1184; **RP-HPLC**: t_R 8.4 mins (Method A).

(*S*)-5-(*tert*-Butyl)-3-(pyrrolidin-2-yl) isoxazole hydrochloride 17

General procedure 1 using chlorooxime **10** (1.00 g, 4.02 mmol) and 3,3-dimethyl-1-butyne gave isoxazole **17** as a brown oil (0.410 g, 52% over 2 steps). **^{1}H NMR** (400 MHz, $CDCl_3$): δ 6.07 (1H, s), 4.81 (1H, t, J = 7.0 Hz), 3.48 (2H, t, J = 7.0 Hz), 2.50-2.41 (1H, m), 2.30-2.12 (3H, m), 1.31 (9H, s) ppm; **^{13}C NMR** (100 MHz, $CDCl_3$): δ 183.5 (C_q), 159.4 (C_q), 97.3 (CH), 55.1 (CH), 45.4 (CH_2), 33.1

(C_q), 30.3 (CH_2), 28.9 (CH_3), 23.7 (CH_2) ppm; **HRMS** (ESI): *m/z* $[M+H]^+$ calculated for $C_{11}H_{19}N_2O^+$ 195.1492, found 195.1499; **RP-HPLC**: t_R 14.7 mins (Method A).

(*S*)-5-Isobutyl-3-(pyrrolidin-2-yl) isoxazole hydrochloride 18

General procedure 1 using chlorooxime **10** (1.00 g, 4.02 mmol) and 4-methyl-1-pentyne gave isoxazole **18** as a brown oil (0.537 g, 69% over 2 steps). **^{1}H NMR** (400 MHz, $CDCl_3$): δ 6.01 (1H, s), 5.39 (2H, br s), 4.45 (1H, t, *J* = 7.0 Hz), 3.27-3.20 (1H, m), 3.18-3.14 (1H, m), 2.58 (2H, d, *J* = 7.0 Hz), 2.33-2.22 (1H, m), 2.06-1.91 (4H, m), 0.94 (6H, d, *J* = 7.0 Hz) ppm; **^{13}C NMR** (100 MHz, $CDCl_3$): δ 173.8 (C_q), 164.3 (C_q), 100.1 (CH), 54.9 (CH), 46.3 (CH_2), 35.9 (CH_2), 31.4 (CH_2), 27.8 (CH), 24.9 (CH_2), 22.5 (CH_3) ppm; **HRMS** (ESI): *m/z* $[M+H]^+$ calculated for $C_{11}H_{19}N_2O^+$ 195.1492, found 195.1498; **RP-HPLC**: t_R 9.5 mins (Method A).

(*S*)-5-Phenyl-3-(pyrrolidin-2-yl) isoxazole hydrochloride 19

General procedure 1 using chlorooxime **10** (1.00 g, 4.02 mmol) and phenylacetylene gave isoxazole 1**9** as a brown oil (0.630 g, 73% over 2 steps). **^{1}H NMR** (400 MHz, $CDCl_3$): δ 7.76-7.73 (2H, m), 7.47-7.38 (3H, m), 6.51 (1H, s), 4.35 (1H, t, *J* = 6.5 Hz), 3.18-3.12 (1H, m), 3.07-3.01 (1H, m), 2.26-2.17 (1H, m), 1.97-1.85 (3H, m) ppm; **^{13}C NMR** (100 MHz, $CDCl_3$): δ 170.0 (C_q), 168.0 (C_q), 130.2 (CH), 129.1 (CH), 127.7 (C_q), 125.9 (CH), 98.0 (CH), 54.7 (CH), 47.0 (CH_2), 32.1 (CH_2), 25.5 (CH_2) ppm; **HRMS** (ESI): *m/z* $[M+H]^+$ calculated for $C_{13}H_{15}N_2O^+$ 215.1179, found 215.1185; **RP-HPLC**: t_R 10.8 mins (Method A).

(*S*)-3-(1-((1*H*-Indol-3-yl) methyl) pyrrolidin-2-yl)-5-cyclopropylisoxazole 20

General procedure 2 using isoxazole **16** (0.508 g, 2.85 mmol) and indole **1** gave product **20** as a brown solid (0.215 g, 13% over 2 steps). **^{1}H NMR** (400 MHz, $CDCl_3$): δ 8.49 (1H, br s), 7.65 (1H, d, *J* = 8.0 Hz), 7.33 (1H, d, *J* = 8.0 Hz), 7.19 (1H, t, *J* = 8.0 Hz), 7.12 (1H, t, *J* = 8.0 Hz), 7.07 (1H, s), 6.04 (1H, s), 4.05 (1H, d, *J* = 13.5 Hz), 3.70 (1H, t, *J* = 8.5 Hz), 3.58 (1H, d, *J* = 13.5 Hz), 3.14 (1H, t, *J* = 8.5 Hz), 2.39 (1H, q, *J* = 8.5 Hz), 2.21-2.12 (1H, m), 2.09-2.00 (1H, m), 1.93-1.83 (2H, m), 1.81-1.70 (1H, m), 1.09-1.04 (2H, m), 1.00-0.94 (2H, m) ppm; **^{13}C NMR** (100 MHz, $CDCl_3$): δ 175.0 (C_q), 167.3 (C_q), 136.4 (C_q), 128.1 (C_q), 124.2 (CH), 121.9 (CH), 119.5 (CH), 119.4 (CH), 112.2 (C_q), 111.3 (CH), 97.0 (CH), 59.4 (CH), 53.4 (CH_2), 47.7 (CH_2), 32.1 (CH_2), 22.7 (CH_2), 8.6 (CH_2), 8.4 (CH) ppm; **HRMS** (ESI): *m/z* $[M+H]^+$ calculated for $C_{19}H_{22}N_3O^+$ 308.1757, found 308.1754; **RP-HPLC**: t_R 12.8 mins (Method A).

(*S*)-3-(1-((1*H*-Indol-3-yl) methyl) pyrrolidin-2-yl)-5-(*tert*-butyl)isoxazole 21

General procedure 2 using isoxazole **17** (0.410 g, 2.11 mmol) and indole **1** gave product **21** as a brown solid (0.158 g, 23% over 2 steps). **^{1}H NMR** (400 MHz, $CDCl_3$): δ 8.64 (1H, br s), 7.65 (1H, d, *J* = 8.0 Hz), 7.32 (1H, d, *J* = 8.0 Hz), 7.18 (1H, t, *J* = 8.0 Hz), 7.12 (1H, t, *J* = 8.0 Hz), 7.07 (1H, s), 6.08 (1H, s), 4.07 (1H, d, *J* = 13.5 Hz), 3.74 (1H, t, *J* = 8.5 Hz), 3.62 (1H, d, *J* = 13.5 Hz), 3.19 (1H, t, *J* = 8.5

Hz), 2.43 (1H, q, *J* = 8.5 Hz), 2.26-2.14 (1H, m), 1.99-1.84 (2H, m), 1.84-1.72 (1H, m), 1.37 (9H, s) ppm; **^{13}C NMR** (100 MHz, $CDCl_3$): δ 181.3 (C_q), 167.0 (C_q), 136.4 (C_q), 128.1 (C_q), 124.2 (CH), 121.8 (CH), 119.4 (CH), 119.3 (CH), 112.1 (C_q), 111.3 (CH), 96.7 (CH), 59.4 (CH), 53.6 (CH_2), 47.8 (CH_2), 32.9 (C_q), 32.2 (CH_2), 29.0 (CH_3), 22.8 (CH_2) ppm; **HRMS** (ESI): *m/z* $[M+H]^+$ calculated for $C_{20}H_{26}N_3O^+$ 324.2070, found 324.2072; **RP-HPLC**: t_R 14.2 mins (Method A).

(*S*)-3-(1-((1*H*-Indol-3-yl) methyl) pyrrolidin-2-yl)-5-isobutylisoxazole 22

General procedure 2 using isoxazole **18** (0.537 g, 2.76 mmol) and indole **1** gave product **22** as a brown oil (0.256 g, 29% over 2 steps). **^{1}H NMR** (400 MHz, $CDCl_3$): δ 8.50 (1H, s), 7.64 (1H, d, 8.0 Hz), 7.32 (1H, d, *J* = 8.0 Hz), 7.18 (1H, t, *J* = 8.0 Hz), 7.10 (1H, t, *J* = 8.0 Hz), 7.07 (1H, s), 6.16 (1H, s), 4.07 (1H, d, *J* = 13.5 Hz)m 3.72 (1H, t, *J* = 8.5 Hz), 3.55 (1H, d, *J* = 13.5 Hz), 3.14 (1H, t, *J* = 8.5 Hz), 2.65 (2H, d, *J* = 7.0 Hz), 2.39 (1H, q, *J* = 8.5 Hz), 2.23-2.16 (1H, m), 2.08 (1H, sept., *J* = 7.0 Hz), 1.95-1.83 (2H, m), 1.81-1.73 (1H, m), 1.01 (6H, d, *J* = 7.0 Hz) ppm; **^{13}C NMR** (100 MHz, $CDCl_3$): δ 173.0 (C_q), 167.1 (C_q), 136.4 (C_q), 128.1 (C_q), 124.1 (CH), 121.9 (CH), 119.44 (CH), 119.41 (CH), 112.3 (C_q), 111.3 (CH), 99.8 (CH), 59.5 (CH), 53.5 (CH_2), 47.8 (CH_2), 36.0 (CH_2), 32.2 (CH_2), 27.8 (CH), 22.7 (CH_2), 22.5 (CH_3), 22.5, ppm; **HRMS** (ESI): *m/z* $[M+H]^+$ calculated for $C_{20}H_{26}N_3O^+$ 324.2070, found 324.2067; **RP-HPLC**: t_R 14.6 mins (Method A).

(*S*)-3-(1-((1*H*-Indol-3-yl) methyl) pyrrolidin-2-yl)-5-phenylisoxazole 23

General procedure 2 using isoxazole **19** (0.630 g, 2.94 mmol) and indole **1** gave product **23** as a brown solid (0.208 g, 21% over 2 steps). **^{1}H NMR** (400 MHz, $CDCl_3$): δ 8.13 (1H, br s), 7.78-7.75 (2H, m), 7.67 (1H, d, *J* = 8.0 Hz), 7.49-7.42 (3H, m), 7.33 (1H, d, *J* = 8.0 Hz), 7.17 (1H, t, *J* = 8.0 Hz), 7.13 (1H, s), 7.11 (1H, d, *J* – 8.0 Hz), 6.65 (1H, s), 4.08 (1H, d, *J* = 13.5 Hz), 3.80 (1H, t, *J* = 8.0 Hz), 3.65 (1H, d, *J* = 13.5 Hz), 3.20 (1H, t, *J* = 8.0 Hz), 2.46 (1H, q, *J* = 8.0 Hz), 2.27-2.20 (1H, m), 1.98-1.89 (2H, m), 1.85-1.77 (1H, m) ppm; **^{13}C NMR** (100 MHz, $CDCl_3$): δ 170.0 (C_q), 167.9 (C_q), 136.4 (C_q), 130.2 (CH), 129.1 (CH), 128.1 (C_q), 127.9 (C_q), 126.0 (CH), 124.2 (CH), 122.2 (CH), 119.7 (CH), 119.5 (CH), 112.5 (C_q), 111.3 (CH), 98.1 (CH), 59.5 (CH), 53.6 (CH_2), 47.9 (CH_2), 32.3 (CH_2), 22.9 (CH_2) ppm; **HRMS** (ESI): *m/z* $[M+H]^+$ calculated for $C_{22}H_{22}N_3O^+$ 344.1757, found 344.1768; **RP-HPLC**: t_R 14.5 mins (Method A).

(*S*)-5-Cyclopropyl-3-(1-((1-ethyl-1*H*-indol-3-yl) methyl) pyrrolidin-2-yl) isoxazole 24

General procedure 3 using compound **20** (0.115 g, 0.374 mmol) gave analogue **24** as a brown oil (0.116 g, 92%). **^{1}H NMR** (400 MHz, $CDCl_3$): δ 7.64 (1H, d, *J* = 8.0 Hz), 7.31 (1H, d, *J* = 8.0 Hz), 7.21 (1H, t, *J* = 8.0 Hz), 7.11 (1H, t. *J* = 8.0 Hz), 7.06 (1H, s), 6.03 (1H, s), 4.14 (2H, q, *J* = 7.5 Hz), 4.03 (1H, d, *J* = 13.5 Hz), 3.69 (1H, t, *J* = 8.5 Hz), 3.57 (1H, d, *J* = 13.5 Hz), 3.16 (1H, t, *J* = 8.5 Hz), 2.40 (1H, q, *J* = 8.5 Hz), 2.21-2.13 (1H, m), 2.07-2.00 (1H, m), 1.96-1.82 (2H, m), 1.82-1.73 (1H, m), 1.45 (3H, t, *J*

= 7.5 Hz), 1.09-1.02 (2H, m), 1.00-0.93 (2H, m) ppm; **^{13}C NMR** (100 MHz, $CDCl_3$): δ 174.9 (C_q), 167.3 (C_q), 136.1 (C_q), 128.7 (C_q), 127.0 (CH), 121.4 (CH), 119.6 (CH), 119.0 (CH), 110.9 (C_q), 109.3 (CH), 96.9 (CH), 59.4 (CH), 53.4 (CH_2), 47.7 (CH_2), 40.9 (CH_2), 32.1 (CH_2), 22.7 (CH_2), 15.6 (CH_3), 8.5 (CH_2), 8.3 (CH) ppm; **HRMS** (ESI): *m/z* $[M+H]^+$ calculated for $C_{21}H_{26}N_3O^+$ 336.2070, found 336.2075; **RP-HPLC**: t_R 14.7 mins (Method A).

(*S*)-5-(*tert*-Butyl)-3-(1-((1-ethyl-1*H*-indol-3-yl) methyl) pyrrolidin-2-yl)isoxazole 25

General procedure 3 using compound **21** (0.158 g, 0.489 mmol) gave analogue **25** as a brown oil (0.161 g, 94%). **^{1}H NMR** (400 MHz, $CDCl_3$): δ 7.63 (1H, d, *J* = 8.0 Hz), 7.31 (1H, d, *J* = 8.0 Hz), 7.20 (1H, t, *J* = 8.0 Hz), 7.10 (1H, t, *J* = 8.0 Hz), 7.07 (1H, s), 6.07 (1H, s), 4.13 (2H, q, *J* = 7.5 Hz), 4.04 (1H, d, *J* = 13.5 Hz), 3.72 (1H, t, *J* = 8.5 Hz), 3.58 (1H, d, *J* = 13.5 Hz), 3.18 (1H, t, *J* = 8.5 Hz), 2.42 (1H, q, *J* = 8.5 Hz), 2.23-2.14 (1H, m), 1.97-1.85 (2H, m), 1.84-1.75 (1H, m), 1.45 (3H, t, *J* = 7.5 Hz), 1.36 (9H, s) ppm; **^{13}C NMR** (100 MHz, $CDCl_3$): δ 181.2 (C_q), 166.9 (C_q), 136.1 (C_q), 128.7 (C_q), 127.0 (CH), 121.4 (CH), 119.6 (CH), 119.0 (CH), 111.0 (C_q), 109.3 (CH), 96.6 (CH), 59.4 (CH), 53.6 (CH_2), 47.7 (CH_2), 40.9 (CH_2), 32.9 (C_q), 32.1 (CH_2), 29.1 (CH_3), 22.8 (CH_2), 15.6 (CH_3) ppm; **HRMS** (ESI): *m/z* $[M+H]^+$ calculated for $C_{22}H_{30}N_3O^+$ 352.2383, found 352.2390; **RP-HPLC**: t_R 16.1 mins (Method A).

(*S*)-3-(1-((1-Ethyl-1*H*-indol-3-yl) methyl) pyrrolidin-2-yl)-5-isobutylisoxazole 26

General procedure 3 using compound **22** (0.256 g, 0.792 mmol) gave analogue **26** as a brown oil (0.265 g, 95%). **^{1}H NMR** (400 MHz, $CDCl_3$): δ 7.64 (1H, d, *J* = 8.0 Hz), 7.31 (1H, d, *J* = 8.0 Hz), 7.21 (1H, t, *J* = 8.0 Hz), 7.09 (1H, t, *J* = 8.0 Hz), 7.05 (1H, s), 6.15 (1H, s), 4.13 (2H, q, *J* = 7.5 Hz), 4.05 (1H, d, *J* = 13.5 Hz), 3.71 (1H, t, *J* = 8.5 Hz), 3.53 (1H, d, *J* = 13.5 Hz), 3.15 (1H, t, *J* = 8.5 Hz), 2.65 (2H, d, *J* = 7.0 Hz), 2.39 (1H, q, *J* = 8.5 Hz), 2.24-2.16 (1H, m), 2.08 (1H, sept., *J* = 7.0 Hz), 1.95-1.84 (2H, m), 1.83-1.73 (1H, m), 1.45 (3H, t, *J* = 7.5 Hz), 1.01 (6H, d, *J* = 7.0 Hz) ppm; **^{13}C NMR** (100 MHz, $CDCl_3$): δ 172.8 (C_q), 167.1 (C_q), 136.1 (C_q), 128.7 (C_q), 126.9 (CH), 121.5 (CH), 119.6 (CH), 119.0(CH), 112.6 (C_q), 109.3 (CH), 99.8 (CH), 59.5 (CH), 53.5 (CH_2), 47.8 (CH_2), 40.9 (CH_2), 36.0 (CH_2), 32.2 (CH_2), 27.8 (CH), 22.7 (CH_2), 22.5 (CH_3), 15.6 (CH_3) ppm; **HRMS** (ESI): *m/z* $[M+H]^+$ calculated for $C_{22}H_{30}N_3O^+$ 352.2383, found 352.2389; **RP-HPLC**: t_R 16.6 mins (Method A).

(*S*)-3-(1-((1-Ethyl-1*H*-indol-3-yl) methyl) pyrrolidin-2-yl)-5-phenylisoxazole 27

General procedure 3 using compound **23** (0.208 g, 0.606 mmol) gave analogue **27** as a brown oil (0.210 g, 93%). **^{1}H NMR** (400 MHz, $CDCl_3$): δ 7.79-7.75 (2H, m), 7.68 (1H, d, *J* = 8.0 Hz), 7.48-7.41 (3H, m), 7.30 (1H, d, *J* = 8.0 Hz), 7.21 (1H, t, *J* = 8.0 Hz), 7.17 (1H, s), 7.13 (1H, t, *J* = 8.0 Hz), 6.76 (1H, s), 4.18 (1H, d, *J* = 13.5 Hz), 4.12 (2H, q, *J* = 7.5 Hz), 3.96 (1H, t, *J* = 7.5 Hz), 3.81 (1H, d, *J* = 13.5 Hz), 3.36 (1H, t, *J* = 7.5 Hz), 2.60 (1H, q, *J* = 7.5 Hz), 2.34-2.23 (1H, m), 2.12-1.98 (2H, m), 1.90-1.78 (1H, m), 1.42 (3H, t, *J* = 7.5 Hz) ppm; **^{13}C NMR** (100 MHz, $CDCl_3$): δ 170.2 (C_q), 166.5 (C_q), 136.0

(C_q), 130.2 (CH), 129.0 (CH), 128.7 (C_q), 127.9 (CH), 127.6 (C_q), 125.9 (CH), 121.6 (CH), 119.42 (CH), 119.36 (CH), 109.5 (CH), 109.0 (C_q), 98.4 (CH), 59.5 (CH), 53.3 (CH_2), 47.4 (CH_2), 41.0 (CH_2), 31.8 (CH_2), 22.5 (CH_2), 15.5 (CH_3) ppm; **HRMS** (ESI): *m/z* $[M+H]^+$ calculated for $C_{24}H_{26}N_3O^+$ 372.2070, found 372.2077; **RP-HPLC**: t_R 16.1 mins (Method A).

(*S*)-5-Isopropyl-3-(1-((1-propyl-1*H*-indol-3-yl) methyl) pyrrolidin-2-yl) isoxazole 28

General procedure 3 using product **4** (0.070 g, 0.23 mmol, 1 eq.) and bromopropane gave product **28** as a brown oil (0.061 g, 75%). **^{1}H NMR** (400 MHz, $CDCl_3$): 7.61 (1H, d, *J* = 8.0 Hz), 7.29 (1H, d, *J* = 8.0 Hz), 7.21-7.19 (1H, m), 7.10-7.05 (1H, m), 7.03 (1H, br s), 6.09 (1H, s), 4.02 (1H, d, *J* = 13.5 Hz), 3.91 (2H, t, *J* = 7.0 Hz), 3.69 (1H, t, *J* = 7.5 Hz), 3.55 (1H, d, *J* = 13.5 Hz), 3.15 (1H, t, *J* = 7.5 Hz), 3.06 (1H, sept., *J* = 7.0 Hz), 2.39 (1H, q, *J* = 7.5 Hz), 2.21-2.11 (1H, m), 1.93-1.70 (5H, m), 1.85 (2H, sext., *J* = 7.5 Hz), 1.32 (6H, d, *J* = 7.0 Hz), 0.92 (3H, t, *J* = 7.50 Hz) ppm; **^{13}C NMR** (100 MHz, $CDCl_3$): δ 179.0 (C_q), 166.8 (C_q), 136.5 (C_q), 128.7 (C_q), 128.0 (CH), 121.5 (CH), 119.6 (CH), 119.1 (CH), 110.7 (C_q), 109.6 (CH), 97.4 (CH), 59.4 (CH), 53.4, (CH_2) 48.1 (CH_2), 47.6 (CH_2), 32.1 (CH_2), 27.5 (CH), 23.7 (CH_2), 22.8 (CH_2), 21.1 (CH_3), 11.8 (CH_3) ppm; **HRMS** (ESI): *m/z* $[M+H]^+$ calculated for $C_{22}H_{30}N_3O^+$ 352.2383, found 352.2387; **RP-HPLC**: t_R 16.3 mins (Method A).

1-Methyl-1*H*-indole-3-carbaldehyde 29

To a solution of formyl indole **14** (0.500 g, 3.44 mmol, 1 eq.) and potassium carbonate (0.522 g, 3.78 mmol, 1.1 eq.) in dry dimethylformamide (3.44 mL) under an atmosphere of nitrogen was added dimethylcarbonate (0.871 mL, 10.3 mmol, 3 eq.) and the reaction heated to 130 °C for 16 hours. Upon completion the reaction was cooled and diluted with water (35 mL) and extracted with ethyl acetate (2 × 35 mL). The combined organic phase was then washed with water (2 × 50 mL), brine (1 × 50 mL), dried with sodium sulfate and the solvent removed *in vacuo* to give indole **29** as a dark brown solid (0.505 g, 92%). **^{1}H NMR** (400 MHz, $CDCl_3$): δ 9.99 (1H, s), 8.32-8.29 (1H, m), 7.68 (1H, s), 7.37-7.35 (2H, m), 7.34-7.31 (1H, m), 3.87 (3H, s) ppm; **^{13}C NMR** (100 MHz, $CDCl_3$): δ 184.6 (CH), 139.4 (CH), 138.1 (C_q), 125.5 (C_q), 124.3 (CH), 123.2 (CH), 122.3 (CH), 118.4 (C_q), 110.1 (CH), 33.9 (CH_3) ppm; **HRMS** (ESI): *m/z* $[M+Na]^+$ calculated for $C_{10}H_9NNaO^+$ 182.0576, found 182.0574.

1-(Ethoxymethyl)-1*H*-indole-3-carbaldehyde 30

To a solution of formyl indole **14** (0.500 g, 3.44 mmol, 1 eq.) in dry tetrahydrofuran (12.4 mL) under an atmosphere of nitrogen at 0 °C was added sodium hydride 60% dispersion in mineral oil (0.179 g, 4.48 mmol, 1.3 eq.) and the reaction left to stir for 30 minutes. Chloromethyl ethyl ether (0.479 mL, 5.16 mmol, 1.5 eq.) was then added and the reaction stirred for a further hour at room temperature. Upon completion, water (5 mL) was added and the mixture extracted with dichloromethane (3 × 10 mL). The combined organic extracts were washed with brine (1 × 15 mL), dried with magnesium sulfate and the solvent removed *in vacuo*. The resultant solid was purified by flash column chromatography in 30% ethyl acetate:hexane resulting in indole **30** as a light pink solid (0.469 g, 67%). **^{1}H NMR** (400

MHz, $CDCl_3$): δ 10.01 (1H, s), 8.33-8.30 (1H, m), 7.76 (1H, s), 7.54-7.50 (1H, m), 7.37-7.30 (2H, m), 5.51 (2H, s), 3.46 (2H, q, J = 7.0 Hz), 1.16 (3H, t, J = 7.0 Hz) ppm; ^{13}**C NMR** (100 MHz, $CDCl_3$): δ 184.9 (CH), 138.6 (CH), 137.1 (C_q), 125.4 (C_q), 124.4 (CH), 123.2 (CH), 121.9 (CH), 118.7 (C_q), 110.8 (CH), 76.7 (CH_2), 64.4 (CH_2), 14.7 (CH_3) ppm; **HRMS** (ESI): *m/z* $[M+Na]^+$ calculated for $C_{12}H_{13}NNaO_2^+$ 226.0839, found 226.0839.

2-(3-Formyl-1*H*-indol-1-yl) acetonitrile 31

To a solution of formyl indole **14** (0.500 g, 3.44 mmol, 1 eq.) in dry tetrahydrofuran (5.16 mL) under an atmosphere of nitrogen at 0 °C was added sodium hydride 60% dispersion in mineral oil (0.151 g, 3.78 mmol, 1.1 eq.) and the reaction left to stir for 30 minutes. Bromoacetonitrile (0.480 mL, 6.88 mmol, 2 eq.) was then added and the reaction heated to reflux for 30 minutes followed by stirring at room temperature for 2 hours. Upon completion, water (4.5 mL) was added and the mixture extracted with dichloromethane (3 × 10 mL). The combined organic extracts were washed with brine (1 × 15 mL), dried with magnesium sulfate and the solvent removed *in vacuo*. The resultant solid was purified by flash column chromatography in 30% ethyl acetate:hexane to give indole **31** as a reddish-brown solid (0.578 g, 91%). ^{1}**H NMR** (400 MHz, $CDCl_3$): δ 10.05 (1H, s), 8.34 (1H, dd, J = 7.5, 1.0 Hz), 7.77 (1H, s), 7.47-7.38 (3H, m), 5.10 (2H, s) ppm; ^{13}**C NMR** (100 MHz, $CDCl_3$): δ 184.8 (CH), 137.2 (CH), 136.7 (C_q), 125.54 (C_q), 125.45 (CH), 124.2 (CH), 122.9 (CH), 120.3 (C_q), 113.4 (C_q), 109.5 (CH), 35.1 (CH_2) ppm; **HRMS** (ESI): *m/z* $[M+Na]^+$ calculated for $C_{11}H_8N_2NaO^+$ 207.0529, found 207.0531.

1-Benzyl-1*H*-indole-3-carbaldehyde 32

To a solution of formyl indole **14** (0.500 g, 3.44 mmol, 1 eq.) in dry tetrahydrofuran (17.5 mL) under an atmosphere of nitrogen at 0 °C was added sodium hydride 60% dispersion in mineral oil (0.151 g, 3.78 mmol, 1.1 eq.) and the reaction left to stir for 30 minutes. Benzyl bromide (0.647 g, 3.78 mmol, 1.1 eq.) was then added and the reaction stirred for a further hour at room temperature. Upon completion, water (4.5 mL) was added and the mixture extracted with dichloromethane (3 × 10 mL). The combined organic extracts were washed with brine (1 × 15 mL), dried with magnesium sulfate and the solvent removed *in vacuo*. The resultant solid was purified by flash column chromatography in 30% ethyl acetate:hexane to give indole **32** as a yellow-brown solid (0.587 g, 73%). ^{1}**H NMR** (400 MHz, $CDCl_3$): δ 9.97 (1H, s), 8.38-8.35 (1H, m), 7.66 (1H, s), 7.37-7.27 (5H, m), 7.18-7.16 (2H, m), 5.30 (1H, s) ppm; ^{13}**C NMR** (100 MHz, $CDCl_3$): δ 184.7 (CH), 138.8 (CH), 137.5 (C_q), 135.4 (C_q), 129.1 (CH), 128.4 (CH), 127.3 (CH), 125.5 (C_q), 124.2 (CH), 123.1 (CH), 122.2 (CH), 118.5 (C_q), 110.5 (CH), 50.9 (CH_2) ppm; **HRMS** (ESI): *m/z* $[M+Na]^+$ calculated for $C_{16}H_{13}NNaO^+$ 258.0889, found 258.0889.

(*S*)-5-Isopropyl-3-(1-((1-methyl-1*H*-indol-3-yl) methyl) pyrrolidin-2-yl) isoxazole 33

General procedure 4 using isoxazole **2** (0.200 g, 1.11 mmol, 1 eq.) and aldehyde **29** gave product **33** as a brown oil (0.178 g, 50%). **^{1}H NMR** (400 MHz, $CDCl_3$): 7.63 (1H, d, *J* = 8.0 Hz), 7.29 (1H, d, *J* = 8.0 Hz), 7.25-7.21 (1H, m), 7.14-7.10 (1H, m), 7.04 (1H, s), 6.16 (1H, m), 4.07 (1H, d, *J* = 13.5 Hz), 3.82-3.74 (1H, m), 3.75 (3H, s), 3.62 (1H, d, *J* = 13.5 Hz), 3.24-3.20 (1H, m), 3.08 (1H, sept., J = 7.0 Hz), 2.46 (1H, q, J = 8.5 Hz), 2.23-2.15 (1H, m), 2.00-1.88 (2H, m), 1.84-1.74 (1H, m), 1.34 (6H, d, J = 7.0 Hz) ppm; **^{13}C NMR** (100 MHz, $CDCl_3$): δ 179.0 (C_q), 166.2 (C_q), 137.0 (C_q), 129.1 (CH), 128.5 (C_q), 121.7 (CH), 119.4 (CH), 119.2 (CH), 109.9 (C_q), 109.3 (CH), 97.4 (CH), 59.3 (CH), 53.2 (CH_2), 47.2 (CH_2), 32.8 (CH_3), 31.8 (CH_2), 27.4 (CH), 22.6 (CH_2), 21.0 (CH_3) ppm; **HRMS** (ESI): *m/z* $[M+H]^+$ calculated for $C_{20}H_{26}N_3O^+$ 324.2070, found 324.2077; **RP-HPLC**: t_R 14.7 mins (Method A).

(*S*)-3-(1-((1-(Ethoxymethyl)-1*H*-indol-3-yl) methyl) pyrrolidin-2-yl)-5-isopropylisoxazole 34

General procedure 4 using isoxazole **2** (0.200 g, 1.11 mmol, 1 eq.) and aldehyde **30** gave product **34** as a brown oil (0.138 g, 34%). **^{1}H NMR** (400 MHz, $CDCl_3$): 7.62 (1H, d, *J* = 8.0 Hz), 7.46 (1H, d, *J* = 8.0 Hz), 7.25-7.20 (1H, m), 7.16-7.11 (1H, m), 7.13 (1H, m), 6.11 (1H, s), 5.46 (2H, s), 4.03 (1H, d, *J* = 13.5 Hz), 3.64 (1H, t, *J* = 8.0 Hz), 3.58 (1H, d, *J* = 13.5 Hz), 3.41 (2H, q, *J* = 7.0 Hz), 3.22-3.15 (1H, m), 3.06 (1H, sept., *J* = 7.0 Hz), 2.41 (1H, q, *J* = 8.0 Hz), 2.24-2.14 (1H, m), 1.98-1.85 (2H, m), 1.83-1.74 (1H, m), 1.31 (6H, d, *J* = 7.0 Hz), 1.14 (3H, t, *J* = 7.0 Hz) ppm; **^{13}C NMR** (100 MHz, $CDCl_3$): δ 178.9 (C_q), 166.5 (C_q), 136.8 (C_q), 129.2 (C_q), 127.9 (CH), 122.3 (CH), 120.0 (CH), 119.6 (CH), 112.1 (C_q), 110.0 (CH), 97.3 (CH), 76.0 (CH_2), 63.9 (CH_2), 59.5 (CH), 53.4 (CH_2), 47.6 (CH_2), 32.0 (CH_2), 27.3 (CH), 22.7 (CH_2), 21.0 (CH_3), 15.0 (CH_3) ppm; **HRMS** (ESI): *m/z* $[M+H]^+$ calculated for $C_{22}H_{30}N_3O_2^+$ 368.2333, found 368.2340; **RP-HPLC**: t_R 15.2 mins (Method A).

(*S*)-2-(3-((2-(5-Isopropylisoxazol-3-yl) pyrrolidin-1-yl) methyl)-1*H*-indol-1-yl) acetonitrile 35

General procedure 4 using isoxazole **2** (0.180 g, 0.98 mmol, 1 eq.) and aldehyde **31** gave product **35** as a brown oil (0.129 g, 38%). **^{1}H NMR** (400 MHz, $CDCl_3$): 7.64 (1H, d, *J* = 8.0 Hz), 7.33-7.25 (2H, m), 7.20-7.15 (1H, m), 7.01 (1H, s), 6.08 (1H, s), 4.91 (2H, s), 3.99 (1H, d, *J* = 13.5 Hz), 3.70 (1H, t, *J* = 8.0 Hz), 3.54 (1H, d, *J* = 13.5 Hz), 3.18-3.11 (1H, m), 3.05 (1H, sept., *J* = 7.0 Hz), 2.37 (1H, q, *J* = 8.0 Hz), 2.28-2.13 (1H, m), 1.96-1.84 (2H, m), 1.83-1.73 (1H, m), 1.31 (6H, d, *J* = 7.0 Hz) ppm; **^{13}C NMR** (100 MHz, $CDCl_3$): δ 178.8 (C_q), 166.6 (C_q), 136.1 (C_q), 129.0 (C_q), 126.7 (CH), 122.9 (CH), 120.5 (CH), 120.1 (CH), 114.6 (C_q), 114.1 (C_q), 108.9 (CH), 97.2 (CH), 59.6 (CH), 53.5 (CH_2), 47.6 (CH_2), 34.1 (CH_2), 31.9 (CH_2), 27.2 (CH), 22.6 (CH_2), 20.9 (CH_3) ppm; **HRMS** (ESI): *m/z* $[M+H]^+$ calculated for $C_{21}H_{25}N_4O^+$ 349.2023, found 349.2025; **RP-HPLC**: t_R 14.0 mins (Method A).

(*S*)-3-(1-((1-Benzyl-1*H*-indol-3-yl) methyl) pyrrolidin-2-yl)-5-isopropylisoxazole 36

General procedure 4 using isoxazole **2** (0.200 g, 1.11 mmol, 1 eq.) and aldehyde **32** gave product **36** as a brown oil (0.121 g, 27 %). **^{1}H NMR** (400 MHz, $CDCl_3$): 7.68 (1H, d, *J* = 7.5 Hz), 7.34-7.24 (4H, m), 7.22-7.09 (5H, m), 6.20 (1H, s), 5.31 (2H, s), 4.12 (1H, d, *J* = 13.5 Hz), 3.82 (1H, t, *J* = 8.0 Hz), 3.66

(1H, d, J = 13.5 Hz), 3.26 (1H, t, J = 8.0 Hz), 3.09 (1H, sept., J = 7.0 HZ), 2.50 (1H, q, J = 8.0 Hz), 2.27-2.18 (1H, m), 2.04-1.91 (2H, m), 1.87-1.77 (1H, m), 1.34 (6H, d, J = 7.0 Hz) ppm; **^{13}C NMR** (100 MHz, $CDCl_3$): δ 179.0 (C_q), 166.1 (C_q), 137.6 (C_q), 136.7 (C_q), 128.82 (CH), 128.75 (C_q), 128.5 (CH), 127.7 (CH), 126.8 (CH), 121.9 (CH), 119.6 (CH), 119.5 (CH), 110.8 (C_q), 109.8 (CH), 97.5 (CH), 59.5 (CH), 53.3 (CH_2), 50.1 (CH_2), 47.5 (CH_2), 31.9 (CH_2), 27.4 (CH), 22.6, (CH_2) 21.0 (CH_3) ppm; **HRMS** (ESI): *m/z* $[M+H]^+$ calculated for $C_{26}H_{30}N_3O^+$ 400.2383, found 400.2387; **RP-HPLC**: t_R 17.0 mins (Method A).

Methyl 1-ethyl-1*H*-indazole-3-carboxylate 38

To a solution of methyl 1*H*-indazole-3-carboxylate **37** (1.00 g, 5.68 mmol, 1 eq.) in tetrahydrofuran (28.0 mL) at 0 °C was added potassium *tert*-butoxide (0.700 g, 6.24 mmol, 1.1 eq). The mixture was then allowed to warm to room temperature and stir for 1 hour. It was then cooled back down to 0 °C and bromoethane (0.678 mL, 9.08 mmol, 1.6 eq.) added dropwise, before being allowed warmed to room temperature and stirred for 24 hours. Water (20 mL) was added, and the reaction extracted with ethyl acetate (2 × 20 mL). The combined organic extracts were then washed with water (1 × 20 mL), brine (1 × 20 mL), dried with magnesium sulfate and the solvent removed *in vacuo* giving the alkylated indazole **38** as a white solid (0.338 g, 29%). **^{1}H NMR** (400 MHz, $CDCl_3$): δ 8.22 (1H, dt, J = 8.0, 1.0 Hz), 7.47-7.40 (1H, m), 7.32-7.28 (1H, m), 4.52 (2H, q, J = 7.5 Hz), 4.02 (3H, s), 1.54 (3H, t, J = 7.5 Hz) ppm; **^{13}C NMR** (100 MHz, $CDCl_3$): δ 163.2 (C_q), 140.2 (C_q), 134.6 (C_q), 126.9 (CH), 124.0 (C_q), 123.2 (CH), 122.4 (CH), 109.7 (CH), 52.1 (CH_3), 45.0 (CH_2), 15.1 (CH_3) ppm; **HRMS** (ESI): *m/z* $[M+Na]^+$ calculated for $C_{11}H_{12}N_2NaO_2^+$ 227.0791, found 227.0791.

(1-Ethyl-1*H*-indazol-3-yl) methanol 39

To a solution of indazole **38** (0.338 g, 1.6 mmol, 1 eq.) in dry dichloromethane (11.0 mL) under an atmosphere of nitrogen at -78 °C was added a 1M solution of DIBAL in toluene (1.82 mL, 1.82 mmol, 1.1 eq.). The mixture was then maintained at this temperature and left to stir for 2 hours. The reaction was quenched by slow addition of methanol (5 mL), then water (10 mL) and allowed to warm to room temperature. The organic phase was then separated, washed with brine (1 × 10 mL), dried with sodium sulfate and the solvent removed resulting in alcohol **39** as a white solid (0.170 g, 58%). **^{1}H NMR** (400 MHz, $CDCl_3$): δ 7.80 (1H, dt, J = 8.0, 1.0 Hz), 7.35-7.27 (1H, m), 7.12-7.08 (1H, m), 4.98 (2H, s), 4.23 (2H, q, J = 7.5 Hz), 1.34 (3H, t, J = 7.5 Hz) ppm; **^{13}C NMR** (100 MHz, $CDCl_3$): δ 144.3 (C_q), 140.0 (C_q), 126.5 (CH), 122.2 (C_q), 120.8 (CH), 120.4 (CH), 108.9 (CH), 57.6 (CH_2), 43.4 (CH_2), 14.8 (CH_3) ppm; **HRMS** (ESI): *m/z* $[M+Na]^+$ calculated for $C_{10}H_{12}N_2NaO^+$ 199.0842, found 199.0849.

1-Ethyl-1*H*-indazole-3-carbaldehyde 40

To a solution of alcohol **39** (0.170 g, 0.965 mmol, 1 eq.) in dichloromethane (10 mL) was added manganese dioxide (1.68 g, 19.3 mmol, 20 eq.) and the reaction left to stir for 2 hours. The reaction was

filtered through Celite®, followed by removal of the solvent *in vacuo* to give aldehyde **40** as a white solid (0.133 g, 79%). **^{1}H NMR** (400 MHz, $CDCl_3$): δ 10.21 (1H, s), 8.28 (1H, dt, *J* = 8.0, 1.0 Hz), 7.48-7.42 (2H, m), 7.35-7.30 (1H, m), 4.50 (2H, q, *J* = 7.5 Hz), 1.58 (3H, t, *J* = 7.5 Hz) ppm; **^{13}C NMR** (100 MHz, $CDCl_3$): δ 186.9 (CH), 143.0 (C_q), 140.6 (C_q), 127.4 (CH), 124.1 (CH), 122.4 (CH), 122.3 (C_q), 109.6 (CH), 45.0 (CH_2), 14.8 (CH_3) ppm; **HRMS** (ESI): *m/z* $[M+H]^+$ calculated for $C_{10}H_{10}N_2NaO^+$ 197.0685, found 197.0684.

(*S*)-3-(1-((1-Ethyl-1*H*-indazol-3-yl) methyl) pyrrolidin-2-yl)-5-isopropylisoxazole 41

General procedure 4 using isoxazole **2** (0.138 g, 0.764 mmol, 1 eq.) and aldehyde **40** gave product **41** as a brown oil (0.041 g, 16%). **^{1}H NMR** (400 MHz, $CDCl_3$): 7.72 (1H, dd, *J* = 8.0, 1.0 Hz), 7.35-7.31 (2H, m), 7.10-7.06 (1H, m), 6.07 (1H, s), 4.38 (2H, q, *J* = 7.5 Hz), 4.16 (1H, d, *J* = 13.5 Hz), 3.77 (1H, t, *J* = 8.0 Hz), 3.74 (1H, d, *J* = 13.5 Hz), 3.12 (1H, t, *J* = 8.0 Hz), 3.04 (1H, sept., *J* = 7.0 Hz), 2.48 (1H, q, *J* = 8.0 Hz), 2.26-2.15 (1H, m), 1.95-1.76 (3H, m), 1.45 (3H, t, *J* = 7.0 Hz), 1.30 (6H, d, *J* = 7.0 Hz) ppm; **^{13}C NMR** (100 MHz, $CDCl_3$): δ 178.8 (C_q), 166.8 (C_q), 142.2 (C_q), 140.0 (C_q), 126.2 (CH), 123.4 (C_q), 121.2 (CH), 120.0 (CH), 109.0 (CH), 97.3 (CH), 60.5 (CH), 54.0 (CH_2), 50.0 (CH_2), 43.7 (CH_2), 32.2 (CH_2), 27.4 (CH), 23.0 (CH_2), 21.1 (CH_3), 15.2 (CH_3) ppm; **HRMS** (ESI): *m/z* $[M+H]^+$ calculated for $C_{20}H_{27}N_4O^+$ 339.2179, found 339.2184; **RP-HPLC**: t_R 14.0 mins (Method A).

Methyl 1-ethyl-1*H*-indole-3-carboxylate 42

General procedure 3 using methyl 1*H*-indole-3-carboxylate **11** (1.00 g, 5.71 mmol) gave alkyl indole **42** as a clear oil (1.14 g, 98%). **^{1}H NMR** (400 MHz, $CDCl_3$): δ 8.22-8.19 (1H, m), 7.85 (1H, s), 7.39-7.35 (1H, m), 7.31-7.27 (2H, m), 4.17 (2H, q, *J* = 7.5 Hz), 3.92 (3H, s), 1.50 (3H, t, *J* = 7.5 Hz) ppm; **^{13}C NMR** (100 MHz, $CDCl_3$): δ 165.7 (C_q), 136.4 (C_q), 133.6 (CH), 126.9 (C_q), 122.8 (CH), 122.0 (CH), 121.9 (CH), 110.0 (CH), 107.1 (C_q), 51.1 (CH_3), 41.7 (CH_2), 15.3 (CH_3) ppm; **HRMS** (ESI): *m/z* $[M+Na]^+$ calculated for $C_{12}H_{13}NNaO_2^+$ 226.0839, found 226.0840.

1-Ethyl-1*H*-indole-3-carboxylic acid 43

To a solution of ethyl indole methyl ester **42** (0.500 g, 2.46 mmol, 1 eq.) in tetrahydrofuran (20 mL) was added 0.25M KOH (19.7 mL, 4.92 mmol, 2 eq.) and the reaction heated to reflux for 16 hours. Upon completion the reaction was cooled to room temperature and neutralised with 1M HCl. The mixture was then extracted with ethyl acetate (3 × 25 mL) and the combined organic extracts were washing with brine (1 × 50 mL), dried with magnesium sulfate and the solvent removed *in vacuo* to give carboxylic acid **43** as a pink solid (0.297 g, 64%). **^{1}H NMR** (400 MHz, d_6-DMSO): δ 11.96 (1H, br s), 8.08 (1H, s), 8.03-8.01 (1H, m), 7.56 (1H, d, *J* = 8.0 Hz), 7.25-7.17 (2H, m), 4.26 (2H, q, *J* = 7.0 Hz), 1.38 (3H, t *J* = 7.0 Hz), ppm; **^{13}C NMR** (100 MHz, d_6-DMSO): δ 165.6 (C_q), 136.0 (C_q), 134.6 (CH), 126.6 (C_q), 122.2 (CH), 121.2 (CH), 120.9 (CH), 110.6 (CH), 106.4 (C_q), 40.9 (CH_2), 15.2 (CH_3) ppm; **HRMS** (ESI): *m/z* $[M+Na]^+$ calculated for $C_{11}H_{11}NNaO_2^+$ 212.0682 , found 212.0682.

(*S*)-(1-Ethyl-1*H*-indol-3-yl) (2-(5-isopropylisoxazol-3-yl) pyrrolidin-1-yl) methanone 44

To a solution of carboxylic acid **43** (0.100 g, 0.53 mmol, 1 eq.) in dry dichloromethane (1.06 mL) under an atmosphere of nitrogen at 0 °C was added dimethylformamide (0.002 mL, 0.027 mmol, 0.05 eq.) followed by oxalyl chloride (0.067 mL, 0.795 mmol, 1.5 eq.) and the reaction left to stir for 16 hours. The solvent was then removed *in vacuo* and the remaining oil dissolved in fresh dichloromethane (5.3 mL) followed by addition of triethylamine (0.148 mL, 1.06 mmol, 2 eq.) and isoxazole **2** (0.096 g, 0.53 mmol, 1 eq.). The reaction was then left to stir for 16 hours. Upon completion the solvent was removed *in vacuo* and the remaining slurry dissolved into ethyl acetate (20 mL). This solution was washed with 1M HCl (2 × 10 mL), saturated sodium bicarbonate (3 × 10 mL), brine (1 × 10 mL), dried with magnesium sulfate and the solvent removed *in vacuo* to give product **44** as a white solid (0.181 g, 97%). **^{1}H NMR** (400 MHz, $CDCl_3$): δ 8.14 (1H, d, J = 8.0 Hz), 7.45 (1H, br s), 7.33 (1H, d, J = 8.0 Hz), 7.27-7.25 (1H, m), 7.23-7.18 (1H, m), 6.00 (1H, s), 5.54 (1H, br s), 4.20-4.11 (2H, m), 3.90-3.82 (2H, m), 3.00 (1H, sept., J = 7.0 Hz), 2.36-2.24 (2H, m), 2.18-2.06 (1H, m), 2.05-1.95 (1H, m), 1.47-1.41 (3H, m), 1.25 (6H, d, J = 7.0 Hz) ppm; **^{13}C NMR** (100 MHz, $CDCl_3$): δ 178.8 (C_q), 166.0 (C_q), 165.8 (C_q), 135.7 (C_q), 129.5 (CH), 127.9 (C_q), 122.7 (CH), 122.3 (CH), 121.3 (CH), 110.6 (C_q), 109.6 (CH), 98.7 (CH), 54.2 (CH), 49.4 (CH_2), 41.5 (CH_2), 30.5 (CH_2), 27.3 (CH), 25.4 (CH_2), 21.0 (CH_3), 15.4 (CH_3); **HRMS** (ESI): *m/z* $[M+H]^+$ calculated for $C_{21}H_{26}N_3O_2^+$ 352.2020, found 352.2024; **RP-HPLC**: t_R 17.8 mins (Method A).

(*tert*-Butoxycarbonyl)-*D*-proline 46

To a solution of D-proline **45** (5.00 g, 43.4 mmol, 1 eq.) in dry dichloromethane (100 mL) under an atmosphere of nitrogen was added di-*tert*-butyl dicarbonate (10.4 g, 47.8 mmol, 1.1 eq.) and dry triethylamine (6.66 mL, 47.8 mmol, 1.1 eq.) and the reaction left to stir for 16 hours. Upon completion the reaction was neutralised with 1M hydrochloric acid, the organic phase separated and washed with brine (1 × 50 mL), dried with magnesium sulfate and the solvent removed *in vacuo* to give the Boc protected D-proline **7.03** as a white solid (8.51 g, 91%). **^{1}H NMR** (400 MHz, $CDCl_3$, exists as 1:1 mixture of isomers): δ 10.71 (2H, br. s), 4.34-4.33 (1H, m), 4.23-4.20 (1H, m), 3.54-3.34 (4H, m), 2.28-1.84 (8H, m), 1.46 (9H, s), 1.40 (9H, s) ppm; **^{13}C-NMR** (100 MHz, $CDCl_3$, exists as a 1:1 mixture of isomers): δ 178.0 (C_q), 175.0 (C_q), 156.1 (C_q), 154.2 (C_q), 81.0 (C_q), 80.2 (C_q), 59.4 (2 × CH), 47.1 (CH_2), 46.5 (CH_2), 31.1 (CH_2), 29.1 (CH_2), 28.6 (CH_3), 28.5 (CH_3), 24.5 (CH_2), 23.8 (CH_2) ppm; **HRMS** (ESI): *m/z* $[M+Na]^+$ calculated for $C_{10}H_{17}NNaO_4^+$ 238.1050, found 238.1054; **Optical Rotation** $[\alpha]^{26}_{D}$: +55° (c 1, acetic acid).

***tert*-Butyl (*R*)-2-(hydroxymethyl) pyrrolidine-1-carboxylate 47**

To a solution of Boc protected proline **46** (8.00 g, 37.2 mmol, 1 eq.) in tetrahydrofuran (64 mL) and cooled to 0 °C was added sodium borohydride (2.25 g, 59.5 mmol, 1.6 eq.) over a 30-minute period

followed by boron trifluoride diethyl etherate (9.17 mL, 74.3 mmol, 2 eq.) and the vessel warmed to room temperature and allowed to stir for 16 hours. Upon completion the reaction was diluted with saturated sodium hydroxide (50 mL) and the solvent removed *in vacuo*. The remaining aqueous phase was then extracted with ethyl acetate (4 × 25 mL). The combined organic phase was then washed with saturated potassium carbonate (2 × 25 mL), brine (1 × 25 mL), dried with sodium sulfate and the solvent removed *in vacuo* resulting in the alcohol **47** as a white solid (4.49 g, 60%). **^{1}H NMR** (400 MHz, $CDCl_3$, exists as a 0.2:1 mixture of isomers): δ 4.73 (2H, s, maj. + min.), 3.98-3.87 (1H, m, maj.), 3.86-3.76 (1H, m, min.), 3.62-3.50 (4H, m, maj. + min.), 3.45-3.36 (2H, m, maj. + min.), 3.31-3.22 (2H, m, maj. + min.), 2.03-1.90 (2H, m, maj. + min.), 1.89-1.66 (4H, m, maj. + min.) 1.61-1.47 (1H, m, maj.), 1.43 (19H, s, maj. + min.) ppm; **^{13}C-NMR** (100 MHz, $CDCl_3$): δ 157.2 (C_q), 80.3 (C_q), 67.7 (CH_2), 60.3 (CH), 47.7 (CH_2), 28.8 (CH_2), 28.6 (CH_3), 24.2 (CH_2) ppm; **HRMS** (ESI): *m/z* $[M+Na]^+$ calculated for $C_{10}H_{19}NNaO_3^+$ 224.1257, found 224.1259; **Optical Rotation** $[\alpha]^{25}_{D}$: +52° (c 1, methanol).

***tert*-Butyl (*R*)-2-formylpyrrolidine-1-carboxylate 48**

To a solution of Boc protected prolinol **47** (4.00 g, 19.9 mmol, 1 eq.) in dichloromethane (43 mL) under an atmosphere of nitrogen at 0 °C was added triethylamine (8.73 mL, 62.6 mmol, 3.15 eq.) and dimethylsulfoxide (7.06 mL, 99.4 mmol, 5 eq.). Pyridine sulfur trioxide complex (9.49 g, 59.6 mmol, 3 eq.) was then added portion wise and the reaction left to stir for 1 hour before being warmed to room temperature and stirred for another hour. Upon completion the reaction was diluted with brine (25 mL) and the solvent removed *in vacuo*. The aqueous phase was then extracted with ethyl acetate (3 × 25 mL). The combined organic phase was then washed with brine (1 × 50 mL), dried with sodium sulfate and the solvent removed *in vacuo* to give a yellow-brown oil. The yellow oil was then dissolved into a 50:50 mixture of ethyl acetate and hexane and passed through a plug of silica, followed again by solvent removal *in vacuo* producing the aldehyde **48** as a light-yellow oil (2.65 g, 85%). **^{1}H NMR** (400 MHz, $CDCl_3$, exists as 0.6:1 a mixture of isomers): δ 9.50 (1H, s, min), 9.41 (1H, s maj.), 4.16-4.13 (1H, m, min.), 4.02-3.98 (1H, m, maj.), 3.55-3.38 (4H, m, maj. + min.), 2.12-1.80 (8H, m, maj. + min.), 1.43 (9H, s, min.), 1.38 (9H, s, maj.) ppm; **^{13}C NMR** (100 MHz, $CDCl_3$, exists as a 0.6:1 mixture of isomers): δ 200.8 (CH, min.), 200.5 (CH, maj.), 155.0 (C_q, min.), 154.1 (C_q, maj.), 80.7 (C_q, maj.), 80.3 (C_q, min.), 65.1 (CH, maj.), 65.0 (CH, min.), 47.0 (CH_2, min.), 46.8 (CH_2, maj.), 28.5 (CH_3, min.), 28.4 (CH_3, maj.), 28.1 (CH_2, maj.), 26.8 (CH_2, min.), 24.7 (CH_2, min.), 24.1 (CH_2, maj.) ppm; **HRMS** (ESI): *m/z* $[M+Na]^+$ calculated for $C_{10}H_{17}NNaO_3^+$ 222.1106, found 222.1111; **Optical Rotation** $[\alpha]^{22}_{D}$: +100° (c 1, chloroform).

***tert*-Butyl (*R*)-2-((hydroxyimino)methyl) pyrrolidine-1-carboxylate 49**

To a solution of Boc protected prolinal **48** (2.00 g, 10.0 mmol, 1 eq.) in methanol (10 mL) at 0 °C was added hydroxylamine hydrochloride (0.767 g, 11.0 mmol, 1.1 eq.) and sodium bicarbonate (1.10 g, 13.0 mmol, 1.3 eq.). The reaction was then allowed to warm to room temperature and stirred for 16 hours.

Upon completion the solvent was removed *in vacuo* and the resultant slurry dissolved into brine (10 mL) and extracted with ethyl acetate (4 × 20 mL). The combined organic phase was then passed through a silica plug, dried with sodium sulfate and the solvent removed *in vacuo* resulting in oxime **49** as a clear oil (1.74 g, 81%). **^{1}H NMR** (400 MHz, MeOD, exists as a 0.5:1 mixture of isomers): δ 7.27 (1H, br s, maj.), 6.64 (1H, br s, min.), 4.90-4.81 (1H, m, min.), 4.40-4.27 (1H, m, maj.), 3.45-3.36 (4H, m, maj. + min.), 2.31-2.20 (1H, m, min.), 2.17-2.02 (1H, m, maj.), 2.00-1.79 (6H, m, maj. + min.), 1.47 (9H, s, min.), 1.44 (9H, s, maj.) ppm; **^{13}C NMR** (100 MHz, MeOD, exists as a 0.5:1 mixture of isomers): δ 156.2 (C_q, maj. + min.), 154.5 (CH, min.), 151.5 (CH, maj.), 81.1 (C_q, maj. + min.), 57.5 (CH, maj.), 54.1 (CH, min.), 47.6 (CH_2, maj. + min.), 31.3 (CH_2, maj. + min.), 24.5 (CH_2, maj. + min.) ppm; **HRMS** (ESI): *m/z* $[M+Na]^+$ calculated for $C_{10}H_{18}N_2NaO_3^+$ 237.1210, found 237.1209.

***tert*-Butyl (*R*)-2-(chloro(hydroxyimino)methyl) pyrrolidine-1-carboxylate 50**

To a solution of oxime **49** (1.50 g, 7.00 mmol, 1 eq.) in dimethylformamide (10 mL) at 0 °C was added 4M HCl in dioxane (0.175 mL, 0.70 mmol, 0.1 eq.) followed by *N*-chlorosuccinimide (1.00 g, 7.49 mmol, 1.1 eq.) whilst maintaining the temperature at 0 °C. The reaction was then stirred at room temperature for 16 hours. Upon completion the reaction was diluted with water (25 mL) and extracted with ethyl acetate (4 × 10 mL). The combined organic extracts were then washed with water (1 × 20 mL), brine (1 × 20 mL), dried with sodium sulfate and the solvent removed *in vacuo* to give chlorooxime **50** as a light-yellow solid (1.41 g, 84%). **^{1}H NMR** (400 MHz, $CDCl_3$, exists as a 0.5:1 mixture of isomers): δ 9.54 (1H, br s, maj.), 9.41 (1H, br s, min.), 4.72-4.61 (1H, m, min.), 4.56-4.46 (1H, m, maj.), 3.54-3.40 (4H, m, maj. + min.), 2.20-2.09 (2H, m, maj. + min.), 2.08-1.90 (4H, m, maj. + min.), 1.90-1.76 (2H, m, maj. + min.), 1.44 (9H, s, min.), 1.39 (9H, s, maj.) ppm; **^{13}C NMR** (100 MHz, $CDCl_3$, exists as a 0.5:1 mixture of isomers): δ 154.59 (C_q, min.), 154.54 (C_q, maj.), 141.1 (C_q, maj.), 140.6 (C_q, min.), 80.7 (C_q, maj.), 80.6 (C_q, min.), 61.2 (CH, maj.), 60.7 (CH, min.), 47.3 (CH_2, min.), 46.6 (CH_2, maj.), 31.0 (CH_2, maj.), 30.4 (CH_2, min.), 28.6 (CH_3, min.), 28.5 (CH_3, maj.), 23.9 (CH_2, min.), 23.0 (CH_2, maj.) ppm.

(*R*)-5-Isopropyl-3-(pyrrolidin-2-yl) isoxazole hydrochloride 51

General procedure 1 using chlorooxime **50** (0.500 g, 2.01 mmol) and 3-methyl-1-butyne gave isoxazole **51** as a brown oil (0.152 g, 42% over 2 steps). **^{1}H NMR** (400 MHz, $CDCl_3$): δ 5.90 (1H, s), 4.26-4.22 (1H, m), 3.61 (2H, br s), 3.10-3.05 (1H, m), 3.01-2.94 (2H, m), 2.16-2.11 (1H, m), 1.89-1.77 (3H, m), 1.24 (6H, d, J = 7.0 Hz) ppm; **^{13}C NMR** (100 MHz, $CDCl_3$): δ 179.0 (C_q), 166.5 (C_q), 93.3 (CH), 54.6 (CH), 46.7 (CH_2), 31.8 (CH_2), 27.3 (CH), 25.4 (CH_2), 20.9 (CH_3) ppm; **HRMS** (ESI): *m/z* $[M+H]^+$ calculated for $C_{10}H_{17}N_2O^+$ 181.1335, found 181.1339; **RP-HPLC**: t_R 10.6 mins (Method A); **Optical Rotation** $[\alpha]^{24}_D$ = +53° (c 0.5, chloroform).

(*R*)-3-(1-((1-Ethyl-1*H*-indol-3-yl) methyl) pyrrolidin-2-yl)-5-isopropylisoxazole 52

General procedure 4 using isoxazole **51** (0.152 g, 1 mmol) and indole **15** gave product **52** as an orange-brown oil (0.179 g, 53%). **^{1}H NMR** (400 MHz, $CDCl_3$): δ 7.65 (1H, d, J = 8.0 Hz), 7.32 (1H, d, J = 8.0 Hz), 7.21 (1H, t, J = 8.0 Hz), 7.11 (1H, t, J = 8.0 Hz), 7.06 (1H, s), 6.10 (1H, s), 4.14 (2H, q, J = 7.5 Hz), 4.04 (1H, d, J = 13.5 Hz), 3.71 (1H, t, J = 8.0 Hz), 3.58 (1H, d, J = 13.5 Hz), 3.21-3.15 (1H, m), 3.08 (1H, sept., J = 8.0 Hz), 2.41 (1H, q, J = 8.0 Hz), 2.24-2.15 (1H, m), 1.98-1.85 (2H, m), 1.85-1.74 (1H, m), 1.46 (3H, t, J = 8.0 Hz), 1.34 (6H, d, J = 7.0 Hz) ppm; **^{13}C NMR** (100 MHz, $CDCl_3$): δ 178.7 (C_q), 167.1 (C_q), 136.1 (C_q), 128.7 (C_q), 126.9 (CH), 121.4 (CH), 119.6 (CH), 118.9 (CH), 111.1 (C_q), 109.3 (CH), 97.2, (CH) 59.4 (CH), 53.6 (CH_2), 47.8 (CH_2), 40.9 (CH_2), 32.2 (CH_2), 27.3 (CH), 22.8 (CH_2), 21.0 (CH_3), 15.6 (CH_3) ppm; **HRMS** (ESI): *m/z* $[M+H]^+$ calculated for $C_{21}H_{28}N_3O^+$ 338.2227, found 338.2233; **RP-HPLC**: t_R 15.3 mins (Method A); **Chiral NP-HPLC**: t_R 7.5 mins; **Optical Rotation** $[\alpha]^{22}_D$ = +94° (c 1, chloroform).

1-(*tert*-Butoxycarbonyl) piperidine-2-carboxylic acid 54

To a solution of pipecolinic acid **53** (5.00 g, 38.7 mmol, 1 eq.) in dry dichloromethane (89 mL) under an atmosphere of nitrogen was added di-*tert*-butyl dicarbonate (9.29 g, 42.6 mmol, 1.1 eq.) and dry triethylamine (5.94 mL, 42.6 mmol, 1.1 eq.) and the reaction left to stir for 16 hours. Upon completion the reaction was neutralised with 1M hydrochloric acid, the organic phase separated and washed with brine (1 × 45 mL), dried with magnesium sulfate and the solvent removed *in vacuo* giving the Boc protected product **54** as a white solid (8.34 g, 94%). **^{1}H NMR** (400 MHz, $CDCl_3$): δ 9.40 (1H, br s), 4.92-4.64 (1H, m), 4.02-3.82 (1H, m), 3.03-2.81 (1H, m), 2.26-2.12 (1H, m), 1.70-1.54 (3H, m), 1.46-1.26 (2H, m), 1.41 (9H, s) ppm; **^{13}C NMR** (100 MHz, $CDCl_3$): δ 177.6 (C_q), 156.4 (C_q), 80.4 (C_q), 54.4 (CH), 41.8 (CH_2), 28.5 (CH_3), 26.9 (CH_2), 25.0 (CH_2), 21.0 (CH_2) ppm; **HRMS** (ESI): *m/z* $[M+Na]^+$ calculated for $C_{11}H_{19}NNaO_4^+$ 252.1206, found 252.1208.

***tert*-Butyl 2-(hydroxymethyl) piperidine-1-carboxylate 55**

To a solution of acid **54** (8.00 g, 34.9 mmol, 1 eq.) in tetrahydrofuran (60 mL) and cooled to 0 °C was added sodium borohydride (2.11 g, 55.8 mmol, 1.6 eq.) over a 30-minute period followed by boron trifluoride diethyl etherate (8.61 mL, 69.8 mmol, 2 eq.). The reaction was allowed to warm to room temperature and stirred for 16 hours. Upon completion the reaction was diluted with saturated sodium hydroxide (50 mL) and the solvent removed *in vacuo*. The remaining aqueous phase was then extracted with ethyl acetate (4 × 20 mL). The combined organic phase was then washed with saturated potassium carbonate (2 × 20 mL), brine (1 × 20 mL), dried with sodium sulfate and the solvent removed *in vacuo* to produce the alcohol **55** as a white solid (6.91 g, 60%). **^{1}H NMR** (400 MHz, $CDCl_3$): δ 4.30-4.23 (1H, m), 3.91 (1H, d, J = 13.5 Hz), 3.77 (1H, dd, J = 11.0, 9.0 Hz), 3.59 (1H, dd, J = 11.0, 6.0 Hz), 2.84 (1H, t, J = 12.0 Hz), 1.71-1.63 (1H, m), 1.63-1.53 (3H, m), 1.50-1.36 (2H, m), 1.44 (9H, s) ppm; **^{13}C NMR** (100 MHz, $CDCl_3$): δ 156.4 (C_q), 80.0 (C_q), 61.8 (CH_2), 52.6 (CH), 40.1 (CH_2), 28.6 (CH_3), 25.41 (CH_2),

25.38 (CH_2), 19.8 (CH_2) ppm; **HRMS** (ESI): *m/z* $[M+Na]^+$ calculated for $C_{11}H_{21}NNaO_3^+$ 238.1414, found 238.1411.

***tert*-Butyl 2-formylpiperidine-1-carboxylate 56**

To a solution of alcohol **55** (6.50 g, 30.2 mmol, 1 eq.) in dichloromethane (65 mL) under an atmosphere of nitrogen at 0 °C was added triethylamine (13.3 mL, 95.1 mmol, 3.15 eq.) and dimethylsulfoxide (10.7 mL, 151.0 mmol, 5 eq.). Pyridine sulfur trioxide complex (14.4 g, 90.6 mmol, 3 eq.) was then added portion wise and the reaction left to stir for 1 hour before being warmed to room temperature and stirred for another hour. Upon completion the reaction was diluted with brine (40 mL) and the solvent removed *in vacuo*. The aqueous phase was then extracted with ethyl acetate (3 × 40 mL). The combined organic phase was then washed with brine (1 × 60 mL), dried with sodium sulfate and the solvent removed *in vacuo* to give a yellow-brown oil. The yellow oil was then dissolved into a 50:50 mixture of ethyl acetate and hexane and passed through a plug of silica, followed again by solvent removal *in vacuo* resulting in the aldehyde **56** as a light-yellow oil (1.93 g, 30%). **^{1}H NMR** (400 MHz, $CDCl_3$): δ 9.52 (1H, s), 4.64-4.38 (1H, m), 4.04-3.73 (1H, m), 2.99-2.68 (1H, m), 2.16-2.04 (1H, m), 1.67-1.47 (3H, m), 1.44-1.30 (1H, m), 1.40 (9H, s), 1.26-1.15 (1H, m) ppm; **^{13}C NMR** (100 MHz, $CDCl_3$): δ 201.4 (CH), 155.7 (C_q), 80.5 (C_q), 61.2 (CH), 42.6 (CH_2), 28.4 (CH_3), 24.8 (CH_2), 23.7 (CH_2), 21.0 (CH_2) ppm; **HRMS** (ESI): *m/z* $[M+Na]^+$ calculated for $C_{11}H_{19}NNaO_3^+$ 236.1257, found 236.1259.

***tert*-Butyl 2-((hydroxyimino)methyl) piperidine-1-carboxylate 57**

To a solution of aldehyde **56** (1.80 g, 8.44 mmol, 1 eq.) in methanol (8 mL) at 0 °C was added hydroxylamine hydrochloride (0.645 g, 9.28 mmol, 1.1 eq.) and sodium bicarbonate (0.92 g, 11.0 mmol, 1.3 eq.). The reaction was then warmed to room temperature and stirred for 16 hours. Upon completion the solvent was removed *in vacuo* and the resultant slurry dissolved into brine (8 mL) and extracted with ethyl acetate (4 × 15 mL). The combined organic phase was then passed through a silica plug, dried with sodium sulfate and the solvent removed *in vacuo* producing the oxime **57** as a clear oil (1.80 g, 93%). **^{1}H NMR** (400 MHz, $CDCl_3$, exists as a 0.4:1 mixture of isomers): δ 9.04 (1H, br s, min.), 8.7 (1H, br s, maj.), 7.33 (1H, d, J = 3.5 Hz, maj.), 6.80 (1H, d, J = 5.5 Hz, min.), 5.35 (1H, m, min.), 4.88 (1H, m, maj.), 4.03 (1H, d, J = 13.5 Hz), 3.95 (1H, d, J = 13.0 Hz, maj.), 2.95-2.84 (1H, m, min.), 2.84-2.75 (1H, m, maj.), 2.08-2.02 (1H, m, min.), 1.94-1.87 (1H, m, maj.), 1.70-1.53 (8H, m, maj. + min.), 1.52-1.30 (2H, m, maj. + min.), 1.42 (9H, s, maj.), 1.40 (9H, s, min.) ppm; **^{13}C NMR** (100 MHz, $CDCl_3$, exists as a 0.4:1 mixture of isomers): δ 155.4 (C_q, maj.), 155.3 (C_q, min.), 151.0 (CH, min.), 150.3 (CH, maj.), 80.22 (C_q, min.), 80.18 (C_q, maj.), 50.5 (CH, min.), 48.0 (CH, maj.), 40.9 (CH_2, maj. + min.), 28.51 (CH_3, maj.), 28.50 (CH_3, min.), 27.4 (CH_2, maj.), 27.0 (CH_2, min.), 25.3 (CH_2, maj. + min.), 21.1 (CH_2, min.), 19.9 (CH_2, maj.) ppm; **HRMS** (ESI): *m/z* $[M+H]^+$ calculated for $C_{11}H_2N_2NaO_3^+$ 251.1366, found 251.1367.

***tert*-Butyl 2-(chloro(hydroxyimino) methyl) piperidine-1-carboxylate 58**

To a solution of oxime **57** (1.80 g, 7.89 mmol, 1 eq.) in dimethylformamide (10 mL) at 0 °C was added 4M HCl in dioxane (0.197 mL, 0.79 mmol, 0.1 eq.) followed by *N*-chlorosuccinimide (1.13 g, 8.44 mmol, 1.1 eq.) whilst maintaining the temperature at 0 °C. The reaction was then stirred at room temperature for 16 hours. Upon completion the reaction was diluted with water (25 mL) and extracted with ethyl acetate (4 × 10 mL). The combined organic extracts were then washed with water (1 × 20 mL), brine (1 × 20 mL), dried with sodium sulfate and the solvent removed *in vacuo* giving chlorooxime **58** as a light-yellow solid (1.49 g, 72%). **^{1}H NMR** (400 MHz, $CDCl_3$): δ 10.04 (1H, br s), 4.88 (1H, br s.), 3.95-3.87 (1H, m), 2.89-2.80 (1H, m), 2.24-2.15 (1H, m), 1.68-1.53 (3H, m), 1.50-1.32 (2H, m), 1.41 (9H, s) ppm; **^{13}C NMR** (100 MHz, $CDCl_3$): δ 156.0 (C_q), 138.5 (C_q), 80.9 (C_q), 56.0 (CH), 41.0 (CH_2), 28.6 (CH_3), 26.4 (CH_2), 24.8 (CH_2), 19.3 (CH_2) ppm.

5-Isopropyl-3-(piperidin-2-yl) isoxazole hydrochloride 59

General procedure 1 using chlorooxime **58** (0.500 g, 1.90 mmol) and 3-methyl-1-butyne gave isoxazole **59** as a brown oil (0.149 g, 51% over 2 steps). **^{1}H NMR** (400 MHz, $CDCl_3$): δ 5.93 (1H, s), 3.82-3.76 (1H, m), 3.13-3.05 (1H, m), 2.98 (1H, sept., *J* = 7.0 Hz), 2.77-2.68 (1H, m), 2.52 (1H, br s), 1.88-1.79 (2H, m), 1.65-1.40 (4H, m), 1.23 (6H, d, *J* = 7.0 Hz) ppm; **^{13}C NMR** (100 MHz, $CDCl_3$): δ 178.7 (C_q), 166.6 (C_q), 97.0 (CH), 53.7 (CH), 47.0 (CH_2), 32.1 (CH_2), 27.3 (CH), 25.8 (CH_2), 24.5 (CH_2), 20.9 (CH_3) ppm; **HRMS** (ESI): *m/z* $[M+H]^+$ calculated for $C_{11}H_{19}N_2O^+$ 195.1492, found 195.1497; **RP-HPLC**: t_R 9.5 mins (Method A).

3-(1-((1-Ethyl-1*H*-indol-3-yl) methyl) piperidin-2-yl)-5-isopropylisoxazole BR43

General procedure 4 using isoxazole **59** (0.149 g, 0.77 mmol) and indole **15** gave product **BR43** as a light brown oil (0.071 g, 26%). **^{1}H NMR** (400 MHz, $CDCl_3$): δ 7.67 (1H, d, *J* = 8.0 Hz), 7.32 (1H, d, *J* = 8.0 Hz), 7.20 (1H, t, *J* = 8.0 Hz), 7.12-7.08 (2H, m), 6.20 (1H, s), 4.15 (2H, q, *J* = 7.0 Hz), 3.90 (1H, d, *J* = 14.0 Hz)m 3.53 (1H, d, *J* = 10.0 Hz), 3.40 (1H, d, *J* = 14.0 Hz), 3.16-3.11 (1H, m), 3.10 (1H, sept., *J* = 7.0 Hz), 2.13-2.03 (1H, m), 1.87-1.68 (3H, m), 1.66-1.55 (2H, m), 1.45 (3H, t, *J* = 7.0 Hz), 1.35 (6H, d, *J* = 7.0 Hz) ppm; **^{13}C NMR** (100 MHz, $CDCl_3$): δ 179.0 (C_q), 167.5 (C_q), 136.1 (C_q), 129.1 (C_q), 127.6 (CH), 121.4 (CH), 119.9 (CH), 119.0 (CH), 110.1 (C_q), 109.4 (CH), 97.6 (CH), 58.7 (CH), 52.7 (CH_2), 50.3 (CH_2), 41.0 (CH_2), 33.7 (CH_2), 29.9 (CH_2), 27.5 (CH), 25.8 (CH_2), 24.2 (CH_2), 21.1 (CH_3), 15.6 (CH_3) ppm; **HRMS** (ESI): *m/z* $[M+H]^+$ calculated for $C_{22}H_{30}N_3O^+$ 352.2383, found 352.2389; **RP-HPLC**: t_R 15.9 mins (Method A)

***tert*-Butyl 5-chloro-3-formyl-1*H*-indole-1-carboxylate 62**

To a solution of compound **60**, dimethylformamide (3.07 mL, 39.6 mmol, 6 eq.) in dichloromethane (33 mL) at 0 °C was added a solution of phosphorus oxychloride (1.84 mL, 19.8 mmol, 3 eq.) in dichloromethane (11.9 mL). The mixture was then heated to reflux for 30 minutes, upon which a

solution of 5-chloroindole (1.00 g, 6.60 mmol, 1 eq.) in dichloromethane (33 mL) was added and the reaction left to reflux for a further one hour. Upon completion the reaction was cooled, water (50 mL) was added, and the pH adjusted to eight using potassium carbonate. The mixture was then extracted with chloroform (3 × 25 mL). The combined organic phases were then washed with brine (1 × 40 mL), dried with sodium sulfate and the solvent removed *in vacuo*. The resultant solid, di-*tert*-butyl dicarbonate (1.59 g, 7.26 mmol, 1.1 eq.) and 4-dimethylaminopyridine (0.081 g, 0.660 mmol, 0.1 eq.) were then dissolved into dry tetrahydrofuran (30 mL) under an atmosphere of nitrogen and the reaction left to stir for one hour. Upon completion the solvent was removed *in vacuo*, the resultant slurry dissolved into ethyl acetate (50 mL), washed with 1M hydrochloric acid (1 × 25 mL), brine (1 × 25 mL), dried with sodium sulfate and the solvent removed *in vacuo* giving Boc formyl indole **6.02** as a white solid (1.76 g, 95%). **^{1}H NMR** (400 MHz, $CDCl_3$): δ 10.06 (1H, s), 8.28 (1H, s), 8.22 (1H, s), 8.06 (1H, d, *J* = 9.0 Hz), 7.36 (1H, dd, *J* = 9.0, 2.0 Hz), 1.70 (9H, s) ppm; **^{13}C NMR** (100 MHz, $CDCl_3$): δ 185.3 (CH), 148.1 (C_q), 136.8 (CH), 134.2 (C_q), 130.5 (C_q), 127.0 (C_q), 126.3 (CH), 121.7 (CH), 120.6 (C_q), 116.0 (CH), 86.0 (C_q), 28.0 (CH_3) ppm; **LRMS** (ESI): *m/z* 280.2 $(M+H)^+$.

***tert*-Butyl 6-chloro-3-formyl-1*H*-indole-1-carboxylate 63**

To a solution of compound **61**, dimethylformamide (2.76 mL, 35.6 mmol, 6 eq.) in dichloromethane (29.7 mL) at 0 °C was added a solution of phosphorus oxychloride (1.66 mL, 17.8 mmol, 3 eq.) in dichloromethane (10.7 mL). The mixture was then heated to reflux for 30 minutes, upon which a solution of 6-chloroindole (0.90 g, 5.94 mmol, 1 eq.) in dichloromethane (29.7 mL) was added and the reaction left to reflux for a further one hour. Upon completion the reaction was cooled, water (50 mL) was added, and the pH adjusted to eight using potassium carbonate. The mixture was then extracted with chloroform (3 × 25 mL). The combined organic phases were then washed with brine (1 × 40 mL), dried with sodium sulfate and the solvent removed *in vacuo*. The resultant solid, di-*tert*-butyl dicarbonate (1.43 g, 6.53 mmol, 1.1 eq.) and 4-dimethylaminopyridine (0.073 g, 0.594 mmol, 0.1 eq.) were then dissolved into dry tetrahydrofuran (27 mL) under an atmosphere of nitrogen and the reaction left to stir for one hour. Upon completion the solvent was removed *in vacuo*, the resultant slurry dissolved into ethyl acetate (50 mL), washed with 1M hydrochloric acid (1 × 25 mL), brine (1 × 25 mL), dried with sodium sulfate and the solvent removed *in vacuo* giving Boc formyl indole **6.03** as a white solid (1.43 g, 86%). **^{1}H NMR** (400 MHz, $CDCl_3$): δ 10.07 (1H, s), 8.21-8.19 (3H, m), 7.35 (1H, dd, *J* = 8.5, 2.0 Hz), 1.71 (9H, s) ppm; **^{13}C NMR** (100 MHz, $CDCl_3$): δ 185.7 (CH), 136.8 (C_q), 136.5 (CH), 132.3 (C_q), 125.5 (CH), 124.8 (C_q), 123.1 (CH), 121.5 (C_q), 115.8 (CH), 86.5 (C_q), 28.3 (CH_3) ppm; **LRMS** (ESI): *m/z* 280.3 $(M+H)^+$.

(*S*)-3-(1-((5-Chloro-1*H*-indol-3-yl) methyl) pyrrolidin-2-yl)-5-isopropylisoxazole 64

Product was prepared by general procedure 4 using isoxazole **2** (0.305 g, 1.69 mmol) and indole **62** then dissolved into dichloromethane (17.0 mL) and trifluoroacetic acid (2.59 ml, 33.8 mmol, 20 eq.) added

at 0 °C and the reaction left to stir for three hours. Upon completion the mixture was neutralised with saturated potassium carbonate and the solution extracted with dichloromethane (2 × 10 mL). The combined organic extracts were then washed with water (1 × 10 mL), brine (1 × 10 mL), dried with sodium sulphate and the solvent removed *in vacuo* giving the product **6.04** as a brown oil (0.138 g, 24% over two steps). **^{1}H NMR** (400 MHz, $CDCl_3$): δ 8.69 (1H, br s), 7.54 (1H, s), 7.21 (1H, d, *J* = 8.5 Hz), 7.09 (1H, d, *J* = 8.5 Hz), 6.09 (1H, s), 3.96 (1H, d, *J* = 13.5 Hz), 3.69 (1H, t, *J* = 8.0 Hz), 3.51 (1H, d, *J* = 13.5 Hz), 3.13 (1H, t, *J* = 8.5 Hz), 3.06 (1H, sept., *J* = 7.0 Hz), 2.37 (1H, q, *J* = 8.5 Hz), 2.25-2.13 (1H, m), 1.96-1.84 (2H, m), 1.83-1.74 (1H, m), 1.33 (6H, d, *J* = 7.0 Hz) ppm; **^{13}C NMR** (100 MHz, $CDCl_3$): δ 179.2 (C_q), 166.9 (C_q), 134.8 (C_q), 129.1 (C_q), 125.4 (CH), 125.2 (C_q), 122.2 (CH), 119.0 (CH), 112.3 (CH + C_q), 97.3 (CH), 59.7 (CH), 53.7 (CH_2), 48.1 (CH_2), 32.2 (CH_2), 27.4 (CH), 22.8 (CH_2), 21.0 (CH_3) ppm; **HRMS** (ESI): *m/z* $[M+H]^+$ calculated for $C_{19}H_{23}ClN_3O^+$ 344.1524, found 344.1530; **RP-HPLC**: t_R 18.4 mins (Method).

(*S*)-3-(1-((6-Chloro-1*H*-indol-3-yl) methyl) pyrrolidin-2-yl)-5-isopropylisoxazole 65

Product was prepared by general procedure 4 using isoxazole **2** (0.250 g, 1.39 mmol) and indole **63** then dissolved into dichloromethane (14.0 mL) and trifluoroacetic acid (2.13 ml , 27.8 mmol, 20 eq.) added at 0 °C and the reaction left to stir for three hours. Upon completion the mixture was neutralised with saturated potassium carbonate and the solution extracted with dichloromethane (2 × 10 mL). The combined organic extracts were then washed with water (1 × 10 mL), brine (1 × 10 mL), dried with sodium sulfate and the solvent removed *in vacuo* giving the product **6.05** as a brown oil (0.257 g, 54 % over two steps). **^{1}H NMR** (400 MHz, $CDCl_3$): δ 8.18 (1H, br s), 7.51 (1H, d, *J* = 8.5 Hz), 7.28 (1H, s), 7.04 (1H, dd, *J* = 8.5, 2.0 Hz), 6.03 (1H, s), 3.96 (1H, d, *J* = 13.5 Hz), 3.66 (1H, t, *J* = 8.0 Hz), 3.53 (1H, d, *J* = 13.5 Hz), 3.11 (1H, t, *J* = 8.0 Hz), 3.04 (1H, sept, *J* = 7.0 Hz), 2.36 (1H, q, *J* = 8.0 Hz), 2.21-2.12 (1H, m), 1.93-1.82 (2H, m), 1.81-1.73 (1H, m), 1.30 (6H, d, *J* = 7.0 Hz) ppm; **^{13}C NMR** (100 MHz, $CDCl_3$): δ 179.0 (C_q), 166.9 (C_q) 136.8 (C_q), 128.0 (C_q), 126.7 (C_q), 124.7 (CH), 120.5 (CH), 120.3 (CH), 112.8 (C_q), 111.2 (CH), 97.3 (CH), 59.6 (CH), 53.7 (CH_2), 48.0 (CH_2), 32.2 (CH_2), 27.4 (CH), 22.9 (CH_2), 21.1 (CH_3) ppm; **HRMS** (ESI): *m/z* $[M+H]^+$ calculated for $C_{19}H_{23}ClN_3O^+$ 344.1524, found 344.1532; **RP-HPLC**: t_R 18.2 mins (Method A).

(*S*)-3-(1-((5-Chloro-1-ethyl-1*H*-indol-3-yl) methyl) pyrrolidin-2-yl)-5-isopropylisoxazole 66

Product was prepared by general procedure 3 using compound **64** (0.138 g, 0.401 mmol) giving the product **66** as a brown oil (0.139 g, 93%). **^{1}H NMR** (400 MHz, $CDCl_3$): δ 7.52 (1H, s), 7.18 (1H, d, *J* = 8.5 Hz), 7.11 (1H, dd, *J*= 8.5, 2.0 Hz), 7.04 (1H, s), 6.07 (1H, s), 4.08 (2H, q, *J* = 7.5 Hz), 3.93 (1H, d, *J* = 13.5 Hz), 3.69-3.63 (1H, m), 3.47 (1H, d, *J* = 13.5 Hz), 3.13 (1H, ,t, *J* = 8.5 Hz), 3.05 (1H, sept., *J* = 7.0 Hz), 2.35 (1H, q, *J* = 8.5 Hz), 2.25-2.13 (1H, m), 1.95-1.83 (2H, m), 1.83-1.74 (1H, m), 1.41 (3H, t, *J* = 7.5 Hz), 1.32 (1H, d, *J* = 7.0 Hz) ppm; **^{13}C NMR** (100 MHz, $CDCl_3$): δ 179.0 (C_q), 166.9 (C_q), 134.6 (C_q), 129.6 (C_q), 128.1 (CH), 124.9 (C_q), 121.7 (CH), 119.2 (CH), 111.2 (C_q), 110.3 (CH),

97.2 (CH), 59.8 (CH), 53.8 (CH_2), 48.0 (CH_2), 41.2 (CH_2), 32.2 (CH_2), 27.4 (CH), 22.8 (CH_2), 21.0 (CH_3), 15.5 (CH_3) ppm; **HRMS** (ESI): *m/z* $[M+H]^+$ calculated for $C_{19}H_{23}ClN_3O^+$ 344.1524, found 344.1532; **RP-HPLC**: t_R 18.9 mins (Method A).

(*S*)-3-(1-((6-Chloro-1-ethyl-1*H*-indol-3-yl) methyl) pyrrolidin-2-yl)-5-isopropylisoxazole 67

Product was prepared by general procedure 3 using product **65** (0.195 g, 0.567 mmol) giving product **6.07** as a brown oil (0.192 g, 91 %). **^{1}H NMR** (400 MHz, $CDCl_3$): δ 7.50 (1H, d, J = 8.5 Hz), 7.27 (1H, s), 7.03 (1H, dd, J = 8.5, 2.0 Hz), 7.01 (1H, s), 6.02 (1H, s), 4.07 (2H, q, J = 7.5 Hz), 3.95 (1H, d, J = 13.5 Hz), 3.66 (1H, t, J = 8.0 Hz), 3.51 (1H, d, J = 13.5 Hz), 3.12 (1H, t, J = 8.0 Hz), 3.04 (1H, sept., J = 7.0 Hz), 2.36 (1H, q, J = 8.0 Hz), 2.23-2.11 (1H, m), 1.95-1.72 (3H, m), 1.42 (3H, t, J = 7.5 Hz), 1.30 (1H, d, J = 7.0 Hz) ppm; **^{13}C NMR** (100 MHz, $CDCl_3$): δ 178.9 (C_q), 167.1 (C_q), 136.6 (C_q), 127.62 (C_q), 127.56 (CH), 127.3 (C_q), 120.7 (CH), 119.7 (CH), 117.7 (C_q), 109.4 (CH), 97.2 (CH), 59.6 (CH), 53.7 (CH_2), 47.9 (CH_2), 41.1 (CH_2), 32.2 (CH_2), 27.4 (CH), 22.8 (CH_2), 21.1 (CH_3), 15.6 (CH_3); **HRMS** (ESI): *m/z* $[M+H]^+$ calculated for $C_{19}H_{23}ClN_3O^+$ 344.1524, found 344.1532; **RP-HPLC**: t_R 19.2 mins (Method A).

Appendix Table S1: p values

Fig 1H

Comparison	Adjusted P Value
UT vs. 4-OHT	0.0005
UT vs. 4-OHT+C5	<0.0001
UT vs. 4-OHT+C23	0.0001
UT vs. 4-OHT+C24	>0.9999
UT vs. 4-OHT+C25	0.0214
UT vs. 4-OHT+C26	>0.9999
4-OHT vs. 4-OHT+C5	0.0378
4-OHT vs. 4-OHT+C23	0.9537
4-OHT vs. 4-OHT+C24	0.0007
4-OHT vs. 4-OHT+C25	0.3971
4-OHT vs. 4-OHT+C26	0.0003
4-OHT+C5 vs. 4-OHT+C23	0.1963
4-OHT+C5 vs. 4-OHT+C24	<0.0001
4-OHT+C5 vs. 4-OHT+C25	0.0009
4-OHT+C5 vs. 4-OHT+C26	<0.0001
4-OHT+C23 vs. 4-OHT+C24	0.0001
4-OHT+C23 vs. 4-OHT+C25	0.0904
4-OHT+C23 vs. 4-OHT+C26	<0.0001
4-OHT+C24 vs. 4-OHT+C25	0.0285
4-OHT+C24 vs. 4-OHT+C26	0.9994
4-OHT+C25 vs. 4-OHT+C26	0.0131

Ordinary one way-ANOVA with Tukey's multiple comparisons

Tukey's multiple comparisons test

BR43:10 μM vs. BR43:50 μM	0.0027	Two way anova with multiple comparisons

Fig 2C

Ordinary one-way ANOVA with multiple comparisons
Tukey's multiple comparisons test

Fig 3A

Heart rate

	Adjusted P Value
UT vs. ISO	0.4654
UT vs. ISO +BR43	0.1448
ISO vs. ISO +BR43	0.7373

Systolic volume

	Adjusted P Value
UT vs. ISO	0.0419
UT vs. ISO +BR43	0.9572
ISO vs. ISO +BR43	0.1201

Ejection fraction

	Adjusted P Value
UT vs. ISO	0.0491
UT vs. ISO +BR43	0.2792
ISO vs. ISO +BR43	0.0021

Diastolic volume

	Adjusted P Value
UT vs. ISO	0.2041
UT vs. ISO +BR43	0.4354
ISO vs. ISO +BR43	0.8709

Fig 3D

	Adjusted P Value
UT vs. Dox	<0.0001
UT vs. Dox + BR43	0.1343
UT vs. Dox +Atenolol	0.6313
Dox vs. Dox + BR43	<0.0001
Dox vs. Dox +Atenolol	<0.0001
Dox + BR43 vs. Dox +Atenolol	0.7219

Ordinary one-way ANOVA with multiple comparisons

Fig EV 5

One way ANOVA with multiple comparisons

Fig EV5

Tukey's multiple comparisons test — Adjusted P Value

Tukey's multiple comparisons test	RBC	HB	HT	RDW	MCH
UT vs. 0.26 MG/KG	0.9388	0.9942	0.998	0.6401	0.6549
UT vs. 0.53 MG/KG	0.8501	0.9641	0.9933	0.1782	0.2632
UT vs. 0.76 MG/KG	0.2875	0.981	0.3496	0.2592	0.2344

	MCV	PLT
UT vs. 0.26 MG/KG	0.5515	0.9677
UT vs. 0.53 MG/KG	0.225	0.989
UT vs. 0.76 MG/KG	0.9278	0.468
