# Supplementary material for: Development of a novel therapy for systolic heart failure
Source: EMBO Mol Med. 2025 Aug 4;17(9):2332–53. doi: 10.1038/s44321-025-00284-6 (PMC12423297; doi:10.1038/s44321-025-00284-6)
Supplement: Supplementary file 3 — Source data Fig. 1 [file 44321_2025_284_MOESM3_ESM.zip › Fig 1 source data_Original scans/Fig 1E/Fig 1e 2nd panel Bim, pCREB.pdf]

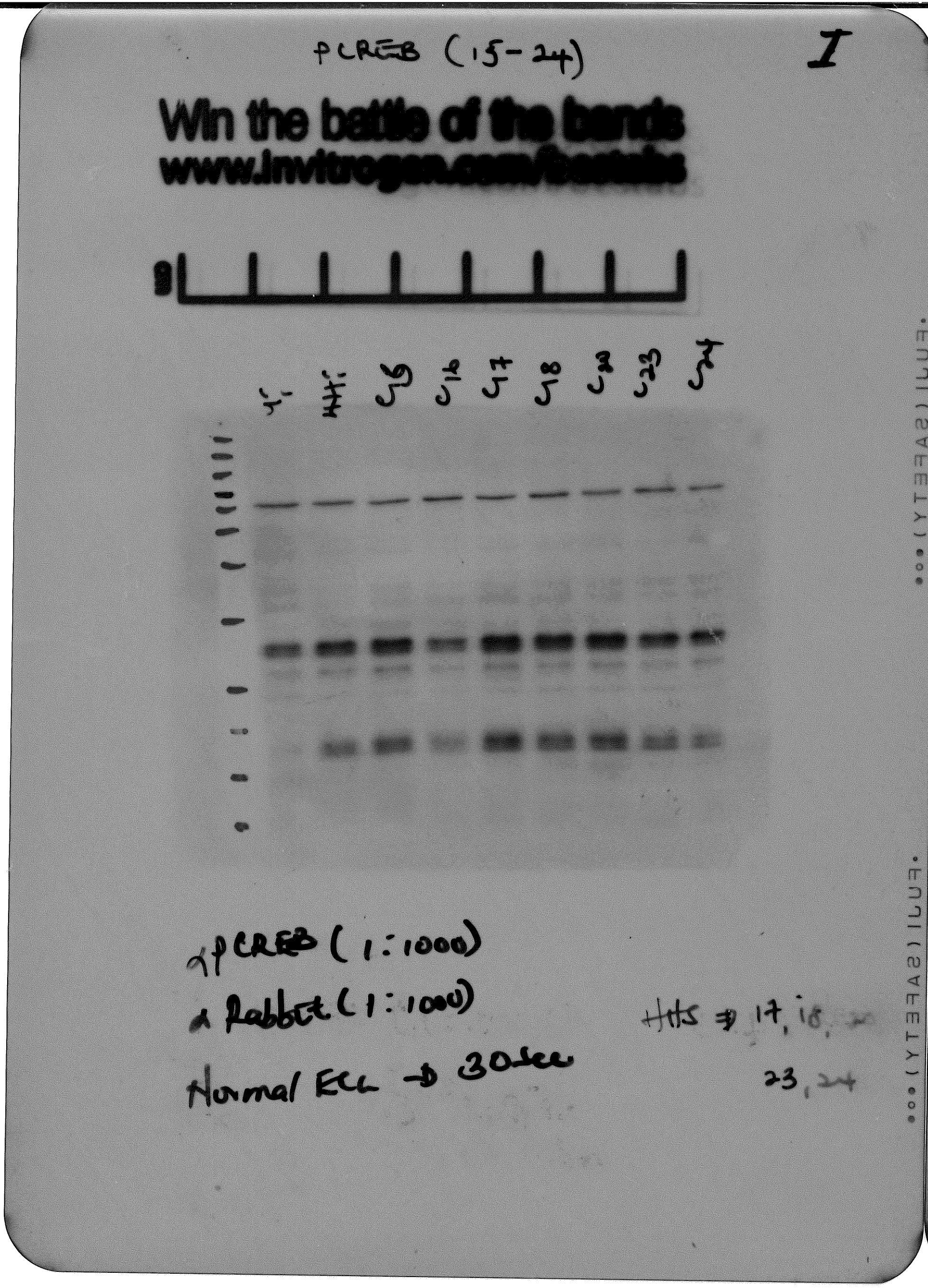
pCREB (15-24)
I
Win the battle of the bands
www.invitrogen.com/bestabs
Ui
HHi
C15
C16
C17
C18
C20
C23
C24
α pCREB (1:1000)
α Rabbit (1:1000)
Normal ECL → 30sec
Hits → 17, 18, 20
23, 24

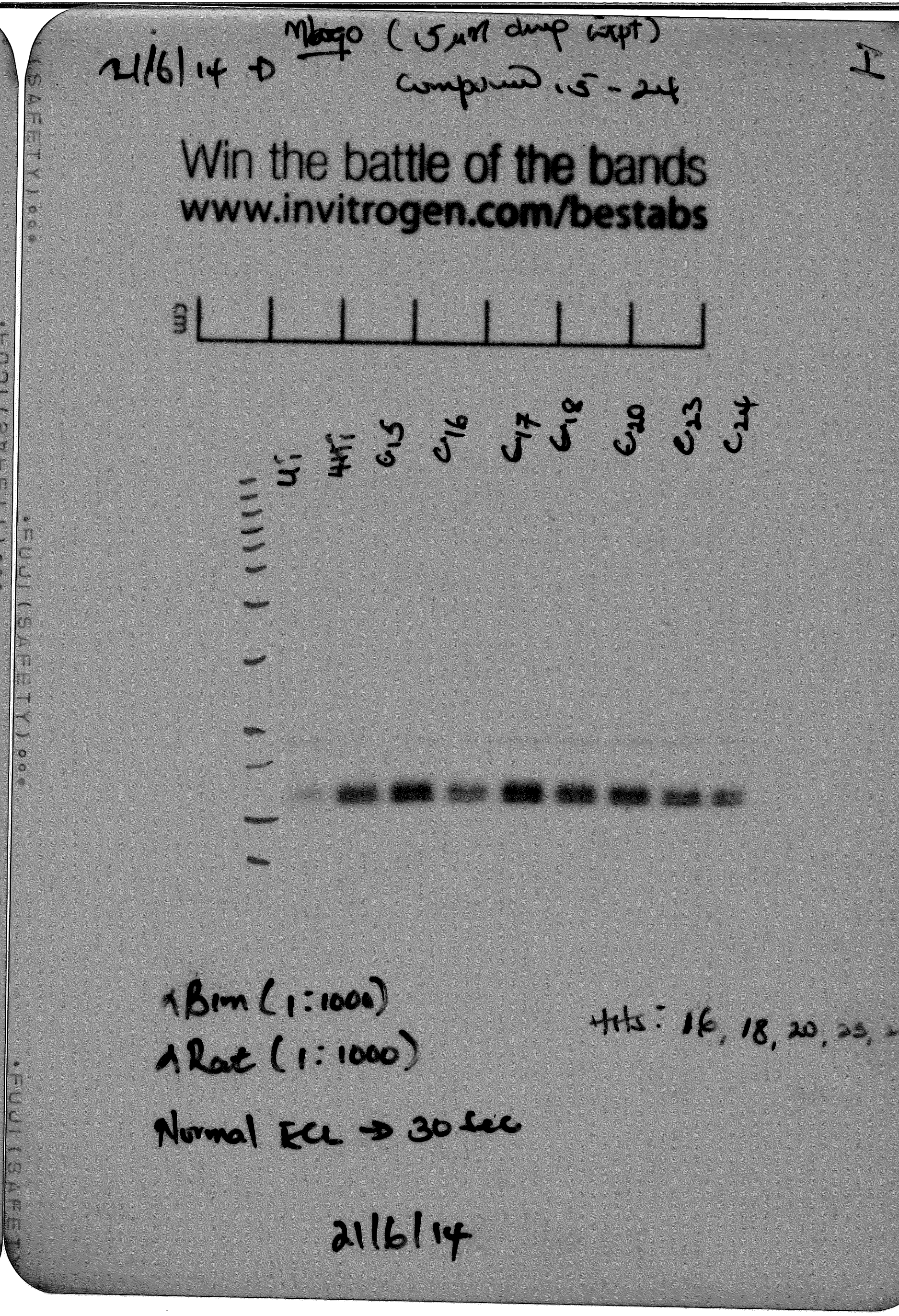
21/6/14 → Mbogo (5μM drug expt)
Compound 15 - 24
I
Win the battle of the bands
www.invitrogen.com/bestabs
cm
Ui
HHi
C15
C16
C17
C18
C20
C23
C24
α Bim (1:1000)
α Rat (1:1000)
Normal ECL → 30 sec
Hits: 16, 18, 20, 23, 24
21/6/14
