# Supplementary material for: Development of a novel therapy for systolic heart failure
Source: EMBO Mol Med. 2025 Aug 4;17(9):2332–53. doi: 10.1038/s44321-025-00284-6 (PMC12423297; doi:10.1038/s44321-025-00284-6)
Supplement: Supplementary file 3 — Source data Fig. 1 [file 44321_2025_284_MOESM3_ESM.zip › Fig 1 source data_Original scans/Fig 1E/Fig 1e 2nd panel HSP.pdf]

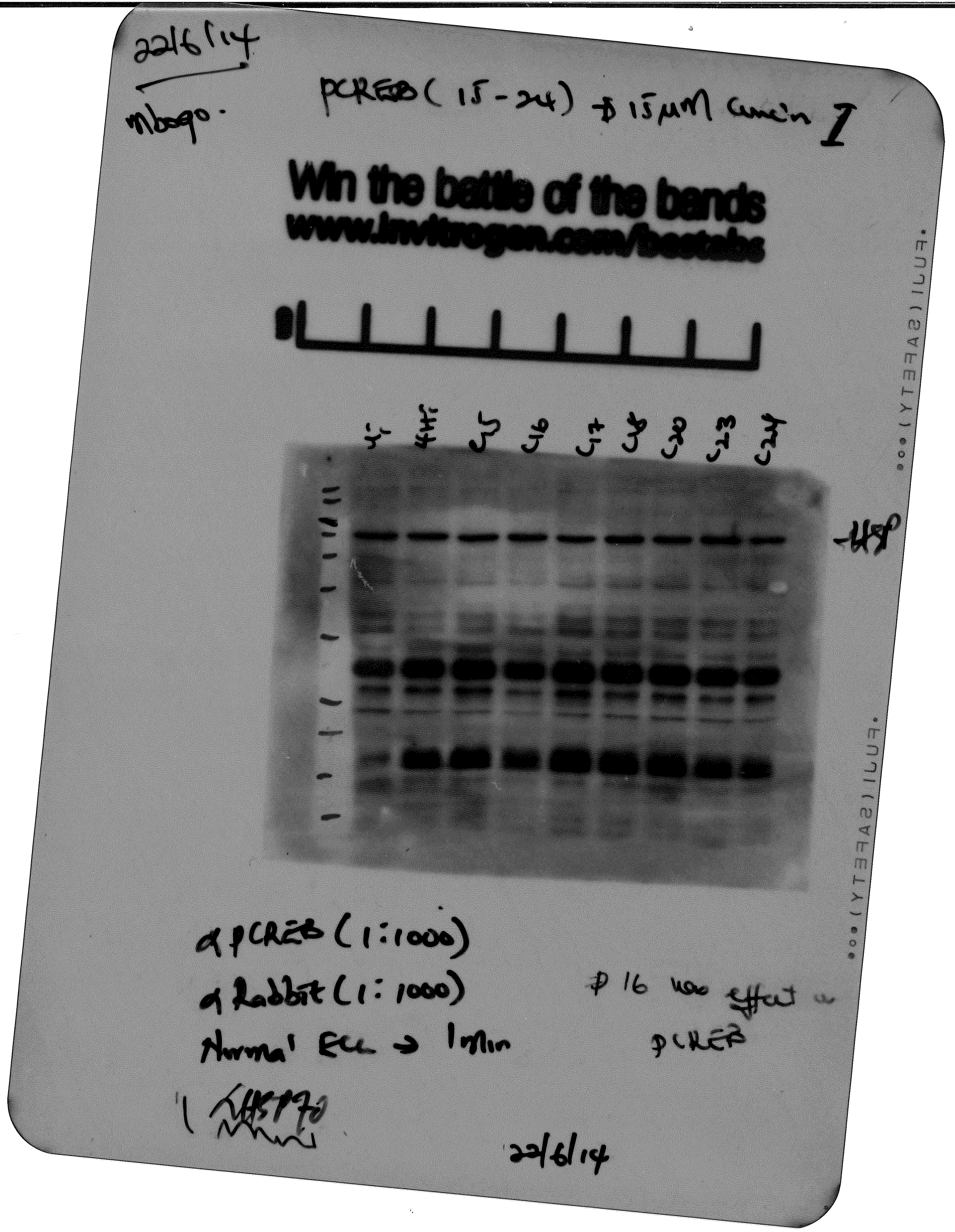
22/6/14
mbogo.
Win the battle of the bands
www.invitrogen.com/bestabs
α pCREB (1:1000)
α Rabbit (1:1000)
Normal ECL → 1 min
22/6/14
