# Supplementary figures and images for: Development of a novel therapy for systolic heart failure
Source: EMBO Mol Med. 2025 Aug 4;17(9):2332–53. doi: 10.1038/s44321-025-00284-6 (PMC12423297; doi:10.1038/s44321-025-00284-6)

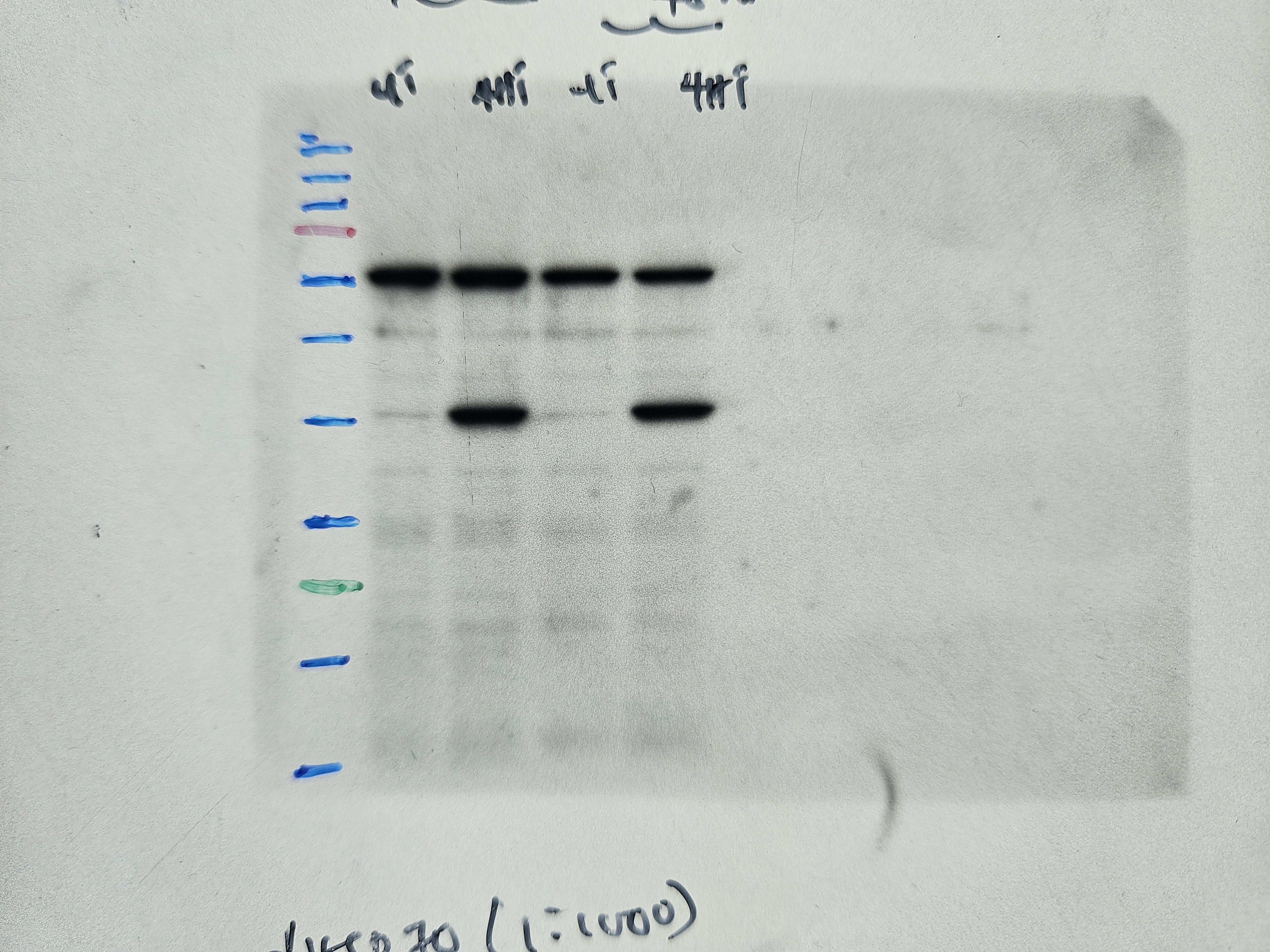

Supplement: Supplementary file 3 — Source data Fig. 1 [file 44321_2025_284_MOESM3_ESM.zip › Fig 1 source data_Original scans/Fig 1D/Fig 1d_HSP_PKAc.jpg]

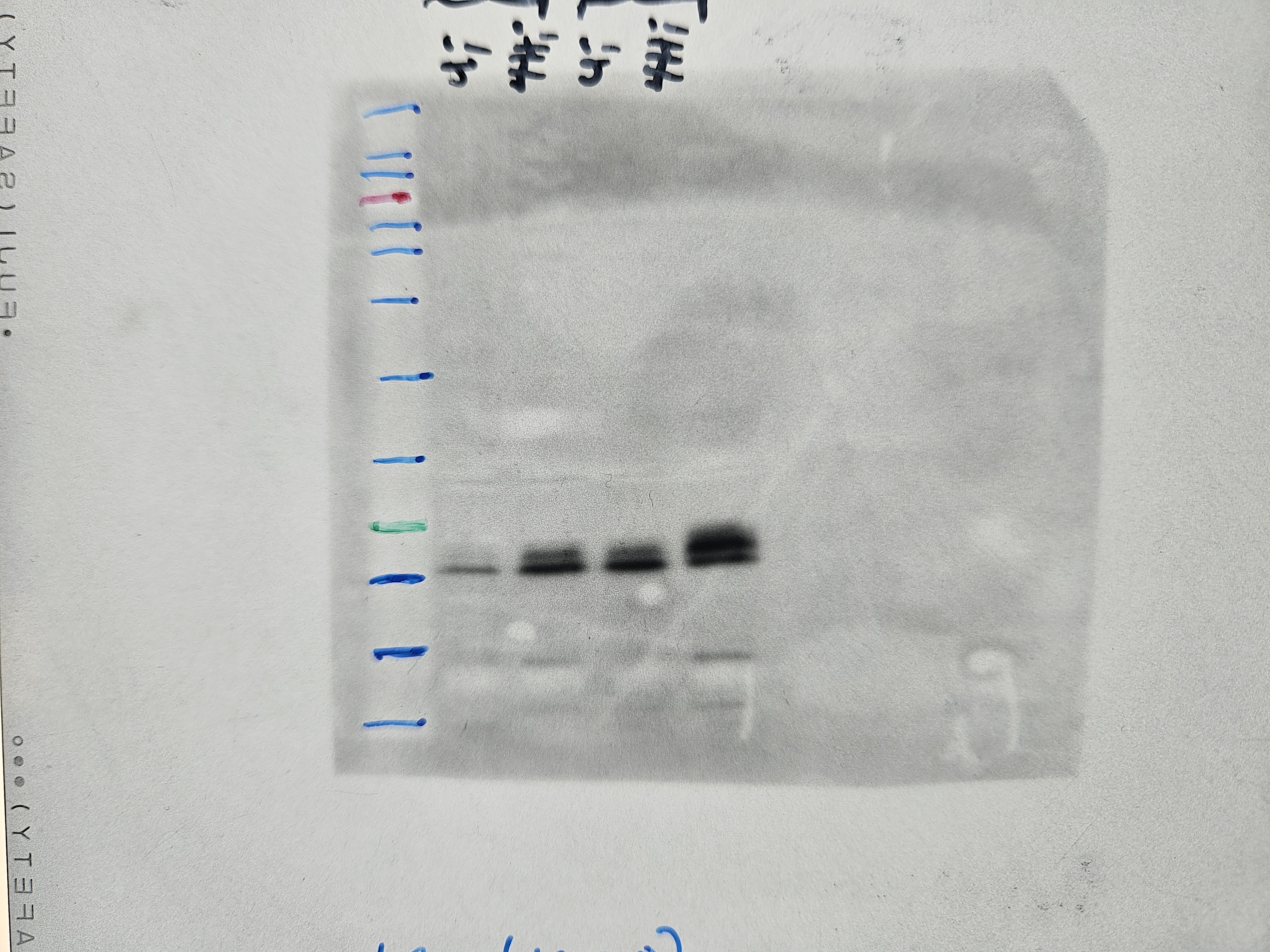

Supplement: Supplementary file 3 — Source data Fig. 1 [file 44321_2025_284_MOESM3_ESM.zip › Fig 1 source data_Original scans/Fig 1D/Fig1d_Bim.jpg]

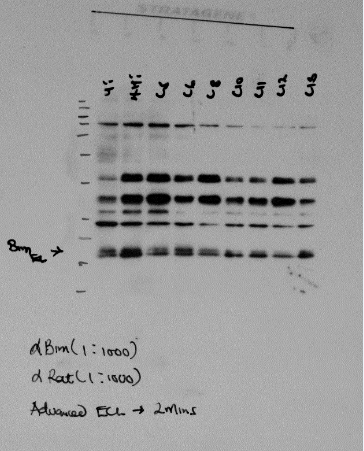

Supplement: Supplementary file 3 — Source data Fig. 1 [file 44321_2025_284_MOESM3_ESM.zip › Fig 1 source data_Original scans/Fig 1E/Fig 1e first panel Bim.PNG]

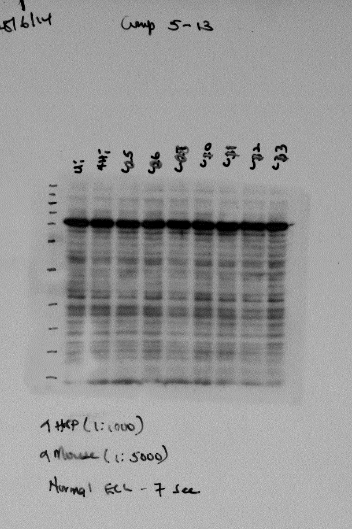

Supplement: Supplementary file 3 — Source data Fig. 1 [file 44321_2025_284_MOESM3_ESM.zip › Fig 1 source data_Original scans/Fig 1E/Fig 1e first panel HSP.PNG]

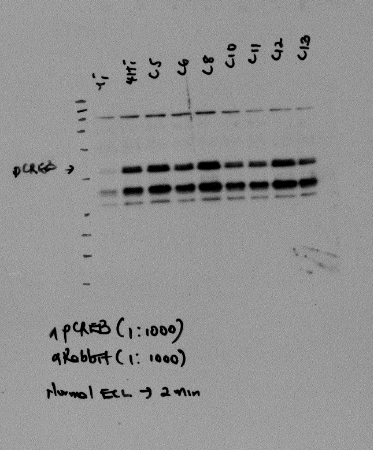

Supplement: Supplementary file 3 — Source data Fig. 1 [file 44321_2025_284_MOESM3_ESM.zip › Fig 1 source data_Original scans/Fig 1E/Fig 1e first panel pCREB.PNG]

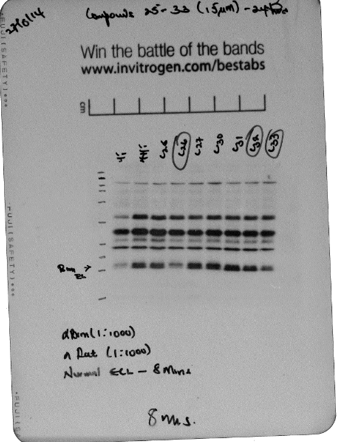

Supplement: Supplementary file 3 — Source data Fig. 1 [file 44321_2025_284_MOESM3_ESM.zip › Fig 1 source data_Original scans/Fig 1E/Fig 1e third panel Bim.PNG]

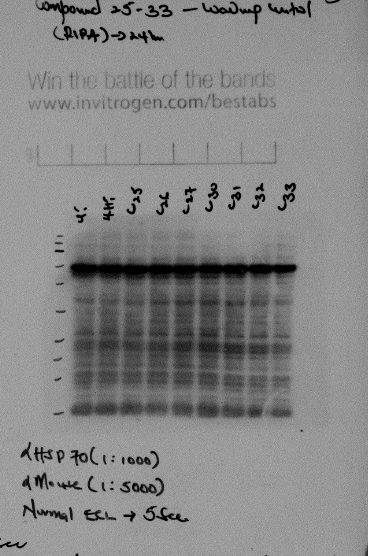

Supplement: Supplementary file 3 — Source data Fig. 1 [file 44321_2025_284_MOESM3_ESM.zip › Fig 1 source data_Original scans/Fig 1E/Fig 1e third panel HSP.PNG]

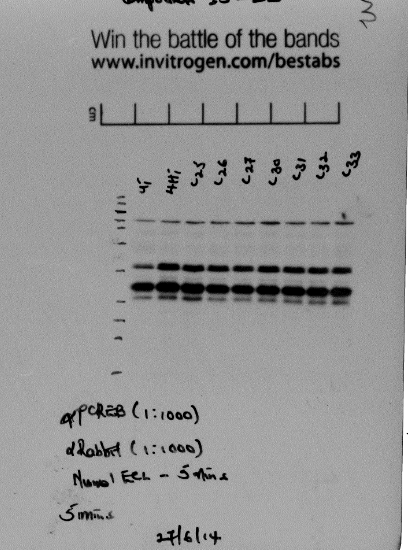

Supplement: Supplementary file 3 — Source data Fig. 1 [file 44321_2025_284_MOESM3_ESM.zip › Fig 1 source data_Original scans/Fig 1E/Fig 1e third panel pCREB.PNG]

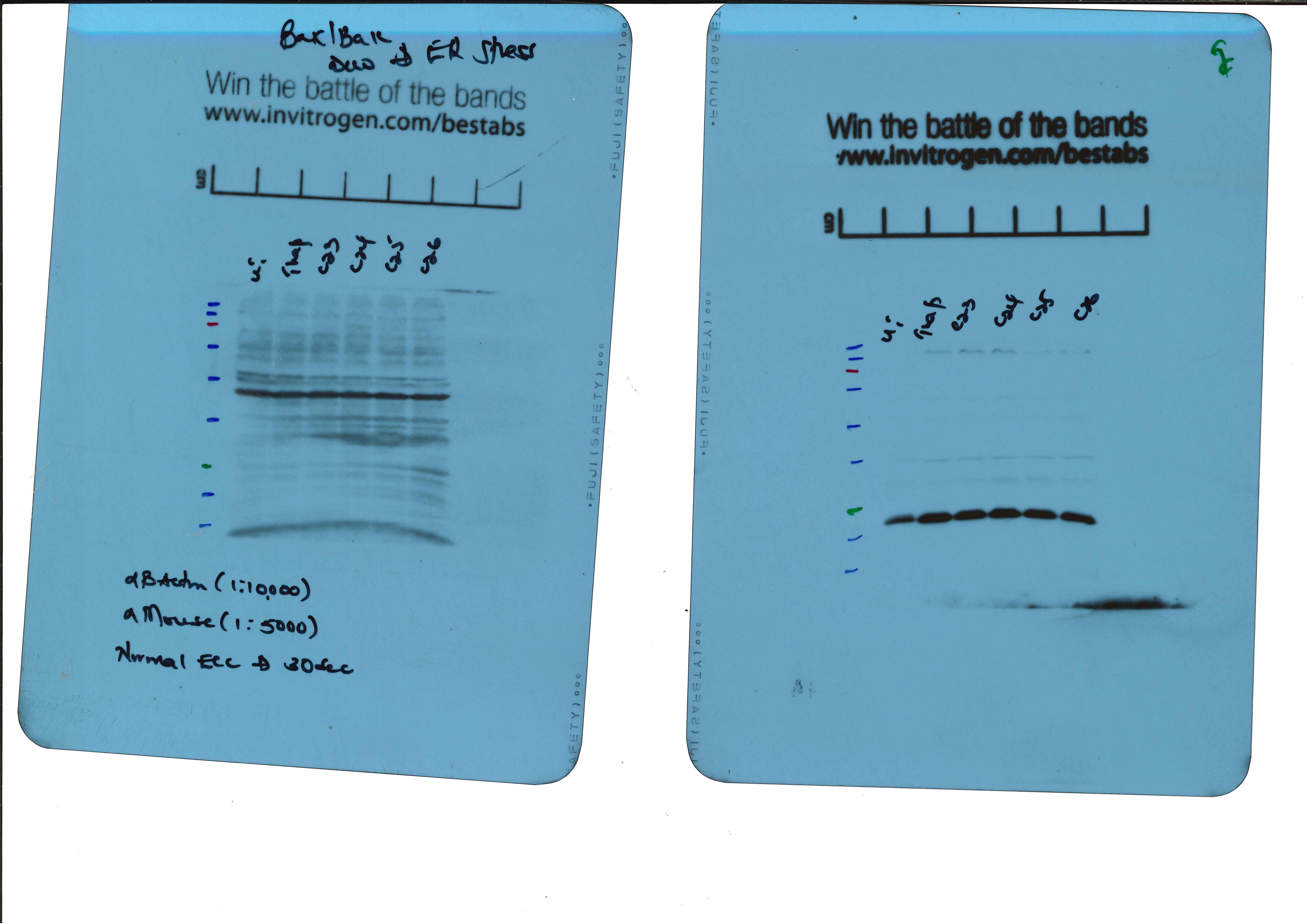

Supplement: Supplementary file 3 — Source data Fig. 1 [file 44321_2025_284_MOESM3_ESM.zip › Fig 1 source data_Original scans/Fig 1F/ERStress2.jpg]

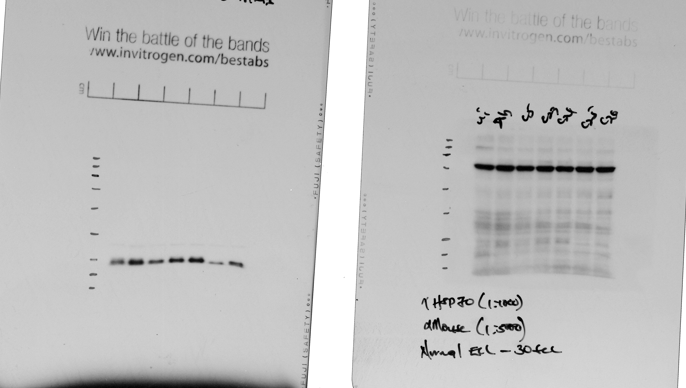

Supplement: Supplementary file 3 — Source data Fig. 1 [file 44321_2025_284_MOESM3_ESM.zip › Fig 1 source data_Original scans/Fig 1G/Fig 1g_Bim_HSP.jpg]

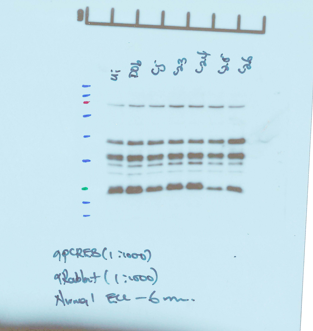

Supplement: Supplementary file 3 — Source data Fig. 1 [file 44321_2025_284_MOESM3_ESM.zip › Fig 1 source data_Original scans/Fig 1G/Fig1g_pCREB.jpg]

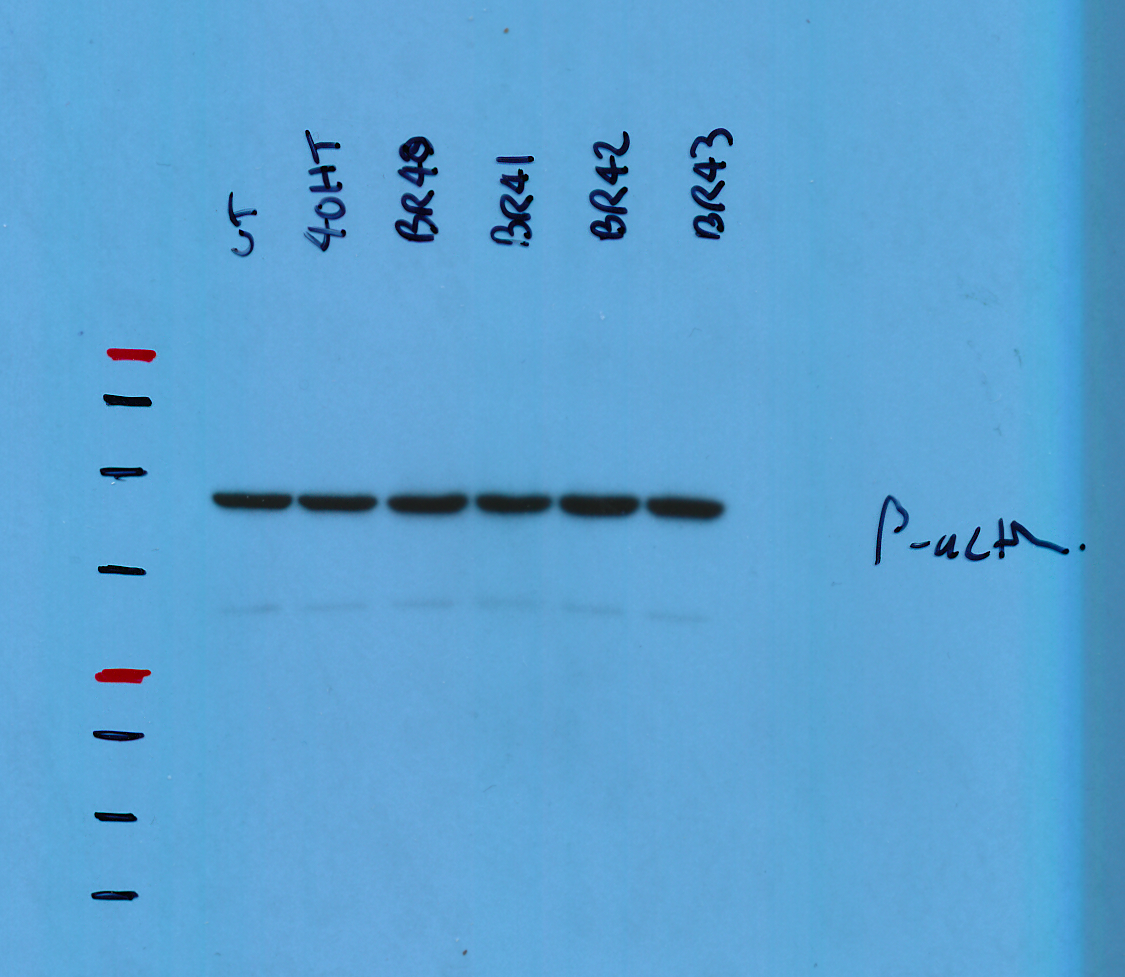

Supplement: Supplementary file 4 — Source data Fig. 2 [file 44321_2025_284_MOESM4_ESM.zip › Fig 2 source data_Original scans/Fig 2A Actin.png]

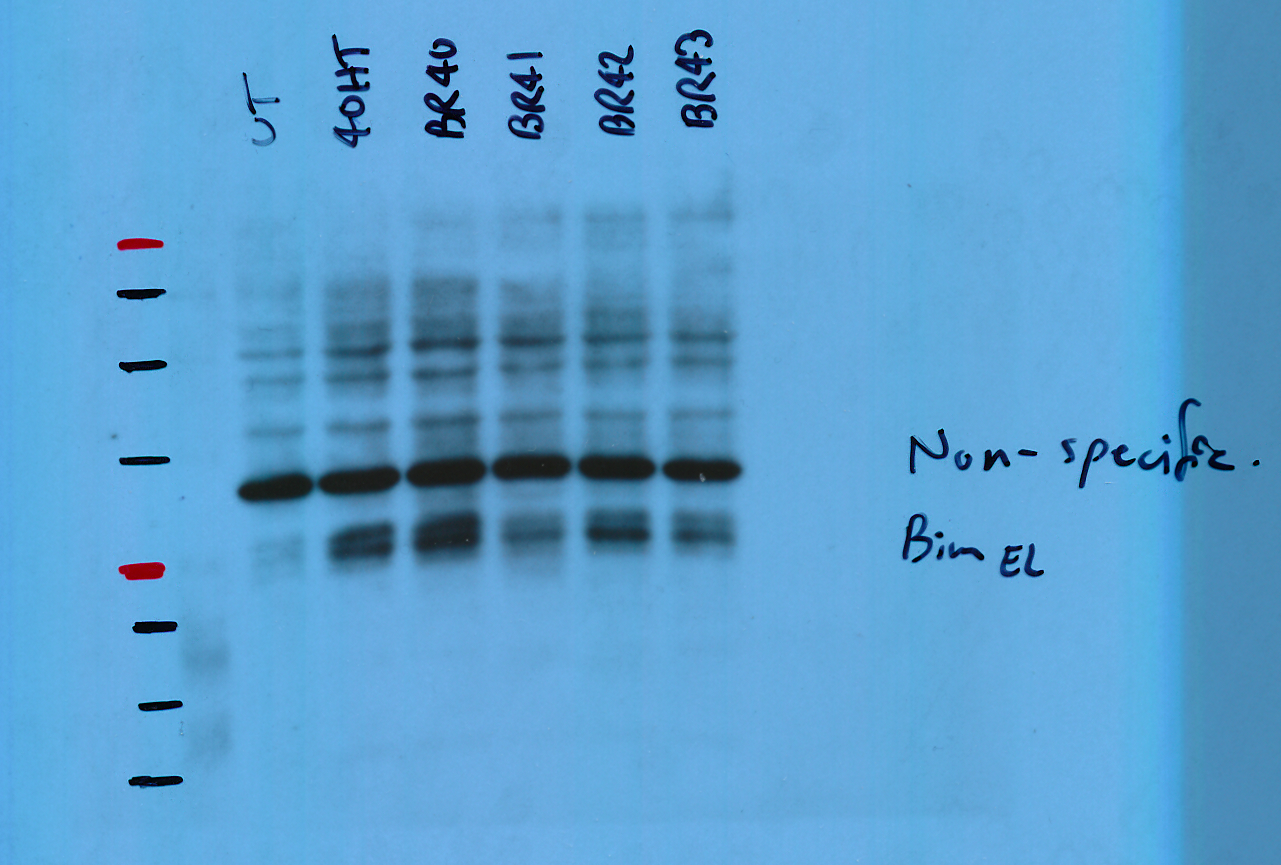

Supplement: Supplementary file 4 — Source data Fig. 2 [file 44321_2025_284_MOESM4_ESM.zip › Fig 2 source data_Original scans/Fig 2A_Bim.png]

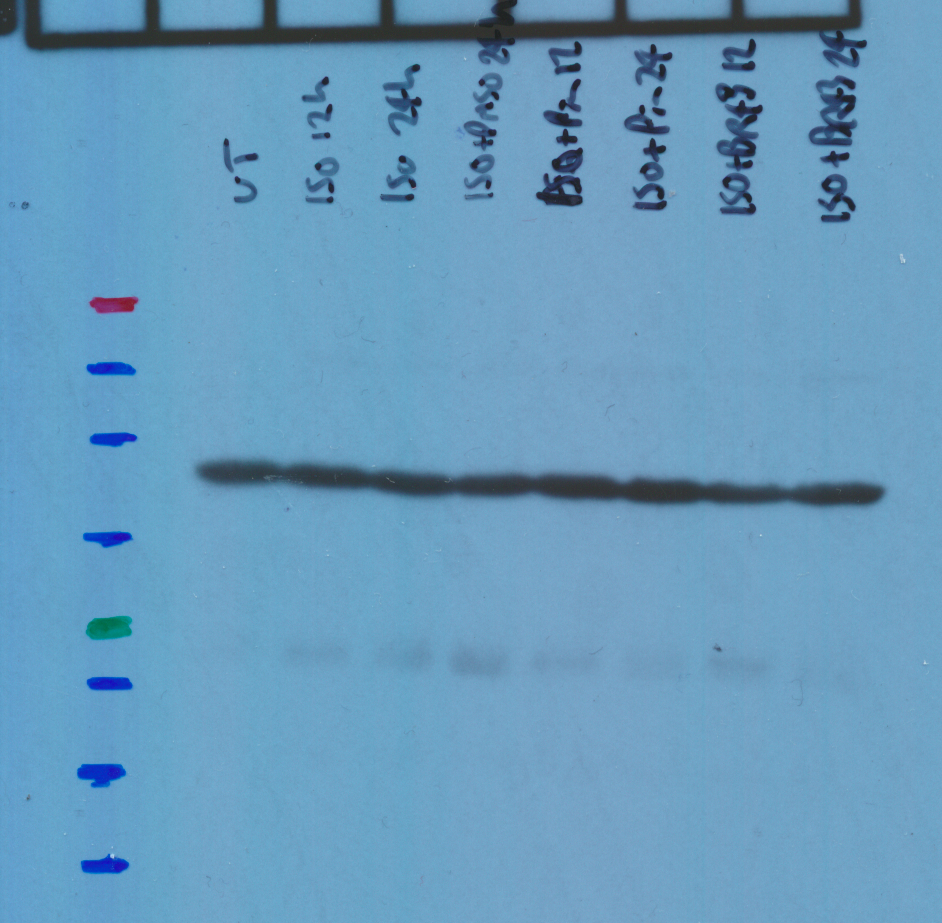

Supplement: Supplementary file 4 — Source data Fig. 2 [file 44321_2025_284_MOESM4_ESM.zip › Fig 2 source data_Original scans/Fig 2D_Actin.png]

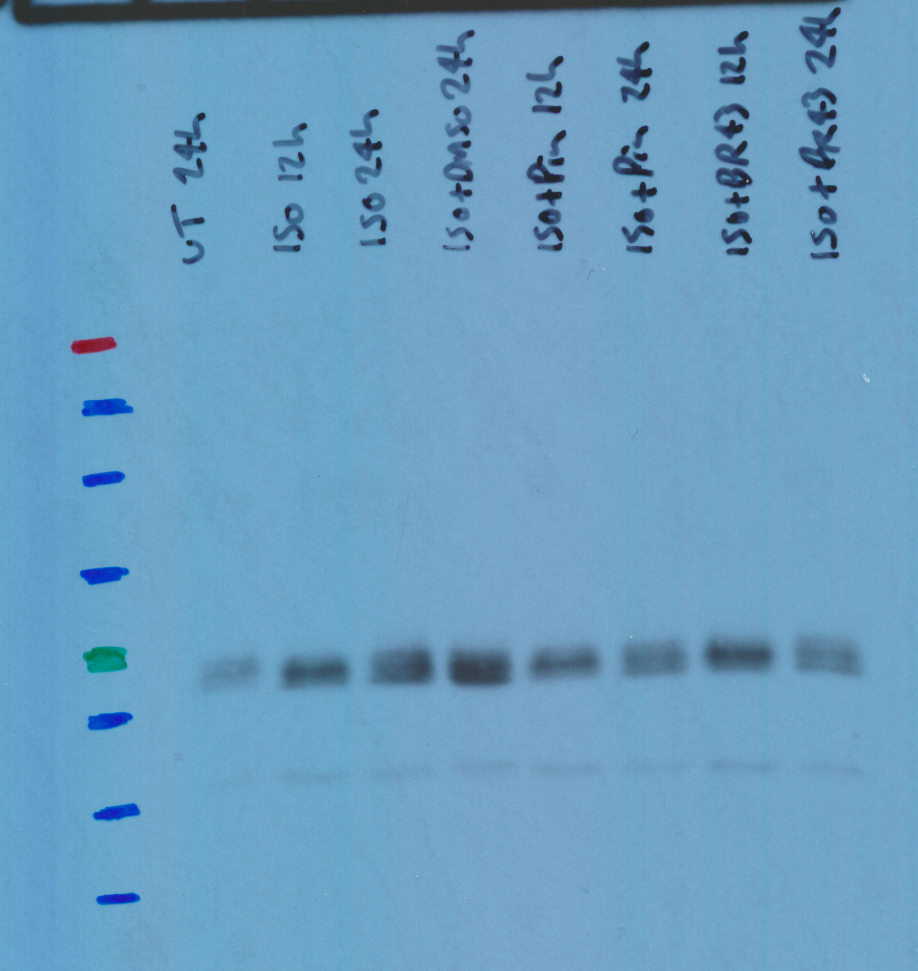

Supplement: Supplementary file 4 — Source data Fig. 2 [file 44321_2025_284_MOESM4_ESM.zip › Fig 2 source data_Original scans/Fig2D_Bim.png]

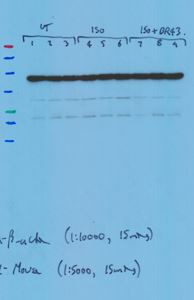

Supplement: Supplementary file 5 — Source data Fig. 3 [file 44321_2025_284_MOESM5_ESM.zip › Fig 3 source data_Original scans/Fig 3C_actin.JPG]

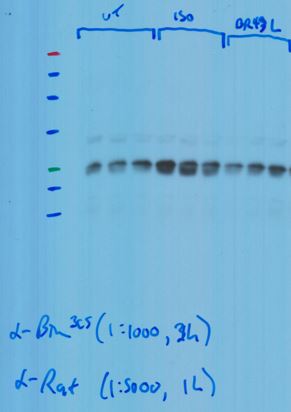

Supplement: Supplementary file 5 — Source data Fig. 3 [file 44321_2025_284_MOESM5_ESM.zip › Fig 3 source data_Original scans/Fig 3C_Bim.JPG]

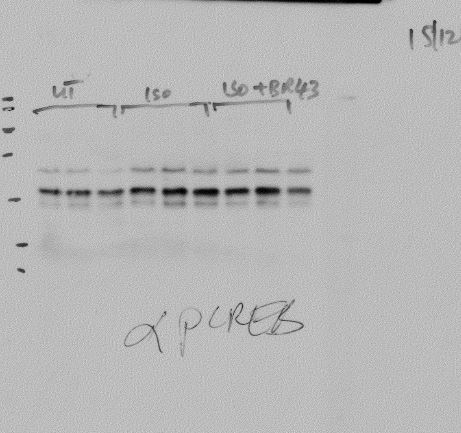

Supplement: Supplementary file 5 — Source data Fig. 3 [file 44321_2025_284_MOESM5_ESM.zip › Fig 3 source data_Original scans/Fig 3C_pCREB.JPG]
