# Supplementary material for: Development of a novel therapy for systolic heart failure
Source: EMBO Mol Med. 2025 Aug 4;17(9):2332–53. doi: 10.1038/s44321-025-00284-6 (PMC12423297; doi:10.1038/s44321-025-00284-6)
Supplement: Supplementary file 6 — Source data Fig. 4 [file 44321_2025_284_MOESM6_ESM.zip › Figure 4 Original scans pdf/4B Scans.pdf]

DMSO

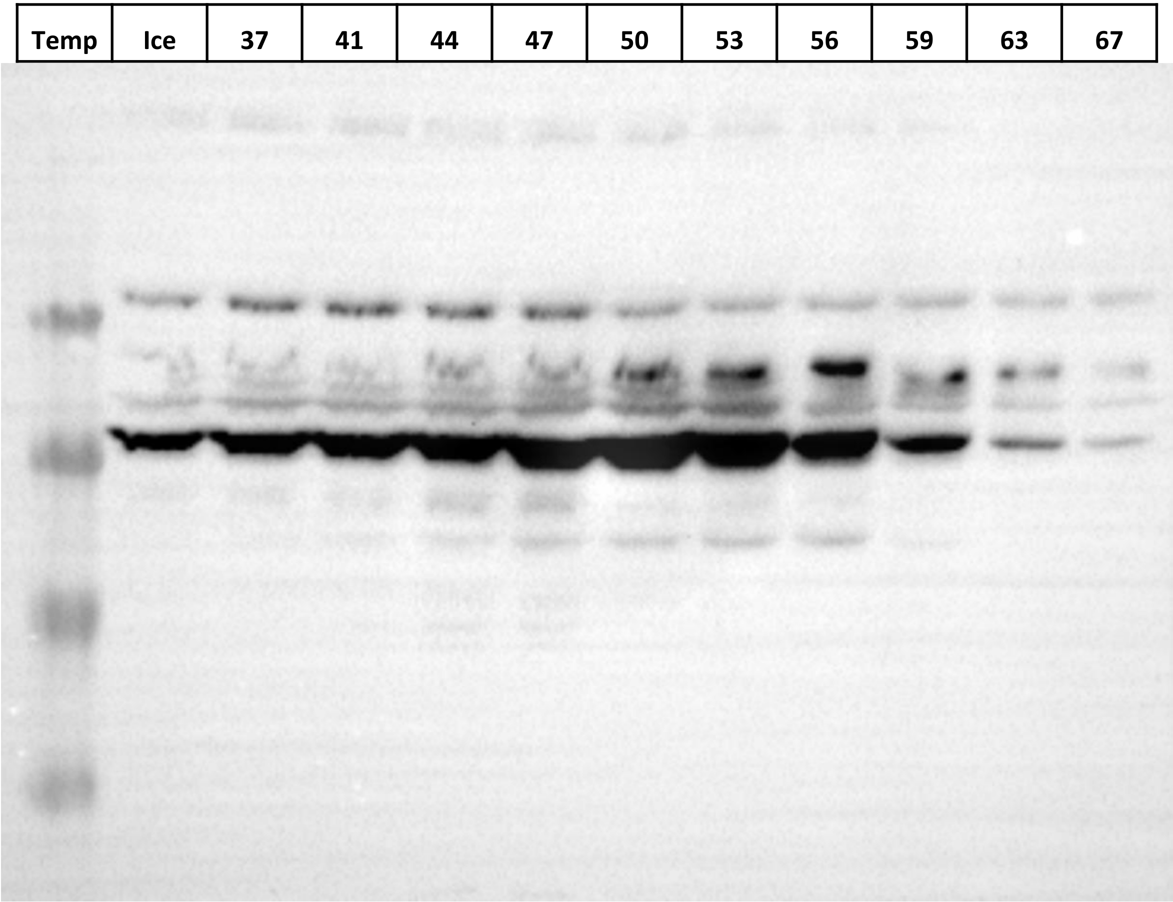

BR43

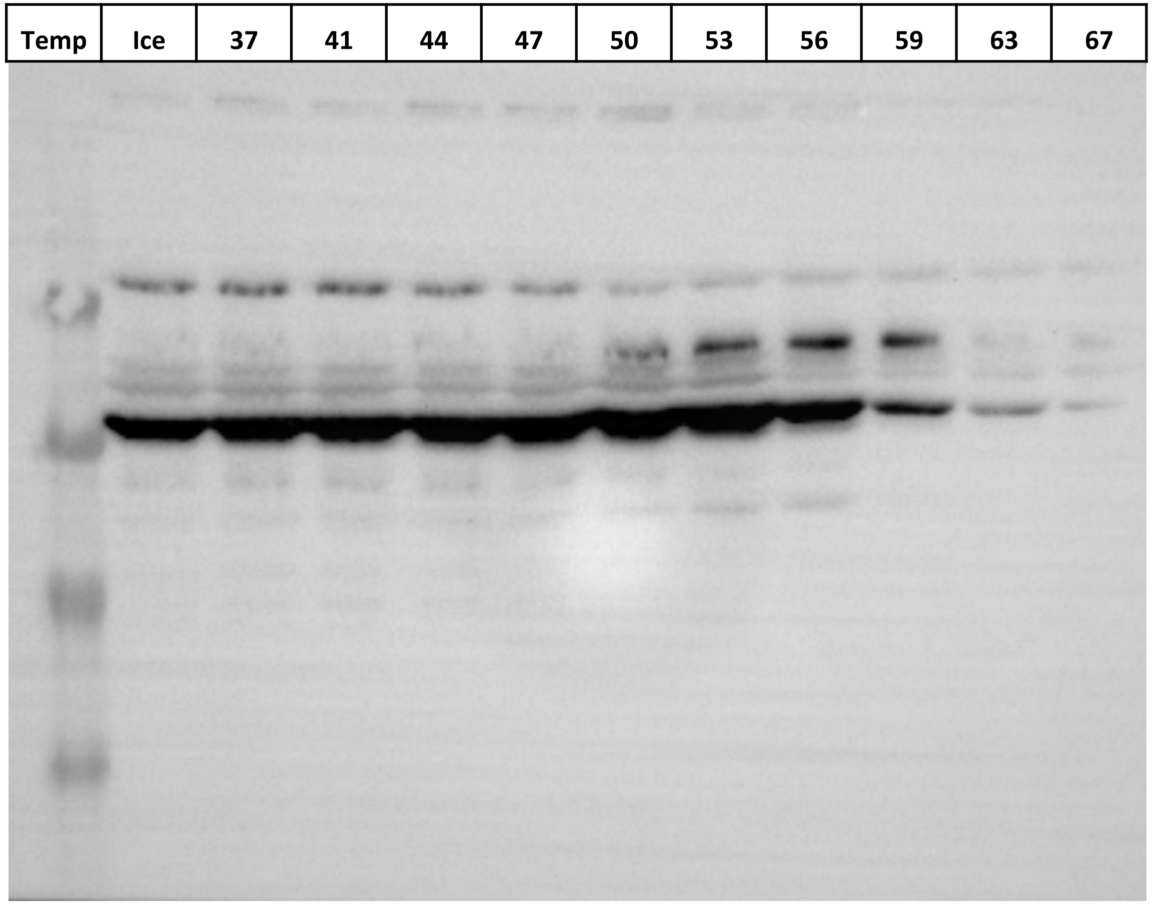

23 µL lysate/lane
10% gel, 1.5h 25V transfer
Anti-WDR3 1:1000 @ 4C, ON
Anti-Rabbit 1:5000 @ RT, 1h

WDR3

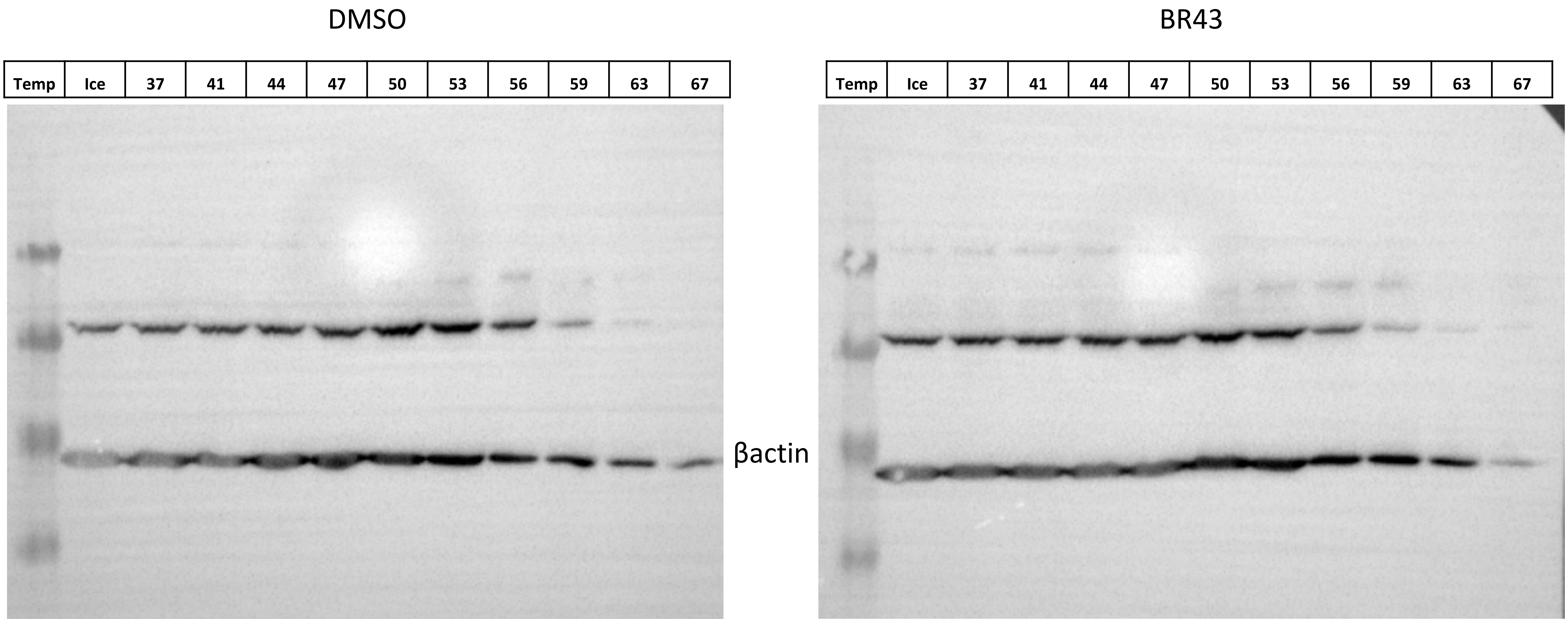

Anti-βactin 1:10000 @ 4C, ON
Anti-Mouse 1:5000 @ RT, 1h
