# Supplementary material for: Development of a novel therapy for systolic heart failure
Source: EMBO Mol Med. 2025 Aug 4;17(9):2332–53. doi: 10.1038/s44321-025-00284-6 (PMC12423297; doi:10.1038/s44321-025-00284-6)
Supplement: Supplementary file 6 — Source data Fig. 4 [file 44321_2025_284_MOESM6_ESM.zip › Figure 4 Original scans pdf/4D Scans.pdf]

| | Cas9 | Cas9 | 17 | 17 | 22 | 22 | 16.1 | 16.1 |
|---|---|---|---|---|---|---|---|---|
| 4HT | - | + | - | + | - | + | - | + |

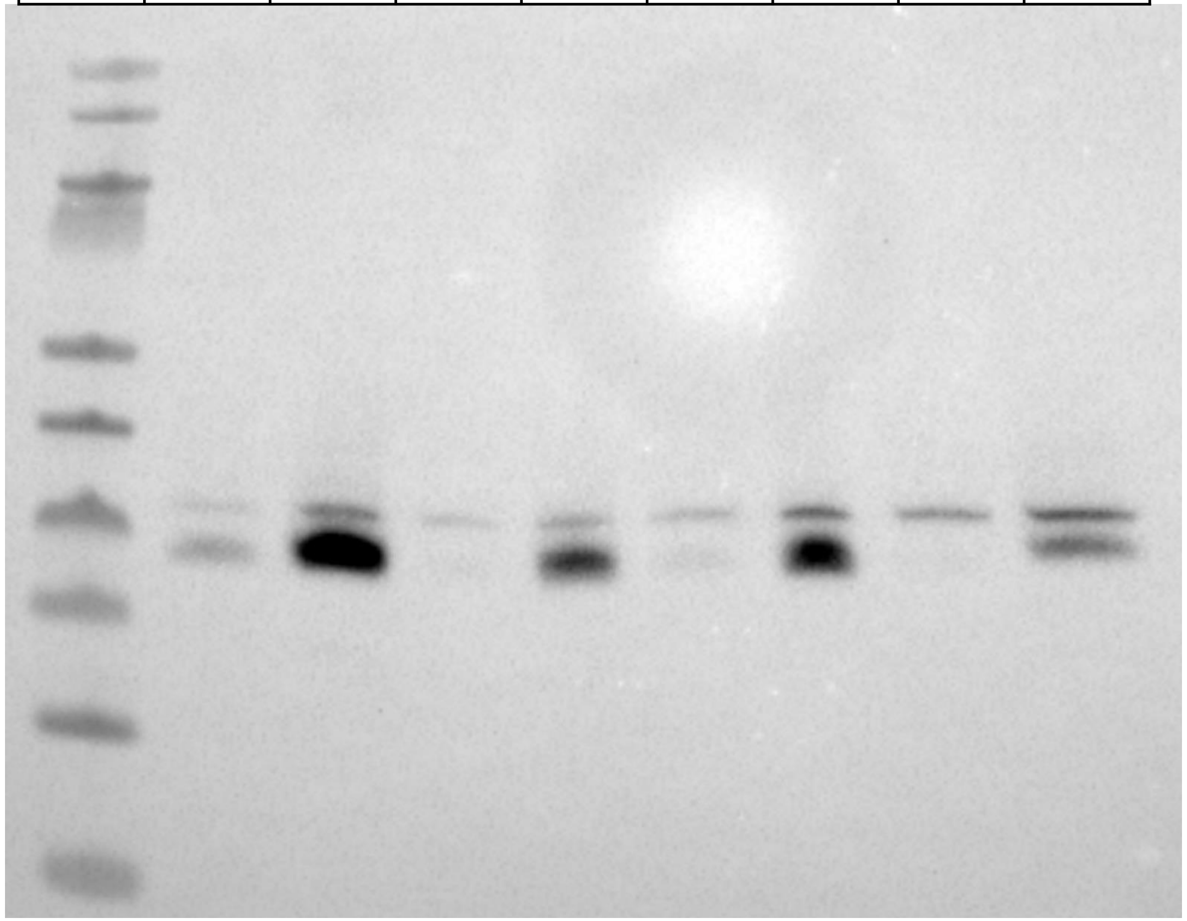


40 µg protein/lane
4-12% nuPAGE, turbo transfer
Anti-Bim (3C5) 1:1000 @ 4C, ON
Anti-Rat 1:1000 @ RT, 1h

| | Cas9 | Cas9 | 17 | 17 | 22 | 22 | 16.1 | 16.1 |
|---|---|---|---|---|---|---|---|---|
| 4HT | - | + | - | + | - | + | - | + |

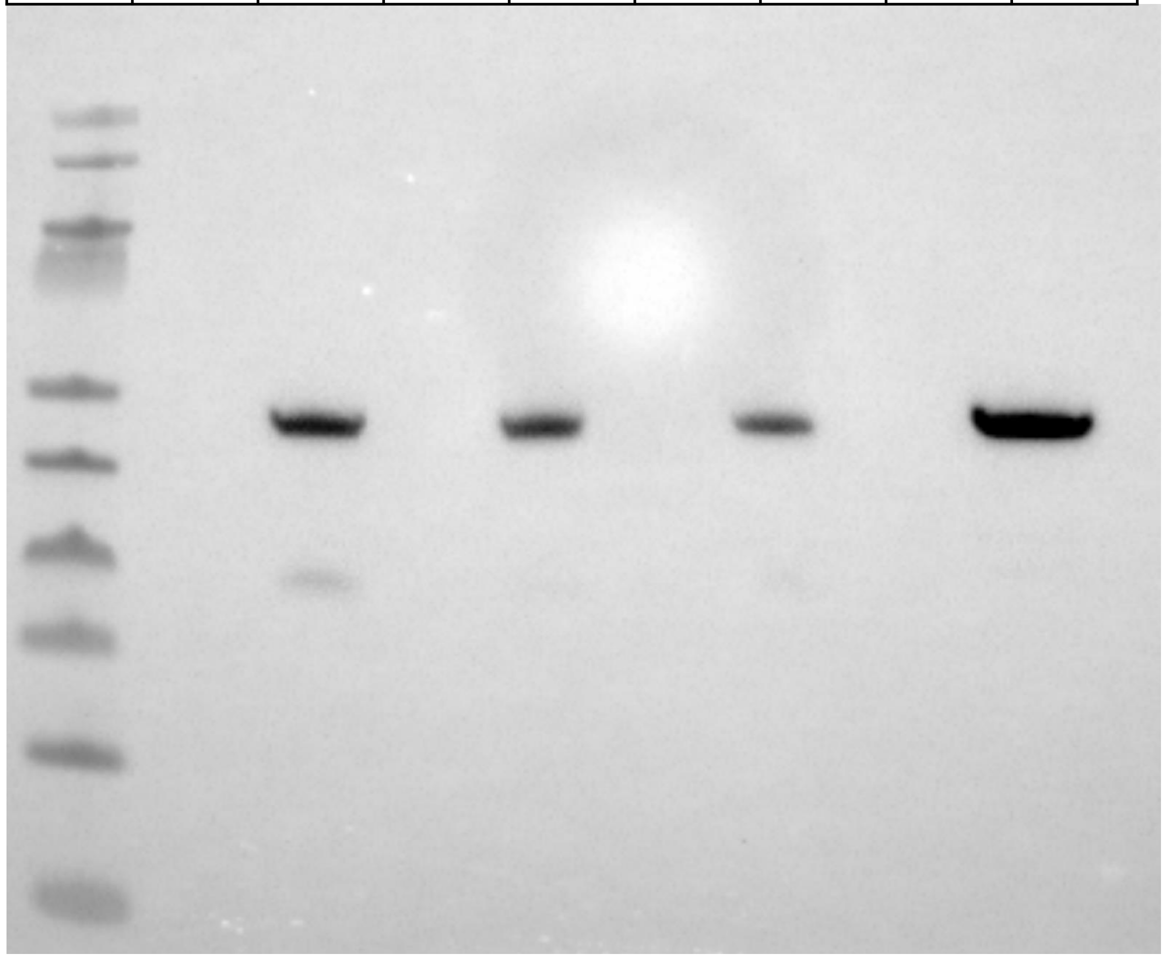


Anti-HA 1:2000 @ RT, 1h
Anti-Mouse 1:5000 @ RT, 1h

| | Cas9 | Cas9 | 17 | 17 | 22 | 22 | 16.1 | 16.1 |
|---|---|---|---|---|---|---|---|---|
| 4HT | - | + | - | + | - | + | - | + |

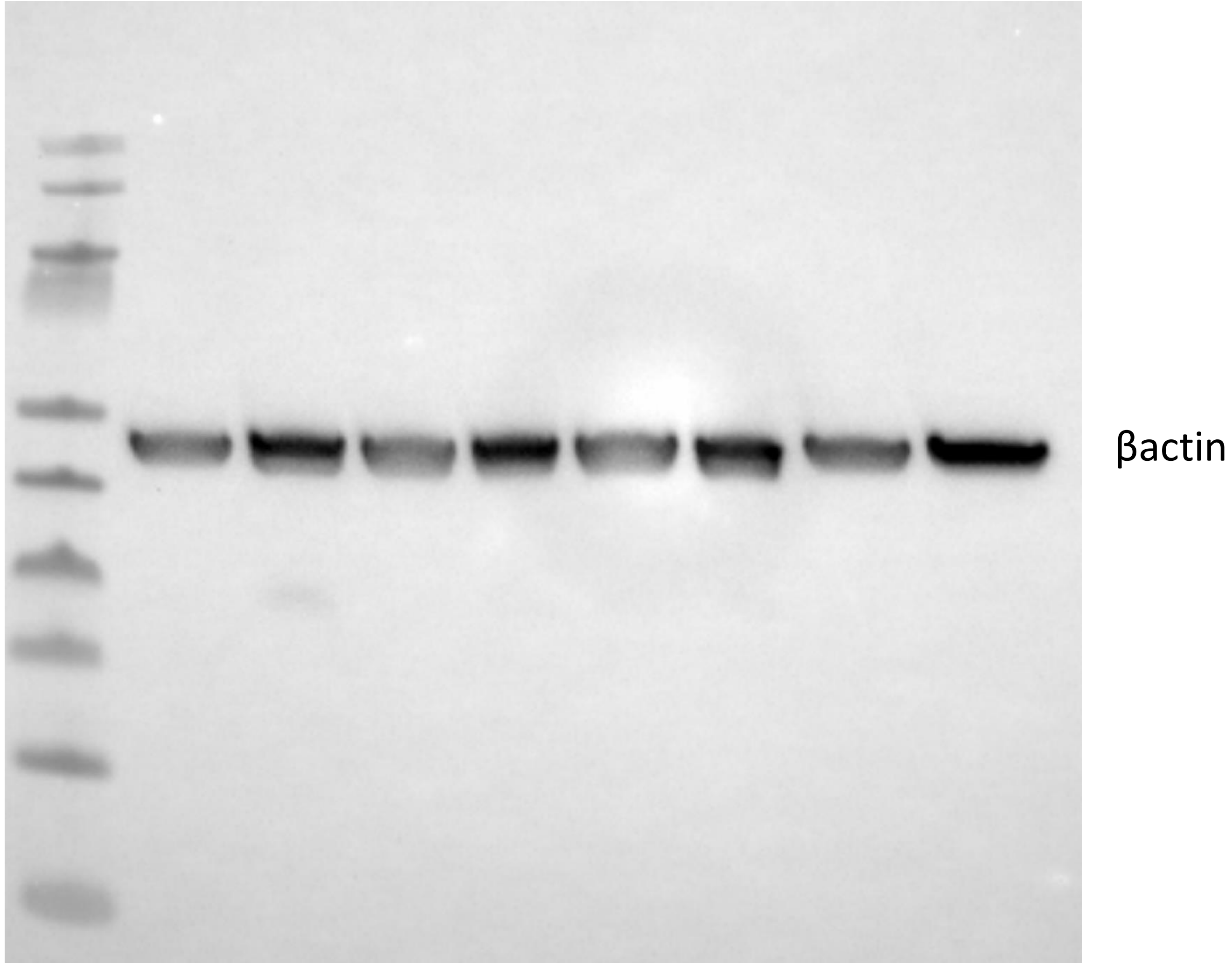


Anti-βactin 1:10000 @ RT, 0.5h
Anti-Mouse 1:5000 @ RT, 0.5h
