# Supplementary material for: Development of a novel therapy for systolic heart failure
Source: EMBO Mol Med. 2025 Aug 4;17(9):2332–53. doi: 10.1038/s44321-025-00284-6 (PMC12423297; doi:10.1038/s44321-025-00284-6)
Supplement: Supplementary file 6 — Source data Fig. 4 [file 44321_2025_284_MOESM6_ESM.zip › Figure 4 Original scans pdf/4E Scans.pdf]

	Cas9	Cas9	#17	#17	#22	#22	#16.1	#16.1
Tg (nm)	-	100	-	100	-	100	-	100

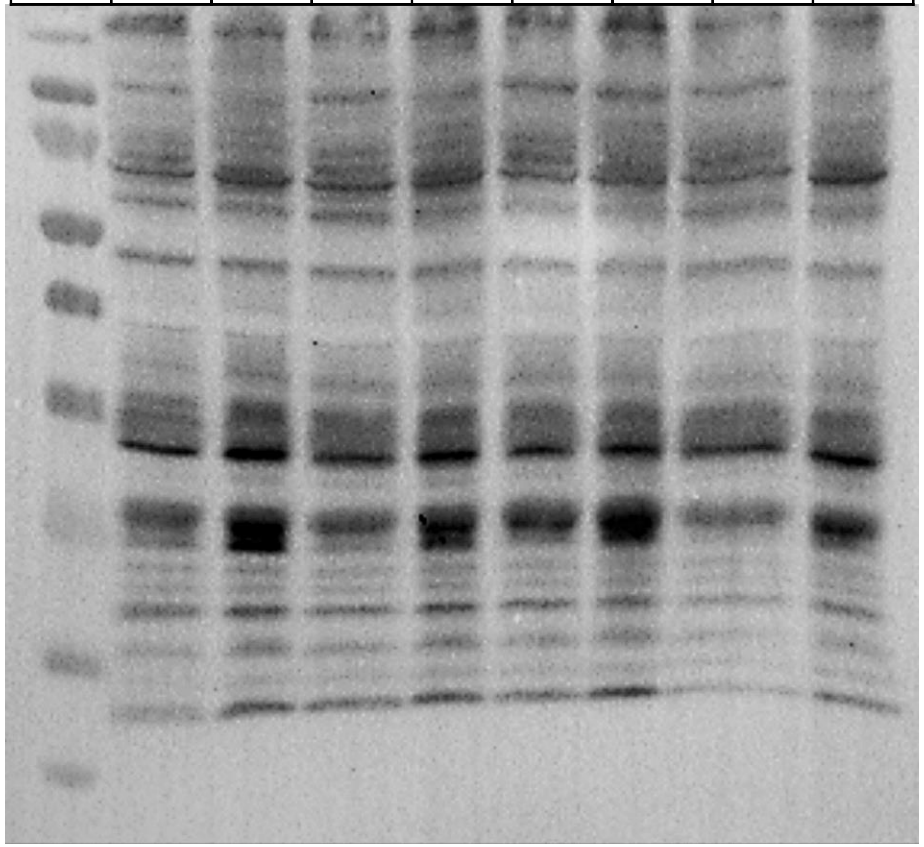

50 µg protein/lane
10-20% SDS PAGE, 25V 1.5h transfer
Anti-Bim (3C5) 1:1000 @ 4C, ON
Anti-Rat 1:1000 @ RT, 1h

	Cas9	Cas9	#17	#17	#22	#22	#16.1	#16.1
Tg (nm)	-	100	-	100	-	100	-	100

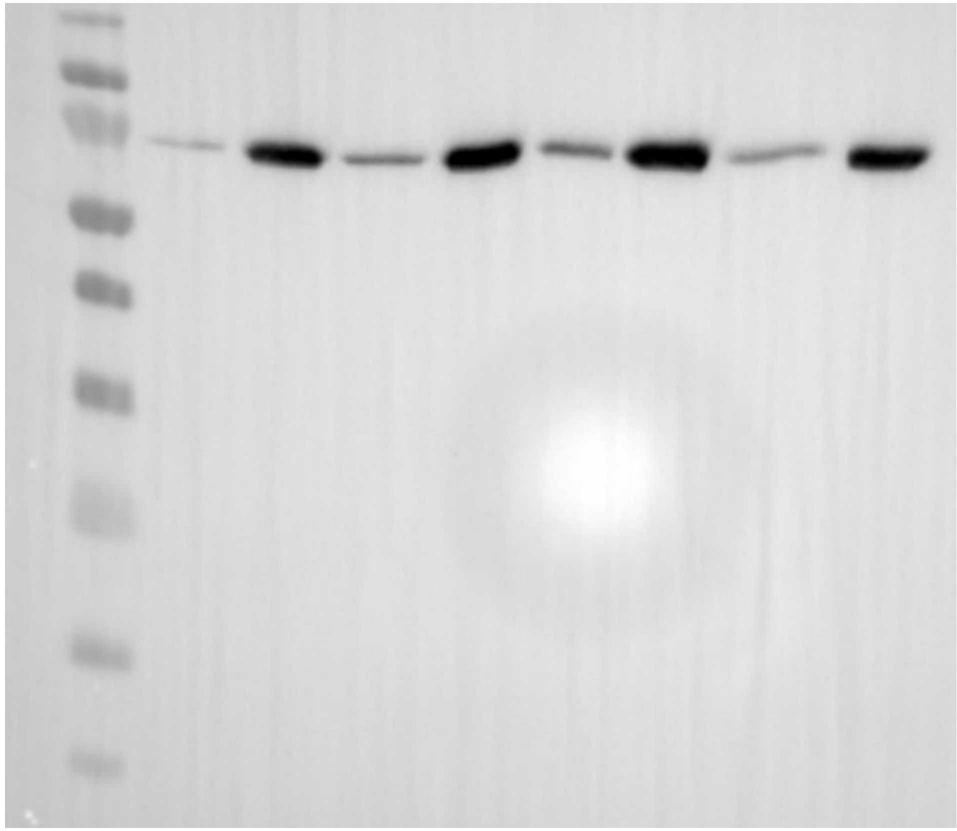

Bip

Anti-Bip 1:1000 @ 4C, ON
Anti-Mouse 1:5000 @ RT, 1h

	Cas9	Cas9	#17	#17	#22	#22	#16.1	#16.1
Tg (nm)	-	100	-	100	-	100	-	100

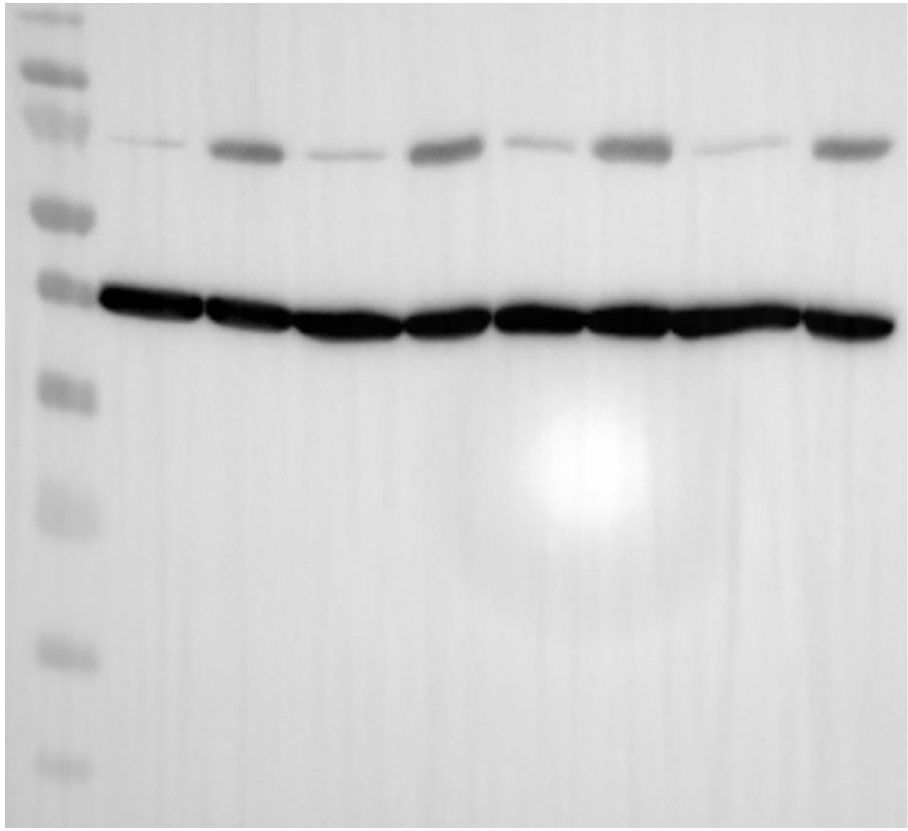

Anti-βactin 1:10000 @ RT, 1h
Anti-Mouse 1:5000 @ RT, 1h
