# Supplementary material for: Development of a novel therapy for systolic heart failure
Source: EMBO Mol Med. 2025 Aug 4;17(9):2332–53. doi: 10.1038/s44321-025-00284-6 (PMC12423297; doi:10.1038/s44321-025-00284-6)
Supplement: Supplementary file 7 — Source data Fig. 5 [file 44321_2025_284_MOESM7_ESM.zip › Figure 5 Original scans pdf/Fig 5B Scans.pdf]

4HT	-	+	+	+	-	-	+	+	-	-
SAH uM	-	-	10	1	10	1	-	-	-	-
SAM uM	-	-	-	-	-	-	10	1	10	1

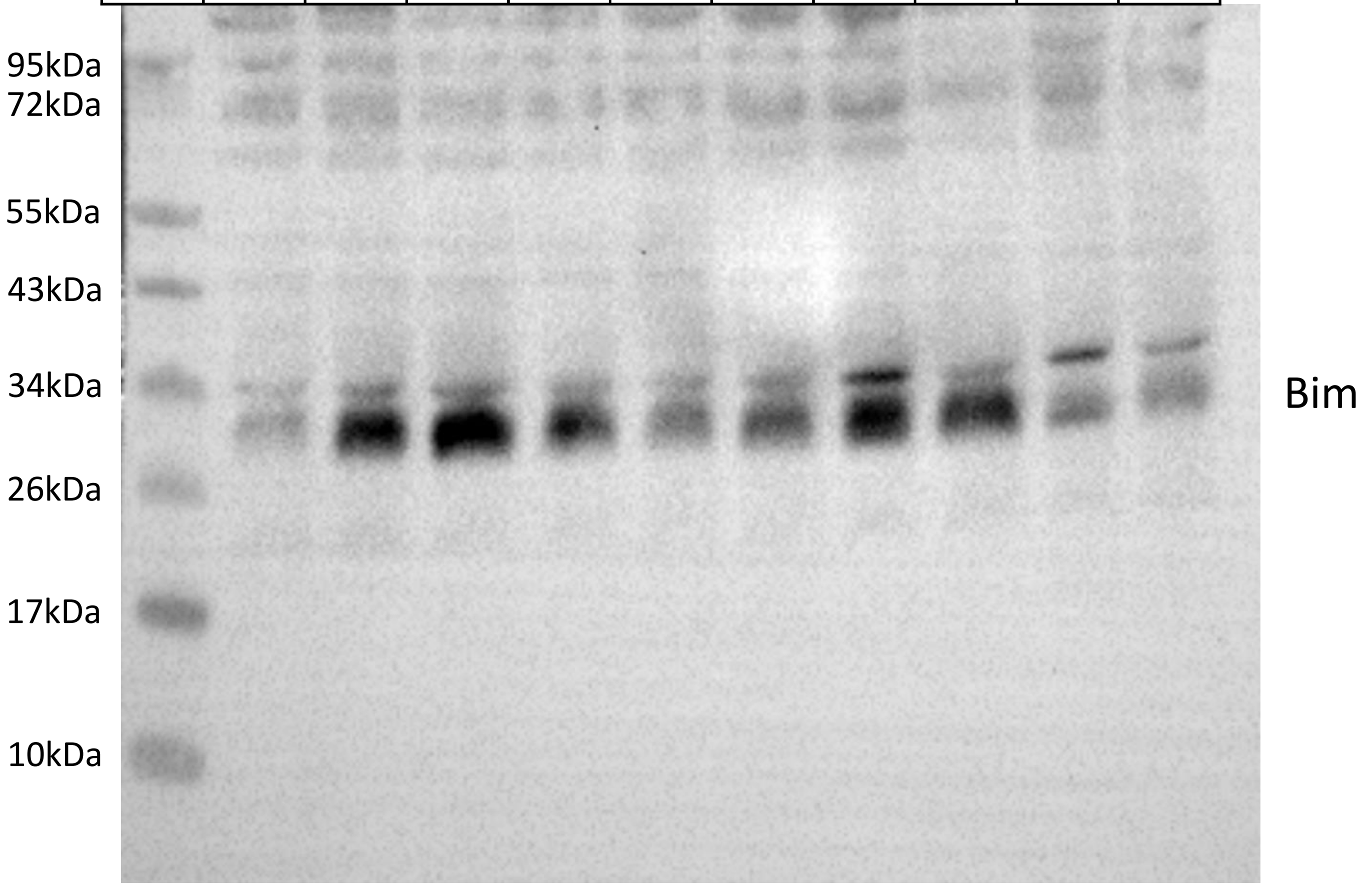

50 µg protein/lane
4-12% gel, 1h 25V transfer
Anti-Bim (3C5) 1:1000 @ 4C, ON
Anti-Rat 1:1000 @ RT, 1h

4HT	-	+	+	+	-	-	+	+	-	-
SAH uM	-	-	10	1	10	1	-	-	-	-
SAM uM	-	-	-	-	-	-	10	1	10	1

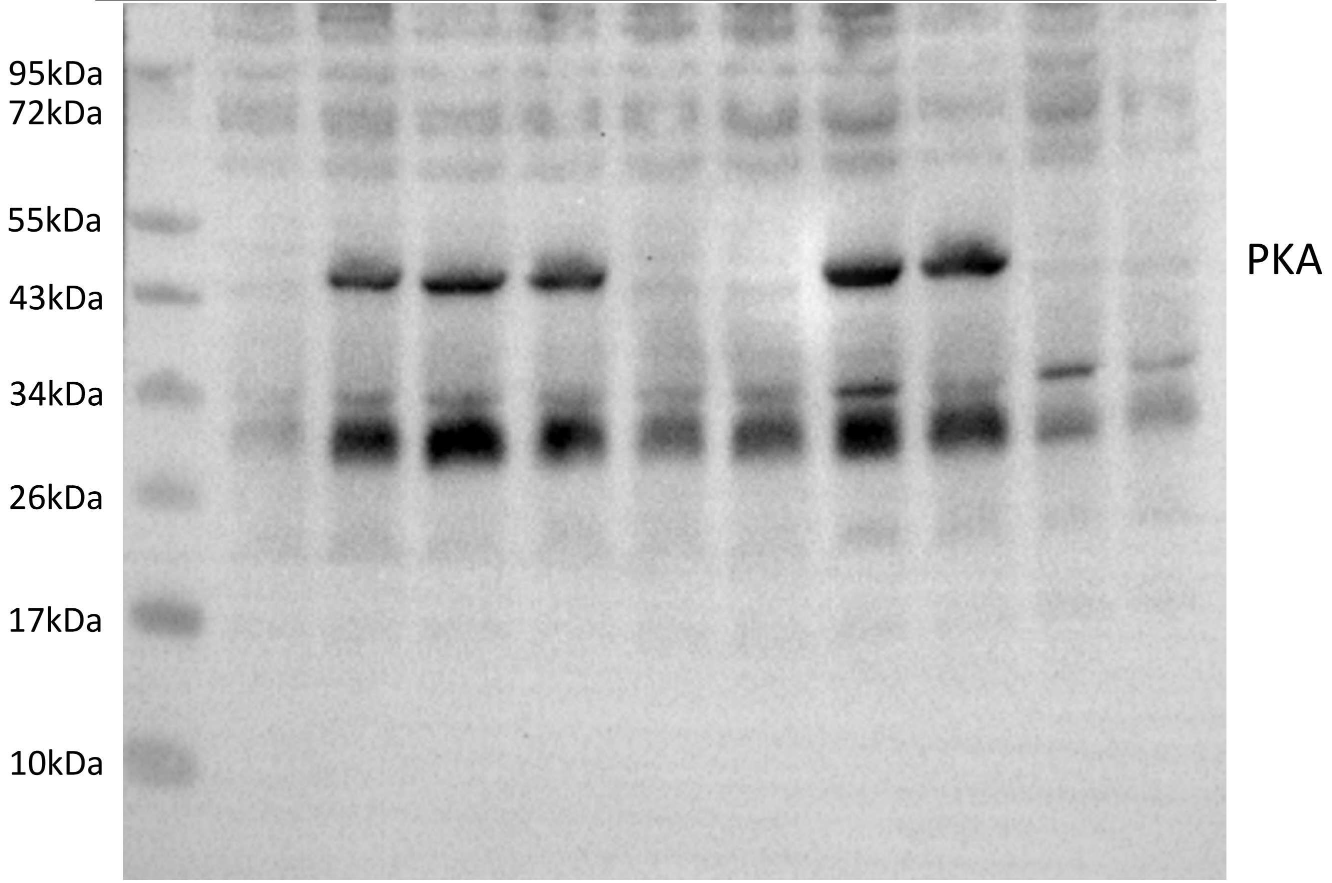

Anti-HA 1:5000 @ RT, 1h
Anti-Mouse 1:5000 @ RT, 1h

4HT	-	+	+	+	-	-	+	+	-	-
SAH uM	-	-	10	1	10	1	-	-	-	-
SAM uM	-	-	-	-	-	-	10	1	10	1

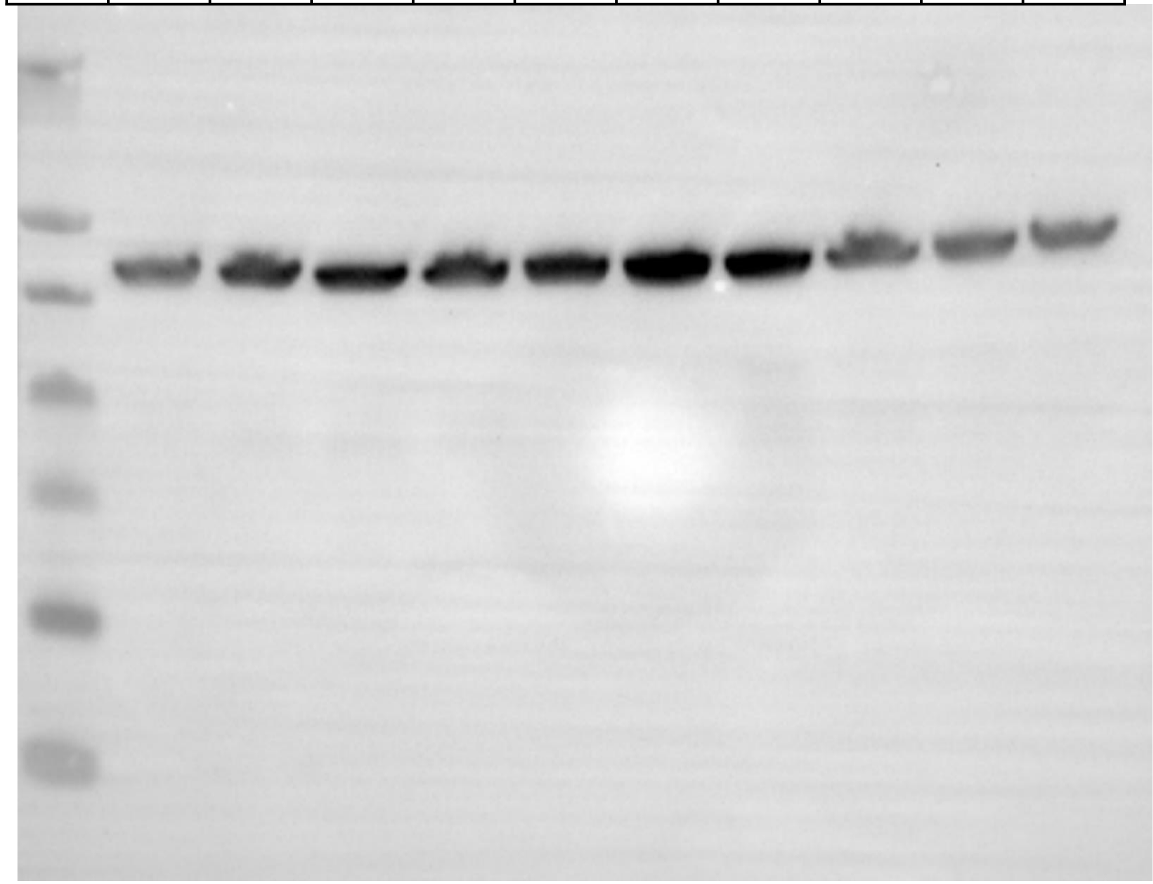

β-actin

Anti-β-actin 1:10000 @ RT, 1h
Anti-Mouse 1:5000 @ RT, 1h
