# Supplementary material for: Development of a novel therapy for systolic heart failure
Source: EMBO Mol Med. 2025 Aug 4;17(9):2332–53. doi: 10.1038/s44321-025-00284-6 (PMC12423297; doi:10.1038/s44321-025-00284-6)
Supplement: Supplementary file 7 — Source data Fig. 5 [file 44321_2025_284_MOESM7_ESM.zip › Figure 5 Original scans pdf/Fig 5C Scans.pdf]

	Cas9	Cas9	Cas9	Cas9	#17	#17	#17	#17
4HT	-	+	+	+	-	+	+	+
SAH 10 uM	-	-	10	-	-	-	10	-
SAM 10 uM	-	-	-	10	-	-	-	10

95kDa
72kDa
55kDa
43kDa
34kDa
26kDa
17kDa
10kDa

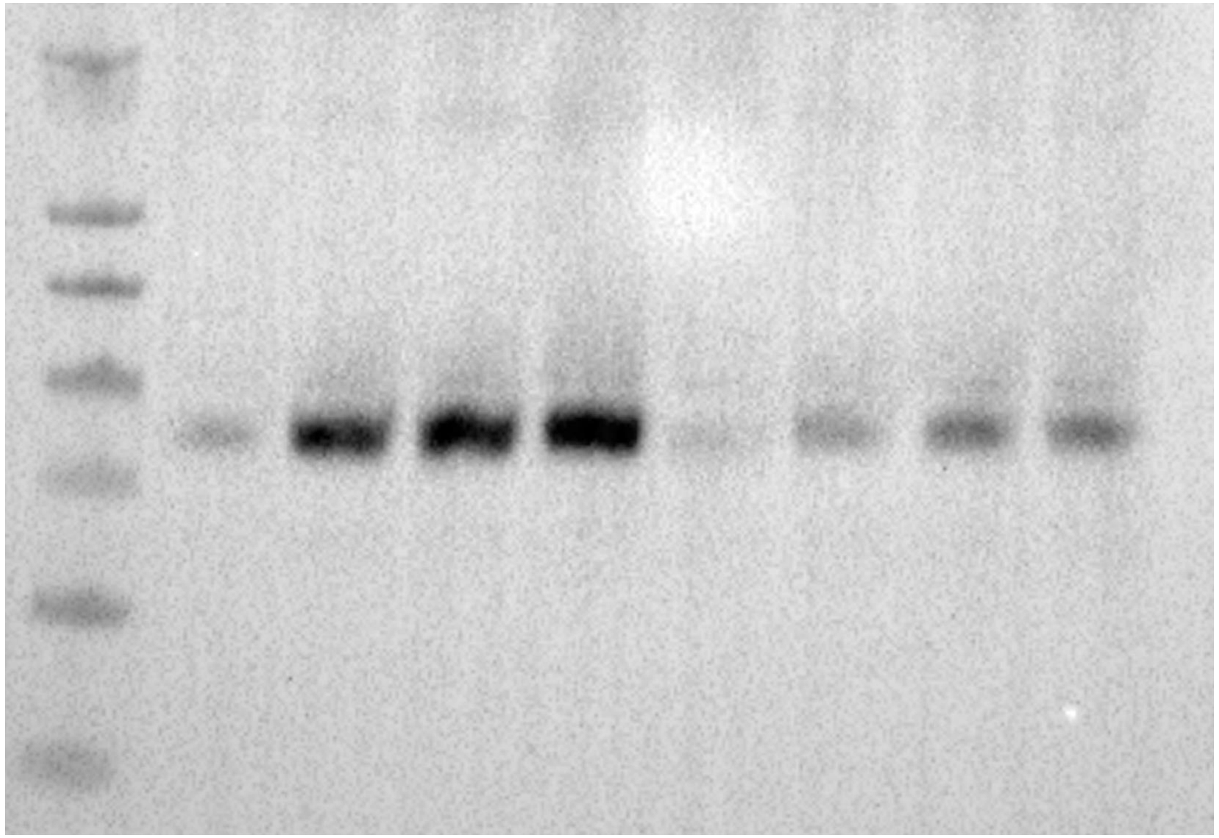

Bim

30 µg protein/lane
4-12% NuPAGE, 1h 25V transfer
Anti-Bim (3C5) 1:1000 @ 4C, ON
Anti-Rat 1:1000 @ RT, 1h

	Cas9	Cas9	Cas9	Cas9	#17	#17	#17	#17
4HT	-	+	+	+	-	+	+	+
SAH 10 uM	-	-	10	1	-	-	10	-
SAM 10 uM	-	-	-	10	-	-	-	10

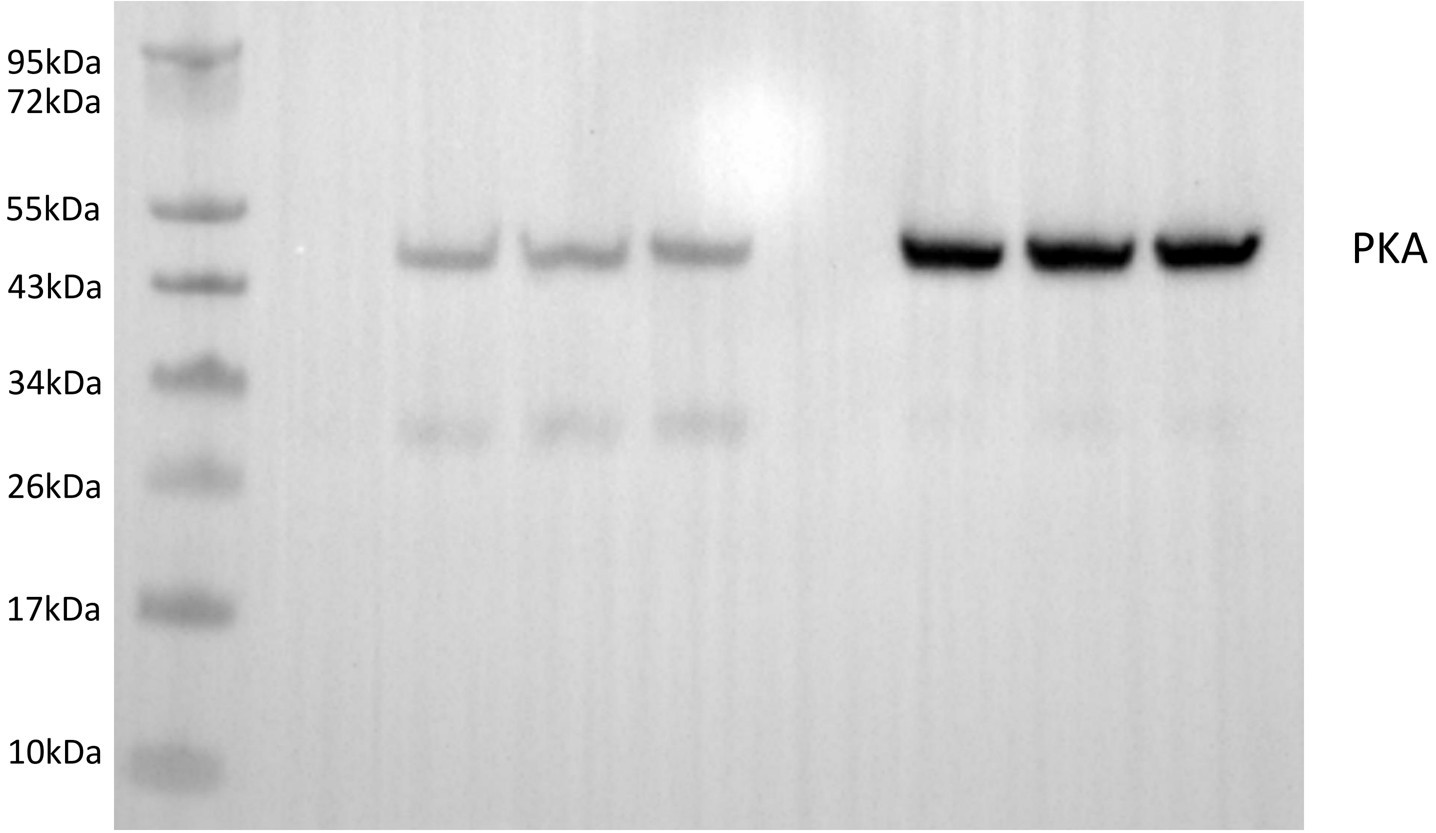

Anti-HA 1:5000 @ 4C, ON
Anti-Mouse 1:5000 @ RT, 1h

	Cas9	Cas9	Cas9	Cas9	#17	#17	#17	#17
4HT	-	+	+	+	-	+	+	+
SAH 10 uM	-	-	10	1	-	-	10	-
SAM 10 uM	-	-	-	10	-	-	-	10

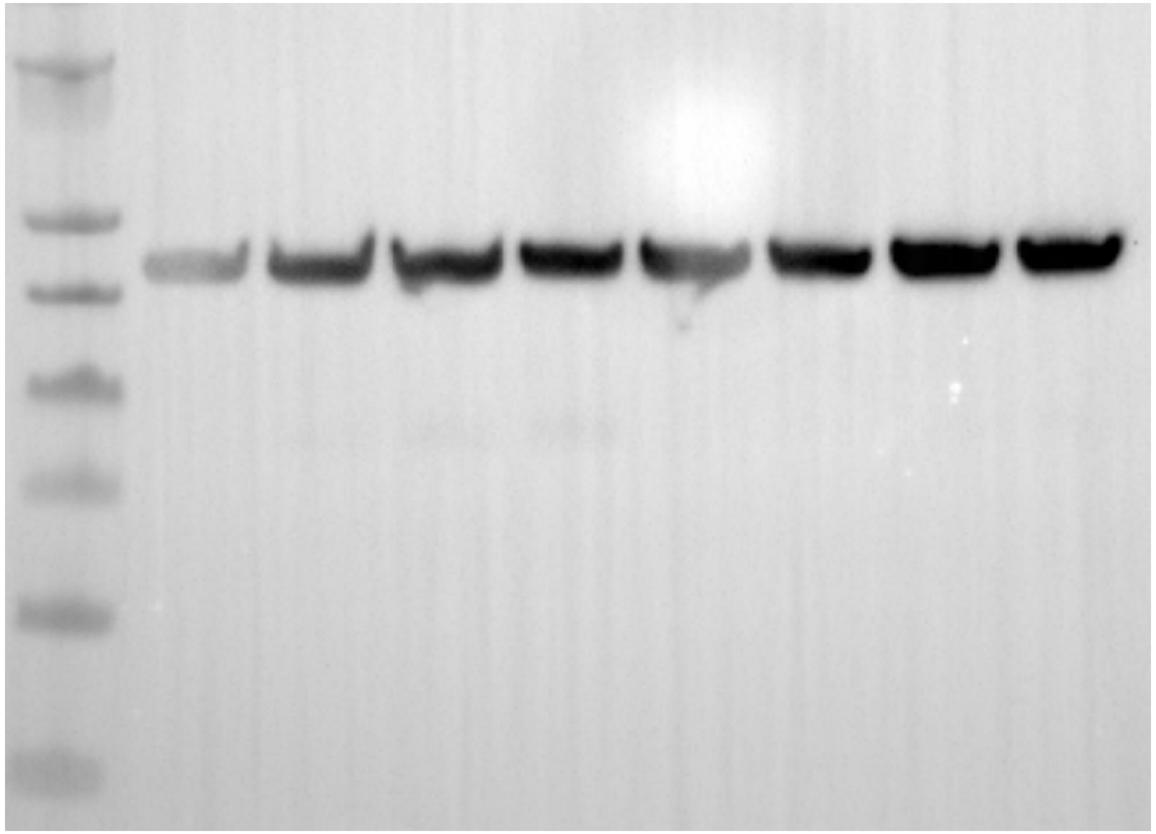

Anti-β-actin 1:10000 @ RT, 1h
Anti-Mouse 1:5000 @ RT, 1h
