# Supplementary material for: Development of a novel therapy for systolic heart failure
Source: EMBO Mol Med. 2025 Aug 4;17(9):2332–53. doi: 10.1038/s44321-025-00284-6 (PMC12423297; doi:10.1038/s44321-025-00284-6)
Supplement: Supplementary file 9 — Figure EV3 Source Data [file 44321_2025_284_MOESM9_ESM.zip › Fig EV 3 Original Scans pdf/EV Fig 3 Original scans.pdf]

Supplementary Fig 3 New)

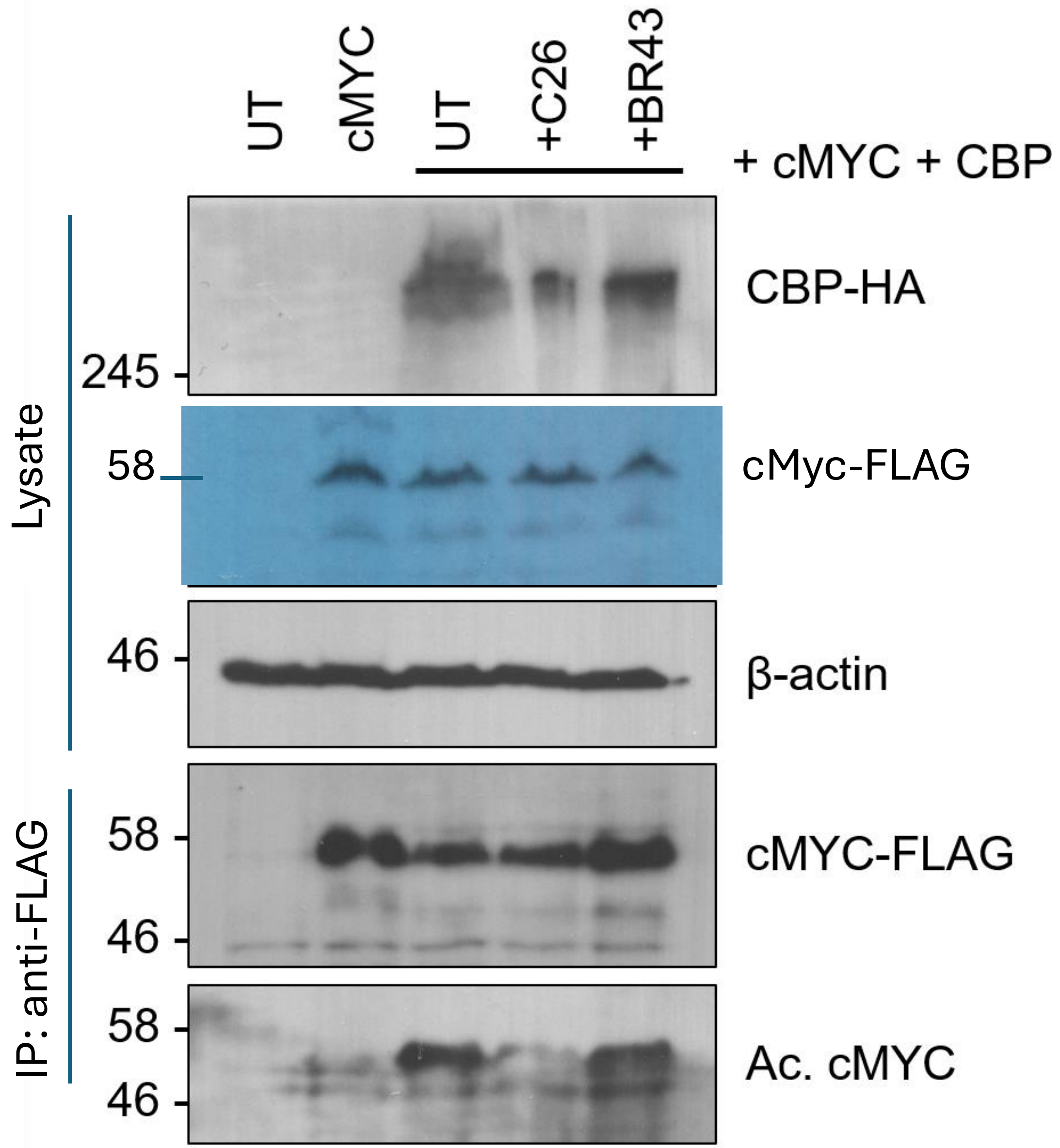

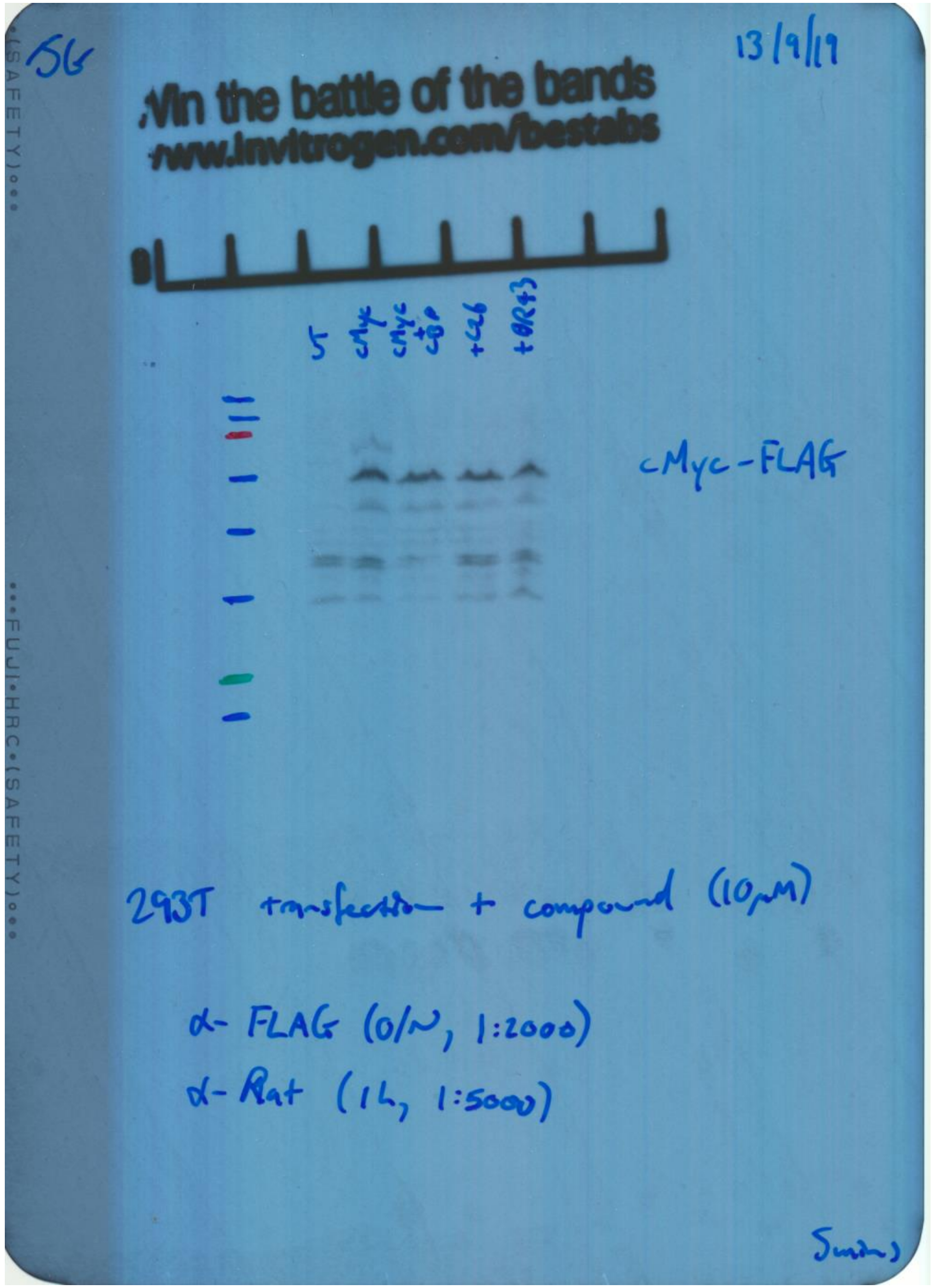
SG
13/9/19
Win the battle of the bands
www.invitrogen.com/bestabs
S
cMyc
cMyc + c80
+C26
+BR43
cMyc-FLAG
293T transfection + compound (10μM)
α- FLAG (O/N, 1:2000)
α- Rat (1h, 1:5000)
Sunday

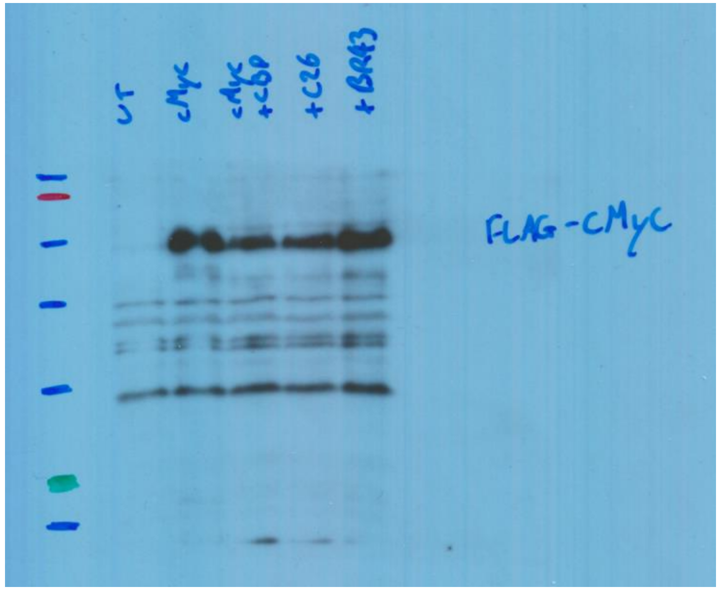
UT
cMyc
cMyc +CBP
+C26
FLAG-CMyC

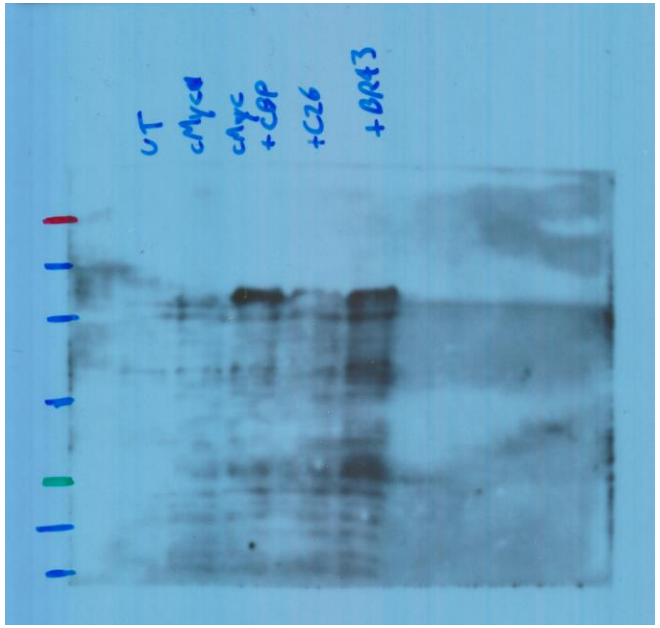
UT
cMyc
cMyc
+CBP
+C26

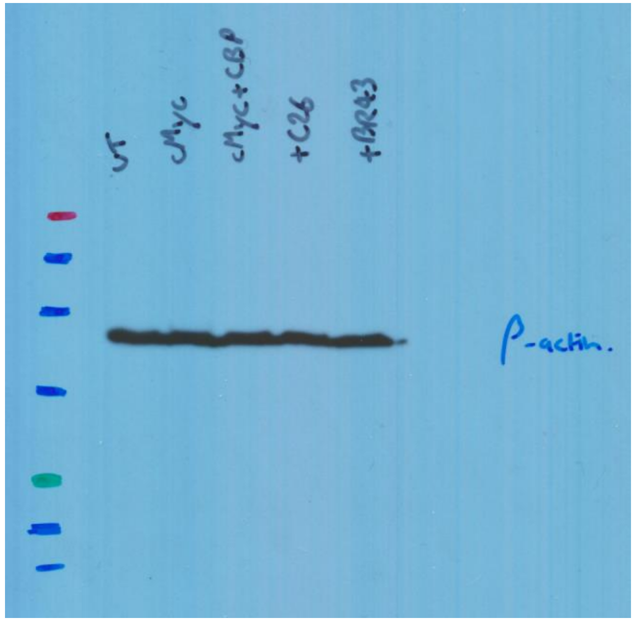
UT
cMyc
cMyc+CBP
+C26
+BR43
β-actin.

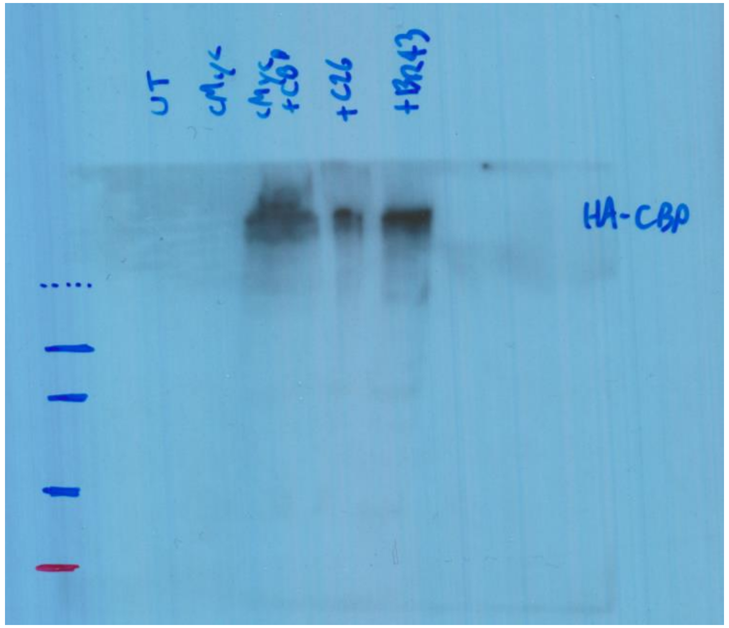
UT
HA-CBP
