# Supplementary material for: Development of a novel therapy for systolic heart failure
Source: EMBO Mol Med. 2025 Aug 4;17(9):2332–53. doi: 10.1038/s44321-025-00284-6 (PMC12423297; doi:10.1038/s44321-025-00284-6)
Supplement: Supplementary file 11 — Expanded View Figures [file 44321_2025_284_MOESM11_ESM.pdf]

Expanded View Figures

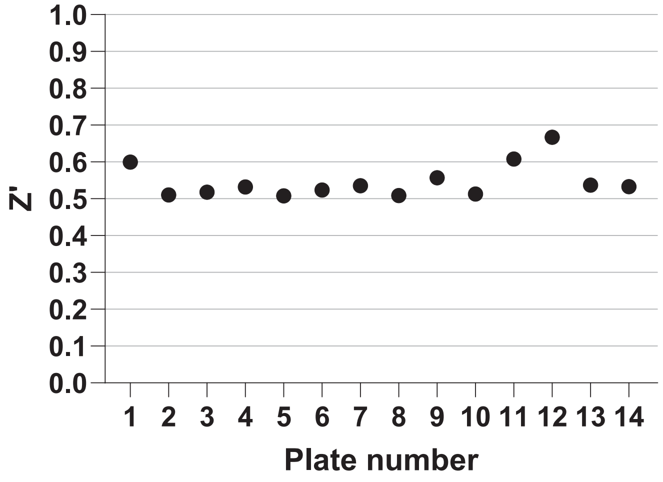

Figure EV1. The high-throughput screen.

The HTS shows that Z′ value exceeded 0.4 for all 14 assay plates. The Z′ value provides an indication of how robust a HTS assay is, whereby Z′ > 0.4 (dashed line) indicates cell-based assay robustness and suitability for HTS.

 © The Author(s)

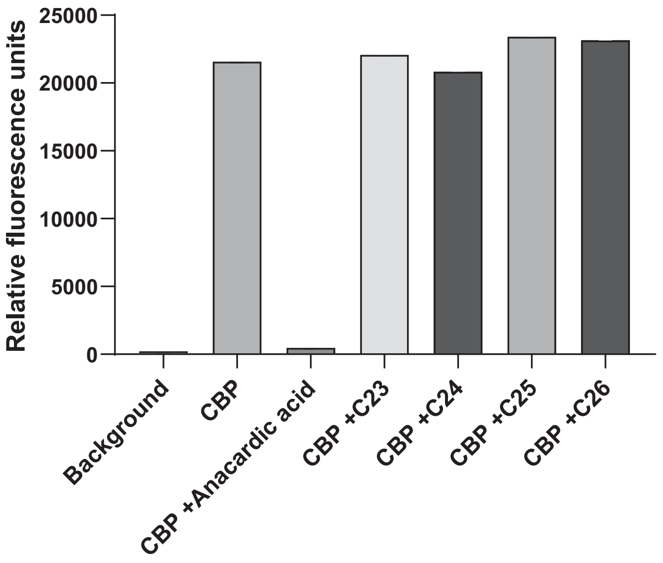

Figure EV2. Histone acetyltransferase (HAT) assay showing the drug-like compounds did not have HAT-inhibitor activity.

The HAT assay was performed using a HAT assay kit (Cat #56100, Active Motif, Carlsbad, CA 92008, USA), using HAT-inhibitor anacardic acid as a control. Each compound was tested at 10 μM concentration as per manufacturer's instructions. Source data are available online for this figure.

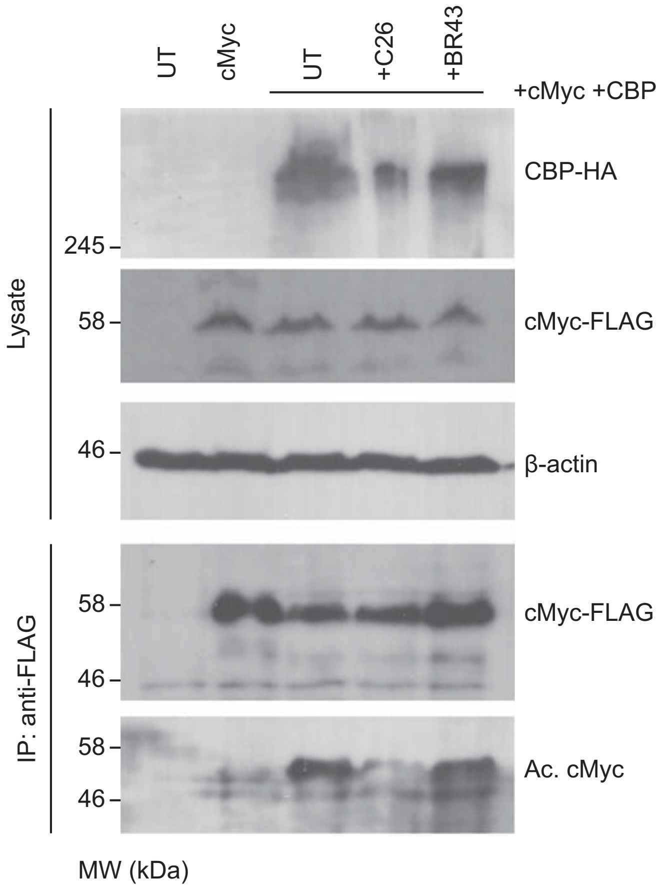

Figure EV3. The drug-like compounds do not inhibit c-Myc acetylation by CBP.

HEK293T cells were transfected with FLAG-c-Myc with or without HA-tagged CBP. Cells co-expressing CBP and c-Myc were left either untreated or treated with 10 mM of C26 or BR43 and analysed c-Myc was pulled down with anti-FLAG beads and analysed by Western blots with anti-FLAG and with anti-acetyl lysine antibodies. Source data are available online for this figure.

 © The Author(s)

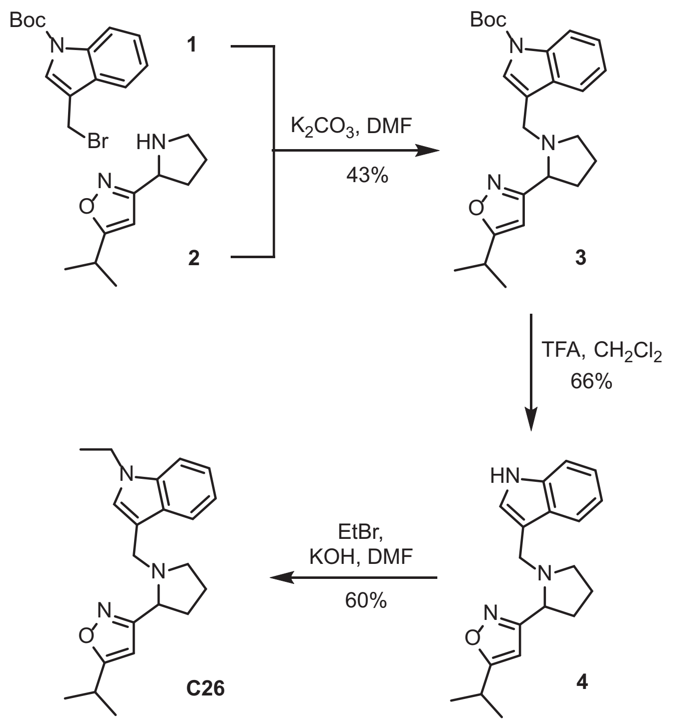

Figure EV4. Synthesis of C26.

The convergent strategy involved N-alkylation of 3-(pyrrolidine-2-yl) isoxazole (2) using bromomethylindole (1) to yield compound 3. This was subsequently subjected to Boc-deprotection to yield compound 4, which was N-alkylated further using ethyl bromide to get C26.

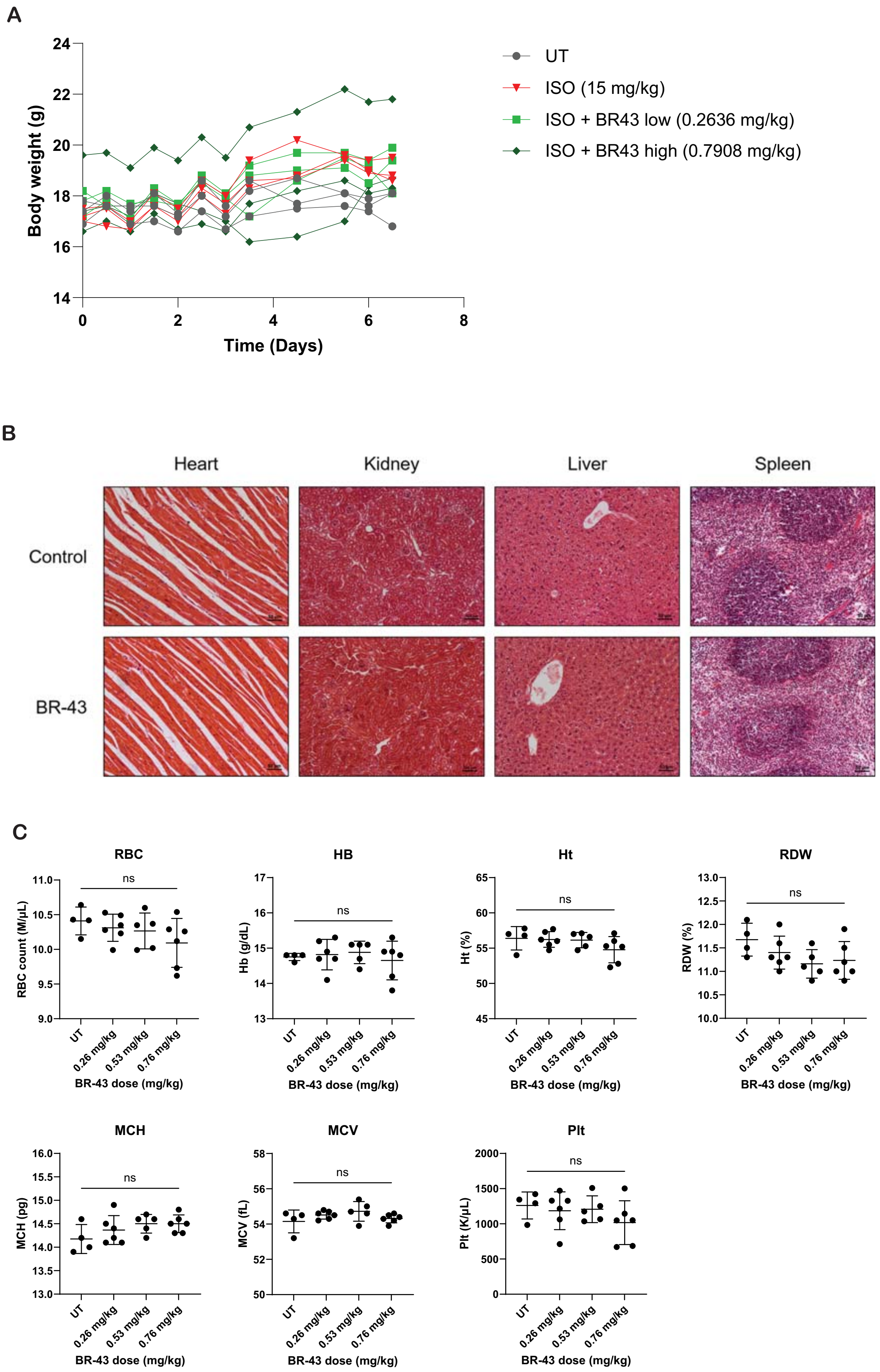
A
Body weight (g)
Time (Days)
UT
ISO (15 mg/kg)
ISO + BR43 low (0.2636 mg/kg)
ISO + BR43 high (0.7908 mg/kg)
B
Heart
Kidney
Liver
Spleen
Control
BR-43
C
RBC
RBC count (M/μL)
HB
Hb (g/dL)
Ht
Ht (%)
RDW
RDW (%)
MCH
MCH (pg)
MCV
MCV (fL)
Plt
Plt (K/μL)
ns
UT
0.26 mg/kg
0.53 mg/kg
0.76 mg/kg
BR-43 dose (mg/kg)

 © The Author(s)

Figure EV5. In vivo safety studies of BR43.

(**A**) C57B/6 were given PBS (UT control) or isoproterenol (ISO) using Alzet minipumps for a period of 24 h and subsequently given BR43 (I.P) for seven consecutive days and body weight was monitored. (**B**) H&E staining of various tissues at the end of 7 days and comparisons were made between control (UT) and Iso+BR43 high (0.79 mg/kg). (**C**) Various dozes of BR43 were injected (I.P) for 7 consecutive days and blood samples were analysed for various blood parameters as shown. Error bars ± SD, $n = 4$-8 animals in each group, one-way ANOVA with Tukey's multiple comparison test. Source data are available online for this figure.

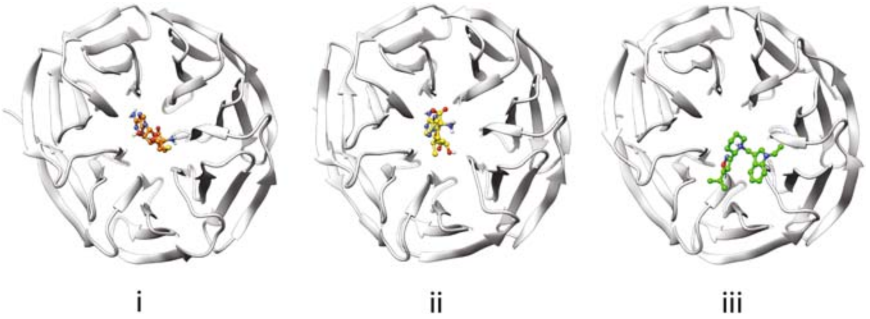

Figure EV6. SAH and SAM could induce Bim expression through Wdr3.

Ribbon diagram of the first beta barrel domain of Wdr3 complexed with (i) SAH, (ii) SAM and (ii) BR43. Ligands are presented in ball-and-stick representation, with carbon atoms coloured orange (SAH), yellow (SAM) and green (BR43), respectively, with nitrogen, oxygen and sulphur coloured blue, red and yellow, respectively.

 © The Author(s)
